# Joint Clinical Practice Guideline on Benzodiazepine Tapering: Considerations When Risks Outweigh Benefits

**DOI:** 10.1007/s11606-025-09499-2

**Published:** 2025-06-17

**Authors:** Emily Brunner, Chwen-Yuen A. Chen, Tracy Klein, Donovan Maust, Maryann Mazer-Amirshahi, Marcia Mecca, Deanna Najera, Chinyere Ogbonna, Kiran F. Rajneesh, Elizabeth Roll, Amy E. Sanders, Brett Snodgrass, Amy VandenBerg, Tricia Wright, Maureen Boyle, Amanda Devoto, Sarah Framnes-DeBoer, Bethea Kleykamp, Janette Norrington, Dawn Lindsay, Clinical Guideline Committee (CGC) Members, Emily Brunner, Chwen-Yuen A. Chen, Tracy Klein, Donovan Maust, Maryann Mazer-Amirshahi, Marcia Mecca, Deanna Najera, Chinyere Ogbonna, Kiran F. Rajneesh, Elizabeth Roll, Amy E. Sanders, Brett Snodgrass, Amy VandenBerg, Tricia Wright, ASAM Staff and Contractors, Maureen Boyle, Amanda Devoto, Sarah Framnes-DeBoer, Bethea Kleykamp, Janette Norrington, Dawn Lindsay

**Affiliations:** 1 Hazelden Betty Ford Foundation, Minneapolis, MN USA; 2 Stanford University, Stanford, CA USA; 3 Washington State University, Vancouver, WA USA; 4 University of Michigan, Ann Arbor, MI USA; 5 Georgetown University, Washington, DC USA; 6 Yale School of Medicine, New Haven, CT USA; 7 MedStar Emergency Physicians, Olney, MD USA; 8 Kaiser Permanente San Jose Medical Center, San Jose, CA USA; 9 The Ohio State University, Columbus, OH USA; 10 Yukon Kuskokwim Health, Bethel, AK USA; 11 Sunday Health, Vienna, VA USA; 12 Baptist Health System, Memphis, TN USA; 13 University of Michigan College of Pharmacy, Ann Arbor, MI USA; 14 University of California San Francisco, San Francisco, CA USA; 15 American Society of Addiction Medicine, Rockville, MD USA; 16 University of Maryland, Baltimore, MD USA

**Keywords:** Benzodiazepine tapering, Clinical guideline, Shared decision-making

## Abstract

**Description:**

The American Society of Addiction Medicine (ASAM) has partnered with nine other medical societies and professional associations representing a wide range of clinical settings and patient populations to provide guidance on evidence-based strategies for tapering benzodiazepine (BZD) medication across a variety of settings.

**Methods:**

The guideline was developed following modified GRADE methodology and clinical consensus process. The process included a systematic literature review as well as several targeted supplemental searches. The clinical practice guideline was revised based on external stakeholder review.

**Recommendations:**

Key takeaways included the following: Clinicians should engage in ongoing risk–benefit assessment of BZD use/tapering, clinicians should utilize shared decision-making strategies in collaboration with patients, clinicians should not discontinue BZDs abruptly in patients who are likely to be physically dependent and at risk of withdrawal, clinicians should tailor tapering strategies to each patient and adjust tapering based on patient response, and clinicians should offer patients adjunctive psychosocial interventions to support successful tapering.

**Supplementary Information:**

The online version contains supplementary material available at 10.1007/s11606-025-09499-2.

## EXECUTIVE SUMMARY

### Purpose

The following medical and professional societies partnered to develop and disseminate this *Joint Clinical Practice Guideline on Benzodiazepine Tapering* (hereafter referred to as the Guideline):

- American Academy of Family Physicians (AAFP)
- American Academy of Neurology (AAN)
- American Academy of Physician Associates (AAPA)
- American Association of Nurse Practitioners (AANP)
- American Association of Psychiatric Pharmacists (AAPP)
- American College of Medical Toxicology (ACMT)
- American College of Obstetricians and Gynecologists (ACOG)
- American Geriatrics Society (AGS)
- American Psychiatric Association (APA)
- American Society of Addiction Medicine (ASAM)

This Guideline provides information on evidence-informed and consensus-based strategies to help clinicians determine whether tapering benzodiazepine (BZD) medications may be appropriate for a given patient and, if so, how to taper them. This Guideline applies to adult patients who have been taking BZDs regularly and may be at risk of physical dependence. Note that physical dependence is an expected outcome associated with BZD use and is distinct from BZD use disorder. Additional considerations for patients with substance use disorder (SUD) are discussed separately in the section “Patients with Benzodiazepine and Other Substance Use Disorders.” Clinicians in palliative and end-of-life care settings are not the intended audience for this Guideline.

### Background

BZDs are approved by the US Food and Drug Administration (FDA) to treat a wide range of conditions, including anxiety and panic disorders, social phobia, insomnia, and seizures, and are commonly prescribed. They are important therapeutic tools. However, use of these medications is associated with an increased risk of adverse events, including falls, motor vehicle accidents, cognitive impairment, delirium, overdose, and death, particularly when BZDs are used in combination with central nervous system (CNS) depressants such as alcohol or opioids.^1–3^ The risk–benefit balance of BZD prescribing may shift over time as patients age and their physical or mental health conditions and other prescribed medications change. Because physical dependence is an expected outcome of BZD use, discontinuation can be challenging. When BZDs are used regularly, abrupt discontinuation (i.e., stopping the medication without a taper) or precipitous dose decreases can lead to serious and potentially life-threatening withdrawal symptoms.
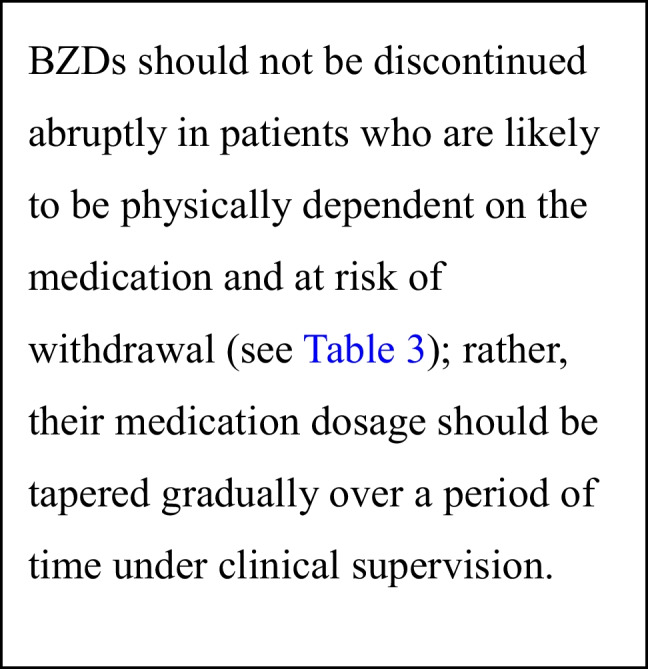


### Key Takeaways

This Guideline aims to assist clinicians in helping patients who have developed physical dependence safely taper BZDs while minimizing withdrawal symptoms and associated risks. The recommendations in this Guideline address considerations for tapering, level of care, tapering strategies, withdrawal management, and specific patient populations.

Due to the paucity of evidence addressing BZD tapering strategies, the majority of the recommendations in this Guideline are based on clinical consensus. Three recommendation statements are based on low-quality evidence from ten studies (see “Summary of Recommendations”).

The following are ten key takeaways of this Guideline for adult patients who have been taking BZDs regularly and may be at risk of physical dependence:

1. Clinicians should base clinical recommendations regarding continued BZD prescribing on ongoing assessment of the risks and benefits of continued BZD use as well as those of tapering/discontinuation (see Table 2). Tapering is generally indicated when the risks of continuing BZD medication outweigh the benefits.
  - Clinicians should conduct more frequent risk–benefit assessments of continued BZD prescribing for patients who:
    i. Are concomitantly taking opioid medication
    ii. Have an SUD
    iii. Have additional risk factors for adverse effects, such as co-occurring physical conditions (e.g., obstructive sleep apnea) or mental health conditions (e.g., bipolar spectrum disorder)
  - Clinicians should use caution if utilizing urine drug screen immunoassays for BZDs due to known limitations.
  - Clinicians should consider the maternal–fetal dyad when assessing the risks and benefits of continued BZD prescribing in patients who are pregnant.
  - Clinicians should taper BZDs in most older adults (i.e., ≥ 65 years) unless there are compelling reasons for continuation.
2. Clinicians should consider approaches to BZD tapering in collaboration with patients and their care partners utilizing shared decision-making strategies.
3. Clinicians should not discontinue BZDs abruptly in patients who are likely to be physically dependent and at risk of withdrawal symptoms (see Recommendation 2 “Rationale” and associated evidence summary).
  - Physical dependence can develop within weeks and is heterogeneous across patients^4^ (see Table 3).
4. Although most patients can complete BZD tapering in outpatient settings, clinicians should consider inpatient or medically managed residential care when patient presentation indicates significant risk that cannot be safely managed in outpatient care.
5. Clinicians should design the tapering strategy to minimize harms from both continued BZD use and the tapering process, such as withdrawal symptoms and recurrence of symptoms for which the BZD was originally prescribed. The initial pace of the BZD taper should generally include dose reductions of 5 to 10% every 2–4 weeks. The taper should typically not exceed 25% every 2 weeks.
  - Patients who have been taking lower doses for a relatively short period of time (e.g., < 3 months) may be able to taper more quickly.
  - The goal of tapering may be discontinuing the BZD medication or reducing the BZD dose to the point where the risks no longer outweigh the benefits.
6. Clinicians should tailor tapering strategies to each individual patient and adjust tapering based on patient response.
  - Clinicians can consider transitioning patients without contraindications to a comparable dose of a longer-acting BZD medication for the taper (see Appendix B).
  - Clinicians should monitor patients for the emergence of BZD withdrawal signs and symptoms with each dose reduction. If significant signs or symptoms emerge, the taper should be slowed or paused.
  - Some patients experience significant withdrawal symptoms, even with gradual tapering, and should be offered slower tapering as needed.
  - In some cases, maintaining a patient on a lower dose may be sufficient to reduce the current risks such that they no longer outweigh the benefits.
7. Clinicians should offer patients undergoing BZD tapering adjunctive psychosocial interventions (e.g., cognitive behavioral therapy (CBT), cognitive behavioral therapy for insomnia (CBT-I)) to support successful tapering (see Recommendation 10 “ Rationale” and associated evidence summary).
8. Clinicians should provide concurrent treatment for any co-occurring physical health conditions and psychiatric disorders, including SUDs, that could interfere with the BZD taper.
9. Clinicians should employ harm reduction strategies—such as providing opioid overdose reversal medication (e.g., naloxone) to those concomitantly taking opioids or otherwise at risk of opioid overdose, connecting patients to local resources, and providing patient education—based on each individual patient’s risks.
10. It may take months to years to fully taper off BZDs, particularly if patients have been taking a high dose for an extended period of time.

Additional considerations for patients with BZD use disorders are discussed separately in the section “Patients with Benzodiazepine and Other Substance Use Disorders.”

### Recognizing Implementation Challenges

The recommendations in this Guideline are relevant to millions of people in the USA. In 2023, nearly 24 million people in the USA reported use of a BZD, with approximately 20 million reporting use as prescribed.^4^ In 2018, an estimated 50% of patients dispensed oral BZD received them for 2 months or longer.^5^ Long-term BZD prescribing is also common among older adults, for whom this and other guidelines recommend avoiding BZD use. As clinicians and healthcare systems begin to implement this Guideline, they may identify a large population of patients who would benefit from tapering. We recognize healthcare systems are already overburdened and significant workforce challenges may limit the capacity to manage BZD tapering at scale. Clinicians and healthcare systems may need to identify strategies for prioritizing patients who are at the highest risk of experiencing BZD-related harms in the short term. See “Implementing this Guideline” for further discussion.

Guiding principles for implementation of recommendations from the *Joint Clinical Practice Guideline on Benzodiazepine Tapering*

1. The recommendations in this Guideline are intended to support patient-centered care. Many complex factors influence decision-making related to BZD tapering, and there is significant heterogeneity in patient response to tapering. This Guideline should be implemented to allow flexibility in response to diverse clinical circumstances.
2. Healthcare systems, payers, policymakers, and clinicians should avoid misapplying this Guideline beyond its intended use in ways that may lead to unintentional harms for patients.
3. Clinicians should develop tapering strategies collaboratively with patients, tailoring strategies to each patient’s risks, needs, and preferences, and adjusting strategies based on a patient’s response.
4. Healthcare systems and policymakers should carefully consider how to best leverage existing healthcare resources to meet the needs of the potentially large population for whom BZD tapering may be indicated.
5. Physical dependence is an expected result of ongoing use of prescribed BZDs and distinct from SUD. Clinicians should not presume that patients with physical dependence have an SUD. Patients with SUD should be managed appropriately (see “Patients with Benzodiazepine and Other Substance Use Disorders”), with referrals for specialty treatment as necessary.

## INTRODUCTION

### Purpose

The following medical and professional societies partnered to develop and disseminate this *Joint Clinical Practice Guideline on Benzodiazepine Tapering* (hereafter referred to as the Guideline):

- American Academy of Family Physicians (AAFP)
- American Academy of Neurology (AAN)
- American Academy of Physician Associates (AAPA)
- American College of Medical Toxicology (ACMT)
- American Association of Nurse Practitioners (AANP)
- American Association of Psychiatric Pharmacists (AAPP)
- American College of Obstetricians and Gynecologists (ACOG)
- American Geriatrics Society (AGS)
- American Psychiatric Association (APA)
- American Society of Addiction Medicine (ASAM)

This Guideline provides information on evidence-informed and consensus-based strategies to help clinicians determine whether tapering benzodiazepine (BZD) medications may be appropriate for a given patient and, if so, how to taper them. This Guideline applies to adult patients who have been taking BZDs regularly and may be at risk of physical dependence and withdrawal (see Table 2).

### Background

BZDs are approved by the US Food and Drug Administration (FDA) to manage a wide range of conditions, including acute conditions (e.g., panic and acute anxiety, alcohol withdrawal, seizures) and common chronic conditions (e.g., anxiety disorders, primary insomnia). These medications are commonly prescribed and represent important therapeutic tools; however, data on long-term safety and efficacy are limited. BZD use is associated with an increased risk of adverse events including falls, motor vehicle accidents, cognitive impairment, delirium, overdose, and death, particularly when BZDs are used in combination with CNS depressants such as alcohol or opioids.^1–3^

Since 2000, fatal overdoses involving BZDs have increased nearly tenfold, often involving the combination of opioids and BZDs.^1^ Although prescribing rates for BZDs have fallen since the most recent peak in 2013, in the 2023 National Survey on Drug Use and Health (NSDUH), 9.1% of US adults reported use of BZDs in the past year, with more than 15% of those reporting BZD misuse.^5,6^ Between 1996 and 2013, the number of adults filling BZD prescriptions increased from 4.1 to 5.6%, while the total quantity of BZD prescriptions filled more than tripled, from 1.1 to 3.6 kg lorazepam equivalents per 100,000 adults.^7^ Over this time, emergency department (ED) visits related to BZDs also tripled, and BZD-related overdose deaths quadrupled.^1,8^ Since 2013, however, BZD prescriptions dispensed from outpatient and mail-order pharmacies have fallen by approximately 33%.^6^

Despite potential harms, long-term use of BZDs (i.e., ≥ 120 days) is common.^9,10^ Long-term BZD use is associated with an increased risk of physical dependence and withdrawal and ongoing risk of adverse events such as falls, motor vehicle accidents, and cognitive impairment.^3,11–13^ Evidence also suggests that use of BZDs is associated with increased suicide risk, although the mechanism for this association is not well understood.^12,13^ The risk–benefit balance for continued BZD use may shift over time, and stopping can be challenging because physical dependence develops with regular use. It should be noted that physical dependence is an expected outcome associated with the use of prescribed BZDs and is distinct from substance use disorders (SUDs; see Box 1). Older adults (i.e., ≥ 65 years old) have the highest BZD prescription rates and are at higher risk of experiencing adverse events related to BZD use.^9,14^ Some older adults have taken BZDs continuously for decades.^9,14,15^ In some instances, use has been so prolonged that the original reason for the BZD prescription may be unclear.

**Box 1:** Physical Dependence Versus Substance Use Disorder

Physical dependence is a biological phenomenon that develops in response to repeated use of a medication. In the case of BZDs, physical dependence results from downregulation of BZD receptors and/or adaptations in the response of the receptor. Physical dependence is an expected consequence of ongoing use of BZD. Conversely, SUD is a chronic disease associated with functional changes to the brain circuits that mediate stress, decision-making, and behavior reinforcement. In addition to physical dependence, SUD is associated with specific criteria, including impaired control over use of the substance and continued use despite adverse consequences.^16^ Genetic, psychosocial, and environmental factors influence the development and manifestations of SUD. A review of NSDUH data estimated that only 1.5% of people who use BZDs met criteria for a BZD use disorder.^17^ Patients who use BZDs and are physically dependent on the medication are far more common than patients who have a BZD use disorder.	

Safe tapering of BZDs can be clinically complex because rapid dosage reductions may precipitate acute withdrawal, which can be life-threatening. Patients are also at risk of recurrence and exacerbation of the symptoms for which BZDs were initially prescribed (e.g., anxiety, seizures, insomnia) and destabilization. This Guideline was motivated, in part, by patients reporting harms associated with too rapid tapering/discontinuation of BZD medications. Inadequate tapering strategies may push patients to the illegal drug market, where counterfeit pills laced with fentanyl and other highly potent synthetic opioids (HPSOs) are common, presenting an increased risk of overdose and overdose death.^18^ This Guideline aims to help clinicians in diverse practice settings determine whether and how to taper BZD medications.

#### Intersection with the Opioid Overdose Epidemic

Co-prescribing of BZDs and opioids quadrupled between 2003 and 2015 in ambulatory care settings, with data from 2014 to 2016 indicating over one-third of BZD prescriptions were co-prescribed with opioids.^14,19^ In addition, some individuals may concomitantly take BZDs and opioids to augment the effects of both substances.^20^

Given that both BZDs and opioids cause CNS depression, concomitant use increases the risk of adverse events, including fatal and nonfatal overdose.^21–23^ In 2021, 13.7% of overdose deaths in the USA involving opioids also involved BZDs, and nearly 88% of overdose deaths involving BZDs also involved opioids.^1^

### Scope of Guideline

This Guideline focuses on whether and how to taper BZD medications, exploring considerations for assessing risks and benefits of continued prescribing, partnering with patients, level of care considerations, and tapering strategies, including management of withdrawal symptoms. It pertains to patients who have been taking BZDs regularly and are at risk of physical dependence and withdrawal. This Guideline also addresses population-specific considerations, including for patients co-prescribed BZDs and opioids, patients with SUD, patients with other psychiatric disorders, older adults, and pregnant and lactating patients.

This Guideline is not applicable to patients who are prescribed BZDs but are not taking them regularly (Box 2). It is also not applicable to patients who are prescribed BZDs for a short period of time (e.g., for under 2 weeks for the management of agitation, acute anxiety, or alcohol withdrawal). Considerations related to initiation of BZDs, ongoing management of BZD prescriptions, and management of underlying conditions are beyond the scope of this Guideline. Additionally, although non-BZD sedative–hypnotic medications such as barbiturates and Z-drugs (i.e., eszopiclone, zaleplon, and zolpidem) have similar mechanisms of action to BZDs and may pose similar risks, they are beyond the scope of this Guideline. Finally, considerations for BZD tapering in children and adolescents (i.e., < 18 years old) are beyond the scope of this Guideline.

A glossary of terms, abbreviations, and acronyms used in this Guideline can be found in Supplementary Materials 1 and 2.

**Box 2:** Note of Caution: Avoid Misapplication of this Guideline

As observed upon the release of the 2016 CDC Guideline for Prescribing Opioids for Chronic Pain, clinical practice guidelines (CPGs) can have unintended impacts on clinical decision-making.^24^ Misapplication of the 2016 CDC opioid recommendations led some prescribers to abruptly discontinue pain medications without first developing a plan for safe tapering with their patients.^24^ This unintended response put patients at risk of withdrawal and potential transition to illegally obtained opioids while failing to address their underlying pain symptoms.^25,26^ Abrupt discontinuation of BZDs confers similar and additional risks: rapid BZD dose reduction can cause life-threatening withdrawal symptoms such as seizures and delirium, as well as potential destabilization of existing mental health conditions, especially in those who have been taking long-term BZDs and at higher doses.^2,20,27^ As highlighted in this Guideline, BZDs should not be discontinued abruptly in patients who are likely to have developed physical dependence. Clinicians and healthcare systems should carefully consider how to meet the needs of patients requiring BZD tapering, including those who experience significant challenges during the tapering process (see “Implementing this Guideline”).	

### Intended Audience

The intended audience of this Guideline is clinicians—including behavioral health professionals, physicians, advanced practice providers, and pharmacists—who prescribe BZDs or provide or support treatment for indications for which BZDs are often prescribed. This Guideline is relevant to clinicians who practice in diverse settings such as primary care offices, ambulatory care clinics for a broad range of specialty clinicians, EDs, hospitals, and outpatient and residential addiction and mental health treatment settings. Some recommendations only apply to specific settings (e.g., inpatient treatment, medically managed settings), as indicated in the narrative. Clinicians in palliative and end-of-life care are not the intended audience for this Guideline. This Guideline may be useful for healthcare administrators, insurers, and policymakers who implement policies related to medical practice. However, this Guideline is not intended to be a source of rigid laws, regulations, or policies related to BZD prescribing or tapering. The recommendations contained in this Guideline support flexible, patient-centered care.

### Qualifying Statement

This Guideline is intended to aid clinicians in their clinical decision-making and patient management (see Box 3). It strives to identify and define clinical decision-making junctures that meet the needs of most patients in most circumstances. Clinical decision-making should consider the quality and availability of expertise and services in the community wherein care is provided. The recommendations in this Guideline reflect the consensus of an independent committee (see “Methodology”) convened beginning January 2023. This Guideline will be updated periodically as clinical and scientific knowledge advances.

Prescribed courses of treatment described in this Guideline are most effective if the patient understands and adheres to the recommendations. Clinicians should make every effort to promote patients’ understanding of and adherence to prescribed and recommended treatment services to improve outcomes.

This Guideline aims to set the standard for best clinical practice by providing recommendations for the appropriate care of patients tapering from BZDs in diverse settings. Patients should be informed of the risks, benefits, and alternative treatment options and welcomed as active parties to shared decision-making. In circumstances in which this Guideline is used to inform regulatory or payer decisions, the central goal should be improvement in quality of care. Recommendations in this Guideline do not supersede any federal or state regulations.

**Box 3:** Intended Use of the *Joint Guideline on Benzodiazepine Tapering*

This Guideline is: • Primarily intended for clinicians who prescribe BZDs in diverse settings such as primary care, specialty care, EDs, and hospitals settings • Applicable to patients aged 18 years and older who are taking BZDs regularly and may be at risk of physical dependence • A clinical tool for supporting individualized, patient-centered care in BZD tapering • Intended to promote flexible and patient-centered care and shared decision-making This Guideline is not: • Intended for clinicians who prescribe BZDs in palliative and end-of-life care settings • Applicable to patients taking BZDs for a short time (e.g., less than 2 weeks) or irregularly (e.g., as needed) • A replacement for clinical judgment or individualized, patient-centered care • Intended to be applied as inflexible standards of care or lead to the rapid tapering or abrupt discontinuation of BZDs • Intended to suggest a one-size-fits-all approach to BZD tapering • A law, regulation, or policy that dictates clinical practice • Focused exclusively on patients with SUDs	

## METHODOLOGY

ASAM’s Quality Improvement Council (QIC) and Clinical Practice Guideline Methodology and Oversight Committee (CPG-MOS) oversaw the development of this Guideline. The FDA provided guidance on the content and development of the Guideline but did not dictate the content. The QIC, working with partner professional societies and the FDA, oversaw the appointment of a Clinical Guideline Committee (CGC) comprised of clinicians representing ten medical and professional societies with broad subject matter expertise across medicine, psychiatry, and pharmacology. A panel of individuals who have lived experience with BZD tapering (the Patient Panel) provided input during the development of the Guideline.

The following key clinical questions were addressed in the systematic literature review:

1. What is the efficacy and/or safety of tapering strategies for BZDs?
2. What factors influence the outcomes of BZD tapering and should be monitored?
3. How can shared decision-making and patient-centered health care be utilized to support the effectiveness and safety of BZD tapering?

These questions were used to develop a Population, Intervention, Comparator, Outcome (PICO) framework for identifying relevant research literature to answer each of the key clinical questions.

- Population: Adults who have been using one or more BZD medication for at least 2–4 weeks, including those with benzodiazepine use disorder
- Intervention: two types of interventions were considered:
  - Interventions that promote the successful discontinuation of BZD use
  - Interventions that manage withdrawal symptoms when discontinuing BZDs
- Comparator: alternative interventions, treatment as usual, placebo, or active control condition
- Outcome: BZD cessation or dose reduction, BZD withdrawal severity, recurrence or rebound of BZD-indicated conditions (e.g., insomnia, anxiety), sleep problems, cognition, mood, quality of life and patient satisfaction, global functioning, study attrition, other substance use, and adverse events

A systematic literature review that considered risks and benefits of BZD tapering as well as patient values and preferences was conducted to inform the development of recommendations. The Grading of Recommendations, Assessment, Development, and Evaluation (GRADE) method was used to develop recommendations in areas with sufficient evidence.^28^ A modified Delphi process was used to develop clinical consensus statements when evidence was lacking.^29^ As very little high-quality evidence was found to directly inform the clinical questions, this strategy allowed for the inclusion of guidance in areas with limited evidence.

The full draft Guideline was released for public comment in June 2024. The CGC reviewed all public comments and revised the document to address identified concerns. The final document was approved and/or endorsed by the respective boards of all partner organizations.

The detailed Methodology can be found in Supplementary Material 3. A list of CGC members, their areas of expertise, and conflict of interest disclosures are available in Supplementary Material 4. GRADE Evidence to Decision (EtD) tables are available in Supplementary Material 5.

## INTERPRETING RECOMMENDATION STATEMENTS

Two pieces of information are included with each recommendation statement: certainty of evidence and strength of the recommendation. The certainty of evidence reflects the level of confidence—or certainty—in how closely the effect estimates reflect the true effect and, therefore, the extent to which the evidence can be relied upon when making recommendation decisions. Certainty of evidence was evaluated using the GRADE method using categories of high, moderate, or low. Consensus-based recommendations were labeled with “Clinical Consensus” rather than a certainty of evidence rating. The CGC graded the strength of each recommendation as strong or conditional based on the overall balance of risks and benefits, the certainty of the evidence on treatment effects, and patient preferences and values. The CGC worded recommendations to reflect the strength of the statement. For example, “clinicians should” indicates a strong recommendation, while “clinicians can consider” indicates a conditional recommendation (see Table 1).

**Table 1:** Recommendation Wording, Strength, and Interpretation

Strength	Recommendation wording	Interpretation^30^
Strong	“Clinicians should…” “Clinicians should not…”	Benefits clearly outweigh risks (or vice versa). Can apply to most patients in most circumstances
Conditional	“Clinicians can consider…”	Benefits are closely balanced with risks. Correct action may differ depending on patient values. Different clinical choices will be appropriate for different patients. Patient-centered decision-making should be the goal based on a patient’s needs, values, and preferences

This table explains how the recommendations in this Guideline are worded based on strength and how each type of recommendation (i.e., strong and conditional) should be interpreted

The systematic review identified 57 relevant articles. Few studies were identified that directly addressed many of the core topics within the Guideline. Due to the paucity of evidence, the majority of the recommendations in this Guideline are based on clinical consensus. Three recommendation statements are based on low-quality evidence from ten studies (see “Summary of Recommendations”). The remaining studies were considered by the CGC but were not used to inform specific recommendations. In the recommendation statement, the certainty of evidence is bolded and italicized when evidence was relied upon to make the recommendation. Recommendations based on the consensus of the CGC based on their clinical expertise, rather than direct evidence, are clearly labeled *Clinical Consensus*.

The clinical recommendation statements are accompanied by implementation considerations that provide guidance on how to implement the recommendations. These include important contextual and patient-centered factors to consider for clinical decision-making.
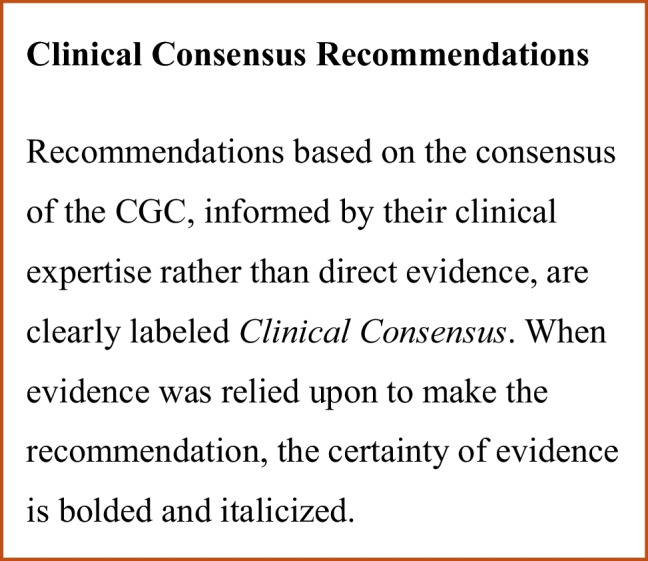


## SUMMARY OF RECOMMENDATIONS

**Recommendations for Considerations for Tapering Benzodiazepines**

1. Clinicians should ideally assess the risks and benefits of ongoing BZD prescribing at least every 3 months for each patient taking BZD medications (see Tables 2 and 3; *Clinical Consensus*, Strong Recommendation).
  a At a minimum, clinicians should assess the risks and benefits with each new BZD prescription or BZD prescription renewal (*Clinical Consensus*, Strong Recommendation).
  b Clinicians should review the information in the relevant prescription drug monitoring programs (PDMPs) as part of the risk–benefit assessment (*Clinical Consensus*, Strong Recommendation).

**Table 2:** Potential Benefits and Risks of Continued BZD Use and BZD Tapering

Potential benefits	Potential risks	
BZD use	BZD use	BZD taper
• Effectiveness in managing a patient’s mental and physical health condition(s) • Related functional improvements • Quality of life improvements*	• Oversedation, including consideration of use with other sedating medications, alcohol, or other drugs • Falls and related injuries • Memory and cognitive impacts • Motor vehicle accidents • Medical safety concerns (e.g., medication interactions) • Impacts on co-occurring mental and physical health conditions • Disrupted sleep patterns • Impacts on work and family responsibilities • Diversion • Substance use disorder • Overdose • Fetal harm • Suicidality	• Withdrawal symptoms, including severe or complicated withdrawal (e.g., seizures, delirium) • Recurrence of the condition for which BZD was prescribed • Impacts on co-occurring mental and physical health conditions • Protracted withdrawal • Return to illicit BZD use • Transition to illicit BZD use^†^

A list of some of the potential benefits and risks to BZD use and tapering when considering whether to taper the medication. Clinicians should consider the likelihood of each benefit and risk for the individual patient. The narrative notes risk/hazard ratios available in the published literature

*Including compassionate use for end of life or palliative care

^†^Including risks associated with counterfeit BZDs from the illicit drug market, such as contamination with HPSOs (e.g., fentanyl) and novel synthetic substances

**Table 3:** Risk of Clinically Significant BZD Withdrawal

Duration of BZD use	Frequency of BZD use	Total daily BZD dose	Risk of clinically significant withdrawal*
Any	≤ 3 days per week	Any	No
< 1 month	≥ 4 days per week	Any	Lower risk, but possible
1–3 months	≥ 4 days per week	Low^†^	Lower risk, but possible
1–3 months	≥ 4 days per week	Moderate^‡^ to high^§^	Yes, with greater risk with increasing dose and duration
≥ 3 months	≥ 4 days per week	Any	Yes, with greater risk with increasing dose and duration

This table summarizes estimates of risk of experiencing clinically significant withdrawal depending on the dose, duration, and frequency of BZD use. This table is based on clinical consensus of the CGC. It is intended to provide general guidance and should not replace clinical judgment

*Many factors influence the risk of physical dependence and BZD withdrawal syndrome, including but not limited to age, co-occurring physical and mental health conditions, other substance use, and prior history of withdrawal

^†^A low daily dose is estimated as 10 mg diazepam equivalents or less (e.g., ≤ 0.5 mg clonazepam, ≤ 2 mg lorazepam, ≤ 1 mg alprazolam). See Appendix B for BZD dose equivalents

^‡^A moderate daily dose is estimated as 10–15 mg diazepam equivalents (e.g., 0.5–1.5 mg clonazepam, 2–3 mg lorazepam, 1–2 mg alprazolam). See Appendix B for BZD dose equivalents

^§^A high daily dose is estimated as more than 15 mg diazepam equivalents (e.g., > 1.5 mg clonazepam, > 3 mg lorazepam, > 2 mg alprazolam). See Appendix B for BZD dose equivalents

2. Clinicians should avoid abruptly discontinuing BZD medication in patients who are likely to be physically dependent on BZDs and at risk of BZD withdrawal (see Table 3; ***Low Certainty***, Strong Recommendation).
  a Tapering is indicated for patients who are likely to be physically dependent when the risks of BZD medication outweigh the benefits (***Low Certainty***, Strong Recommendation).
  b Clinicians should consider either discontinuation or a short taper for patients who are unlikely to be physically dependent when the risks of BZD medication outweigh the benefits (*Clinical Consensus*, Strong Recommendation).

3. If the BZD medication is discontinued without a taper in patients who are unlikely to be physically dependent, clinicians should counsel patients to report the emergence of withdrawal and/or rebound symptoms (*Clinical Consensus*, Strong Recommendation).
  a If significant symptoms emerge, clinicians can consider using medications for symptom management or restarting the BZD medication and initiating a taper (*Clinical Consensus*, Conditional Recommendation).

**Recommendation for Partnering with Patients**

4. Clinicians should develop the BZD tapering strategy in coordination with patients and their care partners in a shared decision-making process whenever possible (*Clinical Consensus*, Strong Recommendation).

**Recommendation for Level of Care Considerations**

5. BZD tapering can typically be managed in outpatient settings. However, clinicians should consider inpatient care for BZD tapering when:
  a Patient presentation indicates an imminent risk of significant harm related to continued use of the BZD medication (e.g., medication interaction, overdose, accidents, falls, suicidality or other self-harm) that is unlikely to be rapidly mitigated by the initial dose reduction of the BZD taper (*Clinical Consensus*, Strong Recommendation)
  b Patient symptoms and/or co-occurring physical or mental health conditions are anticipated to complicate BZD tapering in a way that cannot be safely managed in an outpatient setting (*Clinical Consensus*, Strong Recommendation)
  c The patient is experiencing or imminently anticipated to experience severe or complicated BZD withdrawal (*Clinical Consensus*, Strong Recommendation)

**Recommendations for the Tapering Process**

6. Clinicians should generally consider dose reductions of 5 to 10% when determining the initial pace of the BZD taper. The pace of the taper should typically not exceed 25% every 2 weeks (*Clinical Consensus*, Strong Recommendation).
7. Clinicians can consider transitioning patients without contraindications to a comparable dose of a longer-acting BZD medication for the taper (*Clinical Consensus*, Conditional Recommendation).
8. Clinicians should tailor tapering strategies to each individual patient and adjust the taper based on a patient’s response (*Clinical Consensus*, Strong Recommendation).
9. Clinicians should evaluate patients undergoing tapering for signs and symptoms related to the BZD taper with each dose reduction (*Clinical Consensus*, Strong Recommendation).

**Recommendations for Adjunctive Interventions**

10. Clinicians should offer patients undergoing BZD tapering behavioral interventions tailored to their underlying conditions (e.g., cognitive behavioral therapy (CBT), cognitive behavioral therapy for insomnia (CBT-I)) or provide them with referrals to access these interventions (***Low Certainty***, Strong Recommendation).
11. Clinicians should first consider pausing or slowing the pace of the BZD taper when patients experience symptoms that significantly interfere with the taper (e.g., sleep difficulty, anxiety). However, clinicians can also consider use of adjunctive medications (*Clinical Consensus*, Conditional Recommendation).

**Recommendations for Management of Severe or Complicated Withdrawal Symptoms**

12. Clinicians should manage patients experiencing severe or complicated withdrawal in inpatient or residential medically managed settings (e.g., residential withdrawal management program) with:

- Monitoring for signs and symptoms of BZD withdrawal, including regularly measuring vital signs and using structured assessment tools (*Clinical Consensus*, Strong Recommendation)
- Assessments for seizure risk, managed as appropriate (*Clinical Consensus*, Strong Recommendation)

13. Tapering with very long-acting agents such as phenobarbital:
  a Can be considered for BZD withdrawal management in inpatient settings (***Low Certainty***, Strong Recommendation).
  b Should only be conducted by or in consultation with clinicians experienced in the use of these agents for the purpose of BZD withdrawal management (*Clinical Consensus*, Strong Recommendation).

14. Clinicians should avoid rapid BZD reversal agents such as flumazenil for the purpose of BZD tapering due to risks for refractory seizure, cardiac dysrhythmias, and other adverse effects (*Clinical Consensus*, Strong Recommendation).
15. Clinicians should avoid general anesthetics such as propofol or ketamine for the purpose of BZD tapering (*Clinical Consensus*, Conditional Recommendation).

**Recommendations for Patients Co-prescribed Benzodiazepines and Opioids**

16. Because patients co-prescribed BZDs and opioids are at increased risk of respiratory depression, clinicians should assess the risks and benefits of continued BZD prescribing at least every 3 months or with every related clinical encounter or prescription renewal, whichever is more frequent (*Clinical Consensus*, Strong Recommendation).
17. Clinicians should offer to provide or prescribe opioid overdose reversal medication (e.g., naloxone) for all patients co-prescribed BZDs and opioids (*Clinical Consensus*, Strong Recommendation).
18. Clinicians should consider additional strategies for mitigating risk, including using the lowest effective doses of BZD and opioid medications and optimizing non-opioid interventions (*Clinical Consensus*, Strong Recommendation).

**Recommendations for Patients with Benzodiazepine and Other Substance Use Disorders**

19. Clinicians should consider more frequent assessments of the risks and benefits of continued BZD prescribing for patients with co-occurring SUDs and/or other co-occurring addictions (e.g., behavioral addictions) compared with the general guidance in Recommendation 1 (*Clinical Consensus*, Strong Recommendation).
20. When tapering BZD medication in patients with SUD, clinicians should manage the underlying SUD concurrently with the BZD taper (*Clinical Consensus*, Strong Recommendation).
21. Clinicians should not use BZD prescribing or tapering considerations as a reason to discontinue or disrupt a patient’s medications for SUD treatment, including buprenorphine and methadone (*Clinical Consensus*, Strong Recommendation).
22. Following the taper, clinicians should continue to monitor and treat any underlying SUDs or refer patients to an appropriate level of care for continuing care (*Clinical Consensus*, Strong Recommendation).
23. Clinicians should offer patients harm reduction services or provide them with referrals to access these services (*Clinical Consensus*, Strong Recommendation).
  a Clinicians should offer to provide or prescribe opioid overdose reversal medication (e.g., naloxone) and provide or refer patients for related education (*Clinical Consensus*, Strong Recommendation).
  b Clinicians can consider providing or referring patients to community services for drug checking or other safe use supplies (e.g., fentanyl test strips, xylazine test strips, sterile syringes) and related education (*Clinical Consensus*, Conditional Recommendation).

**Recommendations for Patients with Co-occurring Psychiatric Disorders**

24. Clinicians should optimize evidence-based treatment for any psychiatric disorder prior to the BZD taper or concurrently if clinically indicated (Clinical Consensus, Strong Recommendation).
25. Clinicians should strongly consider tapering BZD medication in patients with post-traumatic stress disorder (PTSD; *Clinical Consensus*, Strong Recommendation).
26. Clinicians should monitor sleep closely during BZD tapering in patients with mood or psychotic disorders, particularly for patients with bipolar disorder as sleep disturbance can trigger episodes of mania (*Clinical Consensus*, Strong Recommendation).
  a If patients with a mood and/or psychotic disorder experience significant sleep disturbance, clinicians should pause the taper until the symptoms resolve due to the risk of destabilization (*Clinical Consensus*, Strong Recommendation).

**Recommendation for Older Adults**

27. Clinicians should generally taper BZD medication in older adults unless there are compelling reasons for continuation (*Clinical Consensus*, Strong Recommendation).

**Recommendations for Patients Who Are Pregnant or Lactating**

28. Clinicians should weigh the risks and benefits for the maternal–fetal dyad when considering continued BZD prescribing or tapering for pregnant patients (*Clinical Consensus*, Strong Recommendation).
29. For infants who have been exposed to BZD in utero and are at risk of neonatal withdrawal, clinicians should:
  a Encourage breastfeeding, which can reduce neonatal withdrawal symptoms (*Clinical Consensus*, Strong Recommendation)
  b Communicate with the infant’s healthcare provider (with parental consent) regarding exposure to BZDs (*Clinical Consensus*, Strong Recommendation)

## PATIENT ENGAGEMENT AND SHARED DECISION-MAKING

**Figure 1:**
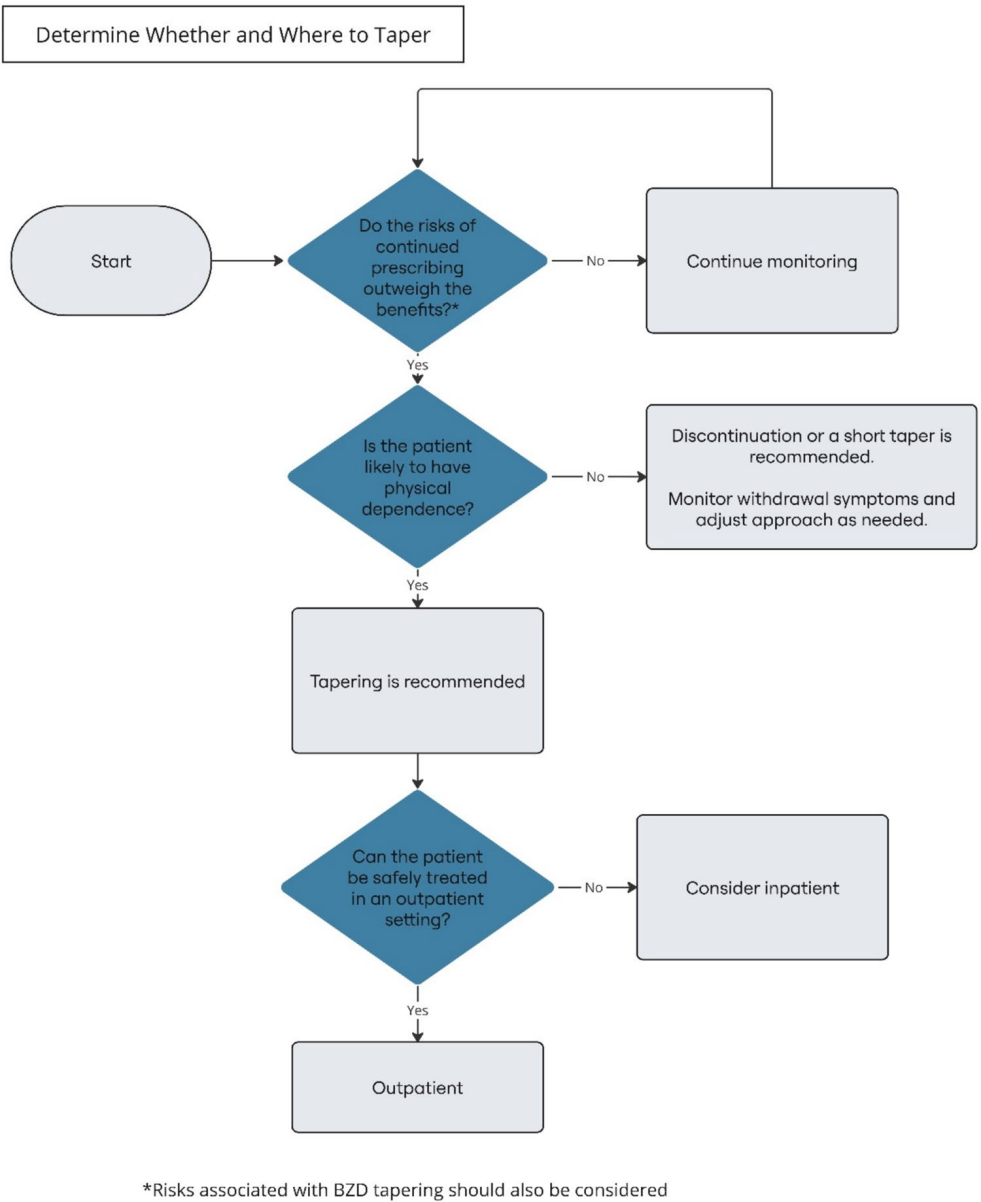
Determining whether and where to taper

BZD tapering can be a challenging process for both clinicians and patients. Decisions relating to whether, when, and how to taper are often complex and need to consider a variety of factors, including each patient’s needs, preferences, and concerns (Fig. 1). Many patients will be anxious about tapering a medication they believe to be beneficial. They may be understandably afraid of the potential for withdrawal symptoms or recurrence of physical or mental health symptoms.

Patient education is critical during the BZD tapering process. Many patients may interpret withdrawal symptoms as an indication that they need the medication to manage their condition(s). Collaborative relationships with patients and their care partners with clear communication about what to expect throughout the process can make a significant difference in a patient’s experience and outcomes. The CGC emphasized the importance of patient engagement and shared decision-making, highlighting these considerations throughout the Guideline (see Box 4).

**Box 4:** Shared Decision-Making

The recommendations in this Guideline should be interpreted in the context of shared decision-making with patients. In other words, when a recommendation says, “clinicians should consider,” it should be understood to include “in partnership with the patient.”	

## CONSIDERATIONS FOR TAPERING BENZODIAZEPINES

### Determining Whether to Taper

**Recommendations for Considerations for Tapering Benzodiazepines**

1. Clinicians should ideally assess the risks and benefits of ongoing BZD prescribing at least every 3 months for each patient taking BZD medications (see Tables 2 and 3; *Clinical Consensus*, Strong Recommendation).
  a At a minimum, clinicians should assess the risks and benefits with each new BZD prescription or BZD prescription renewal (Clinical Consensus, Strong Recommendation).
  b Clinicians should review the information in the relevant prescription drug monitoring programs (PDMPs) as part of the risk–benefit assessment (*Clinical Consensus*, Strong Recommendation).
2. Clinicians should consider discontinuation or a short taper for patients who are unlikely to be physically dependent when the risks of BZD medication outweigh the benefits (*Clinical Consensus*, Strong Recommendation).
  - Tapering is indicated for patients who are likely to be physically dependent when the risks of BZD medication outweigh the benefits (***Low Certainty***, Strong Recommendation).
  - Clinicians should consider discontinuation or a short taper for patients who are unlikely to be physically dependent when the risks of BZD medication outweigh the benefits (*Clinical Consensus*, Strong Recommendation).
3. If the BZD medication is discontinued without a taper in patients who are unlikely to be physically dependent, clinicians should counsel patients to report the emergence of withdrawal and/or rebound symptoms (*Clinical Consensus*, Strong Recommendation).
  a If significant symptoms emerge, clinicians can consider using medications for symptom management or restarting the BZD medication and initiating a taper (*Clinical Consensus*, Conditional Recommendation).

**Implementation Considerations**

- When considering the risks and benefits of continued BZD prescribing, clinicians should screen patients for non-prescription use of BZDs and use of other substances that may interact with BZDs or impact the tapering process.
- When the risks of BZD medication outweigh the benefits, clinicians should initiate a conversation with patients about tapering (or discontinuation if patients are unlikely to be physically dependent). Clinicians should elicit information from patients about their expectations and concerns about the tapering process and discuss them. Clinicians should discuss alternatives for managing the underlying condition(s) for which the BZD was initially prescribed that may be more effective and carry less risk compared to BZDs (e.g., selective serotonin reuptake inhibitors (SSRIs) and/or CBT for anxiety disorders, CBT-I for primary insomnia).
- The goal of tapering may be discontinuing the BZD medication or reducing the BZD dose to the lowest effective dose where the risks no longer outweigh the benefits. Clinicians should reassess risks and benefits throughout the tapering process to inform decision-making.
- Although many patients who have been taking BZDs for a short period of time (e.g., less than a month) are able to discontinue the medication without a taper, clinicians can consider a short taper. A short taper may be indicated if clinicians have concerns for clinically significant withdrawal (e.g., due to the pharmacological properties of the BZD, patient age, comorbidities, other substance use, prior history of withdrawal) or patients express concerns about discontinuing the medication.
- Many healthcare systems may not be able to manage the volume of patients who would benefit from BZD tapering. As such, clinicians and healthcare systems may need to triage patients, prioritizing those at higher risk of harm related to continued BZD use. See “Implementing this Guideline” for further discussion.

#### Rationale

**Figure 2:**
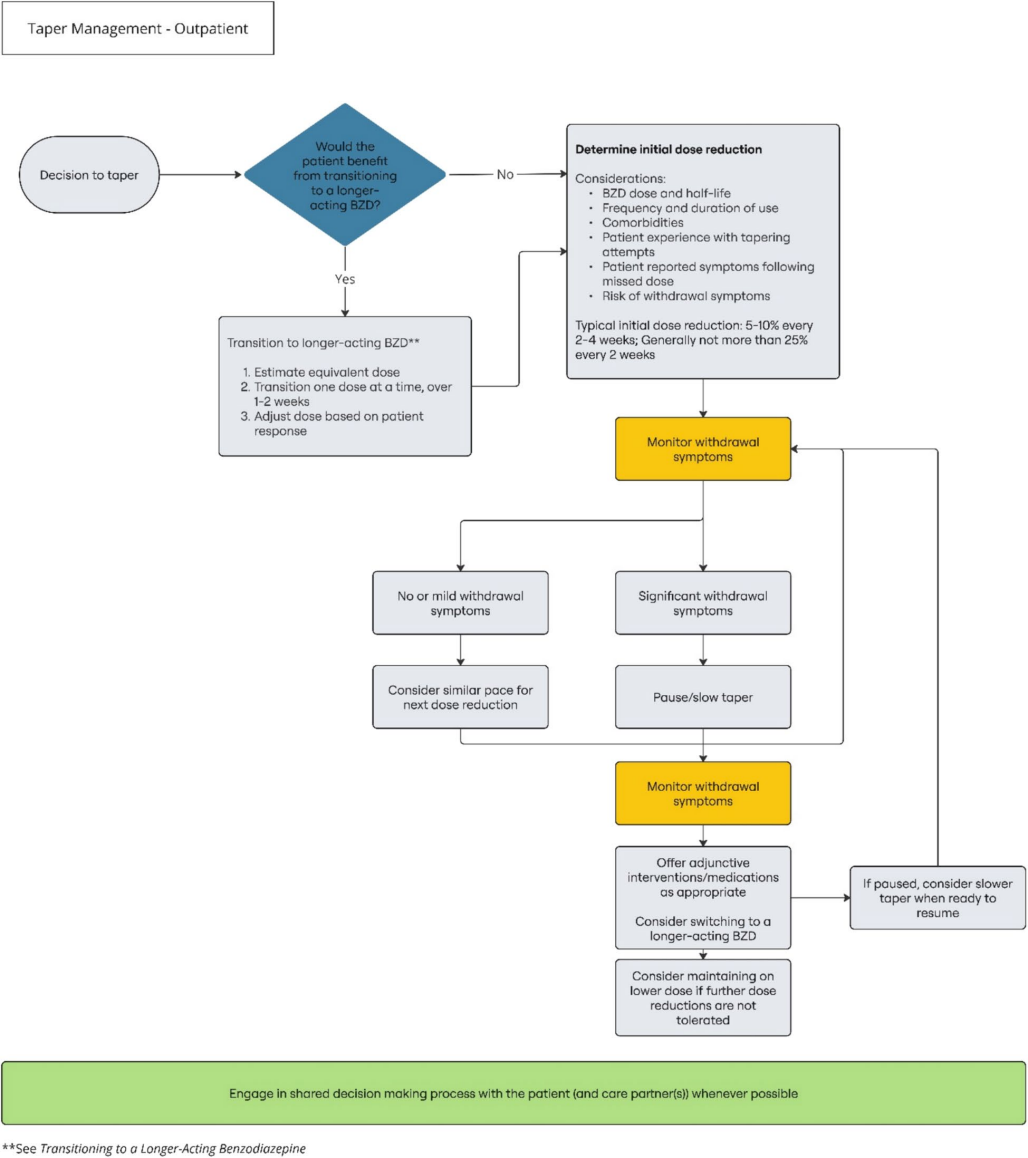
Outpatient taper management

In 2020, the FDA updated the required Boxed Warning for BZD medications to describe the risks of physical dependence, withdrawal, and BZD use disorder.^4^ The associated Drug Safety Communication encouraged prescribers to carefully weigh the risks and benefits of BZD medication, limit the dose and duration to what is needed to achieve the clinical goal, and monitor patients for BZD misuse and BZD use disorder. When prescribing BZDs, clinicians should have a thoughtful strategy for medication management that regularly reassesses the risks and benefits of continued prescribing, as well as a patient-centered plan for tapering the medication when the benefits no longer outweigh the risks (Fig. 2).

The risks of BZD use evolve as a patient continues to take the medication. Although most patients taking BZDs as prescribed will not develop BZD use disorder, the risk of physical dependence and BZD use disorder increases with time, particularly in patients who use alcohol or other substances.^31^ Long-term BZD use is often associated with more risks than benefits. Significant risks include oversedation and other sleep disturbances, cognitive impairment, falls, motor vehicle accidents, and fatal and nonfatal overdose.^3^ Despite these known risks, clinicians often encounter patients who have been taking prescribed BZD for years.

Short-term BZD use is associated with decreased anxiety and insomnia, with duration of use typically recommended to not exceed 4 weeks.^32,33^ This guidance reflects the general lack of evidence for the long-term clinical benefit of BZDs, as well as research demonstrating that the clinical benefits may decrease over time while the risks persist.^32–34^ Meta-analyses of patients taking BZDs for insomnia demonstrated minor improvements in sleep onset, increased sleep duration, and decreased nighttime awakenings.^35,36^ However, therapeutic effects diminished within days to weeks due to changes in BZD receptor density and/or affinity resulting from chronic use, while risks remain present. A meta-analysis of randomized controlled trials (RCTs) comparing BZDs to placebo for insomnia in adults over age 60 showed a 3.8-fold increase in daytime sedation, a 4.8-fold increase in cognitive impairment, and a 2.6 fold increase in incidence of psychomotor effects (e.g., falls, motor vehicle accidents).^35^ Another meta-analysis showed an increased risk of hip fractures (RR 1.34) associated with current and recent BZD use in older adults.^37^ In addition to its psychomotor effects, BZDs may increase the risk of orthostatic hypotension in older adults, which also contributes to fall risks.^38^

Although long-term BZD use should generally be avoided, exceptions do exist. For example, long-term BZD use may be indicated in patients with severe treatment-resistant generalized anxiety disorder (GAD) or bipolar spectrum disorders.^39–41^ Long-term BZD use may also be appropriate for medical conditions such as complex seizure disorders,^42^ spasticity,^43,44^ sleep disorders involving abnormal movements (e.g., rapid eye movement (REM) sleep behavior disorder, restless leg syndrome),^45^ and catatonia.^46,47^ Finally, BZDs have a role in palliative and end-of-life care.^48^ In any of these patient populations, clinicians should consider consulting with an appropriate specialist to determine whether BZD tapering is indicated and manage the process when it is.

#### Risk and Benefit Considerations

Many factors are relevant when determining whether it is in a given patient’s best interest to taper or discontinue BZDs (see Table 2). Clinicians should first consider the risks and benefits of continued BZD use. If the risks outweigh the benefits, clinicians should then consider the potential risks associated with tapering. Risks and benefits exist along a continuum. There is no precise formula for determining whether the risks outweigh the benefits for a given patient at a given time. Clinicians use their judgment—in consultation with the patient, their care partners, and other members of the treatment team—when considering which course of action is in a patient’s best interest. This decision may be more challenging when the risks and benefits are closely balanced; in these cases, clinicians should consider whether continuing or tapering the BZD is likely to positively impact what matters most to patients.^49^ When determining the balance of risks and benefits, clinicians should consider the following:

- How significant are the potential benefits?
  - Could alternative interventions achieve similar benefits?
- How significant are the potential risks?
  - How imminent are the risks?
  - How effectively can the risks be managed?

Risks associated with BZD use evolve dynamically with age and in response to changes in a patient’s health and lifestyle. Age-related changes in pharmacokinetics and pharmacodynamics are well known and can increase the risk of adverse effects from BZDs.^50^ In addition, changes in patients’ use of nicotine/tobacco products can influence metabolism of BZDs.^51^ Furthermore, new health conditions and the medications used to treat them can also influence patients’ risks.

Risk–benefit assessments should include regular screening for signs of BZD misuse and use disorder. Validated screening tools for substance use and prescription drug misuse can be found in Appendix 7. Clinicians should consider how a patient’s substance use impacts their risks related to the prescribed BZD medication. If patients exhibit signs of potential BZD misuse, such as frequently requesting early refills, increased dosage, or number of pills, clinicians should assess the possibility of BZD misuse, related risks, and if tapering is indicated. Drug testing may help inform this assessment and can help differentiate between medication misuse and diversion (see “Drug Testing”). Patients who are misusing medications should be assessed or referred for further assessment and treatment for potential BZD use disorder.

#### Medication Review

Given that polypharmacy is common among patients who take prescribed BZDs, clinicians should conduct a thorough medication review as part of regular risk–benefit assessments as well as prior to beginning a taper.^19^ PDMPs can help detect multiple BZD prescriptions, concurrent prescriptions of other controlled substances with CNS depressant effects, and other issues related to polypharmacy. Although mandates regarding PDMP use vary widely across states, the CGC noted that prescribing clinicians should review the relevant PDMP as part of the risk–benefit assessment at the time of each new BZD prescription and renewal. In addition, most electronic health record (EHR) systems have access to external prescribing databases that include noncontrolled medications prescribed to patients that may interact with BZDs.

BZD medication interactions include additive sedation with sedating medications (e.g., antihistamines, antipsychotics, opioids, gabapentinoids) and pharmacokinetic interactions with cytochrome P450 (CYP) enzymes (see Appendix 1). Combined use of BZDs and opioids increases the risk of adverse events, including fatal and nonfatal overdose, due to the CNS depression caused by both medication classes (see “Patients Co-prescribed Benzodiazepines and Opioids” for further discussion).^7,23,52^ Excessive sedation has been observed when BZDs have been used with CYP3 A4 inhibitors, such as antibiotics like clarithromycin and erythromycin.^53^ Additionally, clinicians should explore patients’ use of nonprescribed opioids and sedatives, as well as their consumption of alcohol (a CNS depressant) and grapefruit juice (a strong CYP3 A4 inhibitor).^53^

#### Patient Engagement

BZD prescription renewals represent opportunities for clinicians to proactively review the risks and benefits of BZD use with patients and educate them on the importance of limiting the duration of BZD use. Many patients and clinicians are unaware the clinical benefits of BZDs can decrease within a few weeks, while risks continue (e.g., falls) or increase (e.g., physical dependence).

Because of the risks associated with regular BZD use, the CGC recommends that clinicians assess the risks and benefits of continued BZD prescribing with each new prescription and prescription renewal. Virtual or telephonic follow-up visits can be leveraged for this purpose. Clinicians should conduct risk–benefit assessments for patients with newly initiated BZD prescriptions within 1 month of writing the script or sooner, given how quickly BZD dependence can develop.^4^ Clinicians should discuss any adverse effects of BZD use (including those discussed in Table 2) and elicit patients’ perceptions on the risks and benefits of ongoing use. Going forward, clinicians should reassess the risks and benefits of continued BZD every 3 months, at minimum. Clinicians should be mindful of the many types of bias that may exist when making decisions regarding initiating a taper (see “Health Disparities”).^54^

#### Consideration of Risks Associated with Tapering

Even when the risk–benefit assessment favors BZD tapering, discontinuation of the medication may present risks.^55^ A recent retrospective cohort study of a US commercial healthcare claims database (*n* = 353,576 patients) by Maust et al.^55^ indicated that the mortality risk among patients who discontinued BZD use over a 6-month period was 1.6 times higher compared to patients who continued use. However, the analysis could not examine the reason for discontinuation and did not account for the rate of tapering or discontinuation, factors that will be important to consider to fully interpret the finding of increased mortality risk.^55^ The association identified in the study between discontinuation of BZD and mortality may be related to the underlying reason for BZD discontinuation such as declining health (e.g., liver or kidney dysfunction), falls, or cognitive decline—rather than having been caused by the discontinuation. In contrast, an RCT by Tannenbaum et al.^56^ evaluating a patient educational intervention for BZD tapering did not observe any major adverse events in 303 patients, while an RCT by Vicens et al.^57^ only reported one adverse event among 359 patients after initiating a primary care–based intervention for BZD tapering.

The CGC carefully considered the results of the Maust et al.^55^ study on mortality risk and do not believe these findings should outweigh the extensive body of literature characterizing the risks associated with BZD use, especially since the reason for discontinuation and the speed of the taper were not considered in the analysis.^55^ However, as discussed throughout this Guideline, prescribing clinicians should carefully consider the risks and benefits of continued BZD use as well as the likely risks and benefits of tapering for a given patient and should not assume tapering is the right choice for all patients. Some patients may have risks associated with discontinuing the BZD prescription (see Table 2) that clinicians should take into account based on an individual patient’s needs and circumstances. Tapering should be undertaken carefully, with close monitoring and adjustments based on a patient’s response. More research is needed to better understand the potential risks of BZD tapering and develop strategies to mitigate them.

#### Tapering Versus Discontinuation

If the clinician determines, in the context of the risk/benefit assessment and shared decision-making, continuing the BZD prescription is no longer appropriate, they need to first determine if patients are likely to be physically dependent on the medication, and therefore at risk of withdrawal. The risk of severe withdrawal syndrome following regular use of therapeutic doses of BZD has been recognized since the 1960 s.^58^ Factors including use of shorter acting BZD, higher dose, and longer duration of treatment contribute to a higher likelihood of physical dependence and risk of severe withdrawal.^59^

If patients are at risk of withdrawal, the medication should be tapered rather than abruptly discontinued. While limited evidence was found comparing tapering strategies, the systematic review identified two RCTs with 70 participants that compared a BZD taper to abrupt cessation.^60,61^ Both RCTs had an unclear risk of bias. Although labeled “gradual,” the taper duration was only 7–8 days. There was no difference in the rate of complete BZD discontinuation, return to BZD use after a period of discontinuation, delirium, or study completion between groups. However, patients in the tapering group reported significantly less severe BZD withdrawal and insomnia symptoms after 4 days, and up to 4 weeks, compared to patients who abruptly stopped their BZD use. In discussing these studies, the CGC agreed that benefits of tapering are likely to outweigh the risks of abrupt discontinuation, especially when using more gradual tapering strategies. See Table 1 in Supplementary Material 5 for the full Evidence to Decision Table comprising these studies.

Although many patients who have been taking BZDs for a short period of time (e.g., less than a month) are able to discontinue the medication without a taper, some will experience significant withdrawal symptoms. Similarly, some patients who have been taking BZDs at a low dose for an intermediate amount of time (e.g., 6 weeks) may not be physically dependent. Defining strict thresholds for the risk of physical dependence and withdrawal is difficult because many factors beyond the dose and duration of BZD use impact risk, including age, co-occurring physical and mental health conditions, the pharmacological properties of the given BZD, other medication and substance use, and prior history of withdrawal, among others.

Table 3 summarizes the risk of withdrawal by dose, duration, and frequency of BZD use. While no direct evidence was found for predicting risk of withdrawal, the CGC agreed that these factors were most salient in the determination. Clinicians should consider this information in the context of each patient’s presentation when determining if patients are likely to be physically dependent and tapering is indicated. It should be noted that alprazolam—which is unique in having a very short half-life, rapid onset of action, and no active metabolites—tends to be associated with a more rapid onset of physical dependence.^62^ Therefore, a taper may be appropriate for patients taking this medication daily, even for a short duration (e.g., 2–4 weeks).

If physical dependence is difficult to determine, clinicians should elicit information from patients regarding any concerns about discontinuation or preferences for tapering. Clinicians should gather information about each patient’s likelihood for physical dependence and risk of withdrawal, including asking if they have experienced withdrawal symptoms following missed doses in the past. Clinicians should also ask patients about any past experiences with withdrawal symptoms associated with tapering or discontinuing BZDs, especially adverse events such as seizures. Determining use of alcohol is also important, particularly if patients engage in ongoing daily alcohol use or have experienced severe withdrawal from alcohol or other substances in the past; slower and/or alternate tapering strategies may be indicated in these situations. If SUD may be present, clinicians should consider consulting addiction specialists, when possible.

If physical dependence is unlikely and the decision is made to discontinue the BZD without a taper, clinicians should educate patients about potential withdrawal and/or rebound symptoms that may occur and encourage patients to report these symptoms. If patients report significant symptoms, clinicians can consider initiating a taper.

### Partnering with Patients

**Recommendation for Partnering with Patients**

4. Clinicians should develop the BZD tapering strategy in coordination with patients and their care partners in a shared decision-making process, whenever possible (*Clinical Consensus*, Strong Recommendation).

**Implementation Considerations**

- Clinicians can consider utilizing educational resources when developing BZD tapering strategies with patients.
- Clinicians can consider utilizing a motivational interviewing (MI) framework, which is patient-centered and seeks to involve patients in resolving ambivalence to change.

#### Rationale

Evidence supports the importance of shared decision-making across multiple clinical settings.^63,64^ One systematic review of 39 studies of shared decision-making and patient outcomes demonstrated that patient-perceived shared decision-making was associated with improved positive affective/cognitive outcomes such as understanding, satisfaction, and trust.^64^ When BZD tapering is indicated, clinicians should initiate a conversation with patients with a goal of shared decision-making. Clinicians should invite patients to share their perceptions about the benefits and risks of continuing BZDs, as well as share their own with patients. Although some patients will be understandably reluctant to consider tapering a medication they have been taking for a long period of time and consider helpful, others may welcome the opportunity to minimize potential adverse effects and explore more optimal ways of managing their underlying conditions.^65,66^ Appendix 7 includes resources on the management of conditions for which BZDs are commonly prescribed, including insomnia, anxiety, seizure disorders, and chronic pain, among others.

Clinicians often do not discuss tapering with patients and continue renewing prescriptions because of concern for withdrawal, as well as patients’ perception of benefits.^65^ Clinicians may feel uncomfortable starting these conversations due to the perceived sensitivity and difficulty of the topic. However, many patients indicate they would be open to considering tapering chronic medications, including BZDs, if clinicians discussed it with them.^66,67^

In addition to discussing the risks and benefits of BZD use, clinicians should acknowledge and discuss the risks and benefits associated with BZD tapering or discontinuation. Patients can experience life-threatening withdrawal symptoms with abrupt or rapid discontinuation of BZDs, and some patients experience significant symptoms even with gradual dose reduction.^2,20,68^ As such, it is important for clinicians to adopt a patient-centered approach when considering BZD tapering, acknowledging patient concerns and engaging in a shared decision-making process.^33,69^ Engaging patients in discussions about their BZD use serves two important purposes:

1. Clinicians are presented with opportunities to educate patients on the risks and benefits of BZD use, alternative pharmacological and nonpharmacological treatment options to manage the condition for which they are taking BZDs, and the tapering process. Discussions on tapering should prepare patients for what they can expect during the process, including potential withdrawal symptoms and how they will be managed.
2. Patients are presented with opportunities to help clinicians understand how their BZD use impacts them, as well as their treatment goals and preferences. This insight into each patient’s experience with BZDs can help inform clinicians’ education efforts for a given individual. These discussions can empower patients to be active participants in their health care by sharing valuable information to help their clinicians better tailor treatment plans, including BZD tapering strategies, to incorporate their unique needs, goals, and preferences.

Education is a vital component of conversations about tapering. Clinicians should inform patients on how the clinical benefits of BZDs can decrease over time while the risk of adverse effects increases (e.g., physical dependence) or persists (e.g., falls, motor vehicle accidents). Clinicians may stress the benefits patients can expect from reducing or discontinuing their BZD medication, such as improved cognition and psychomotor functioning.^70^ They should acknowledge the reality of physical dependence associated with BZD use, as well as potential withdrawal and/or rebound symptoms that may arise during tapering. Clinicians should expect and inform patients that fully tapering off their BZD medication may take months to years, particularly if patients have been on a high dose for an extended period of time.

Clinicians can utilize educational resources—such as those available from the Eliminating Medications through Patient Ownership of End Results (EMPOWER)—when developing tapering strategies with patients (see Appendix 7).^71^ MI techniques may help build patient buy-in and formulate a joint tapering strategy. Patients should be reassured that they will be supported throughout the tapering process. These conversations should be conducted in a patient’s preferred language and at a level commensurate with their medical literacy.

The concept of shared decision-making is built on engaging patients as active participants in their treatment rather than as passive recipients.^72^ It also highlights the value of each patient’s own experiences, thereby promoting their autonomy and empowering them to improve their health.^72^

### Level of Care Considerations

**Recommendation for Level of Care Considerations**

5. Although BZD tapering can typically be managed in outpatient settings, clinicians should consider inpatient care for BZD tapering when:

- Patient presentation indicates an imminent risk of significant harm related to continued use of the BZD medication (e.g., medication interaction, overdose, accidents, falls, suicidality or other self-harm) that is unlikely to be rapidly mitigated by the initial dose reduction of the BZD taper (*Clinical Consensus*, Strong Recommendation)
- Patient symptoms and/or co-occurring physical or mental health conditions are anticipated to complicate BZD tapering in a way that cannot be safely managed in an outpatient setting (*Clinical Consensus*, Strong Recommendation)
- The patient is experiencing or imminently anticipated to experience severe or complicated BZD withdrawal (*Clinical Consensus*, Strong Recommendation)

**Implementation Considerations**

- Clinicians should use the risk–benefit assessment to inform decisions regarding level of care.
- Most patients who have developed physical dependence to BZDs can complete tapering in outpatient settings. Clinicians should consider inpatient tapering when imminent risks to patient safety cannot be mitigated rapidly with outpatient treatment.
- Clinicians should consider prior history of severe or complicated BZD or alcohol withdrawal when determining patients’ current withdrawal risk.
- If the clinician cannot obtain authorization for inpatient care, they should consider whether attempting an outpatient taper or continuing the medication poses a greater risk.

#### Rationale

BZD tapering can typically be accomplished in outpatient settings. Although no direct evidence was found regarding level of care decisions for BZD tapering, the CGC agreed that most patients can undergo tapering in outpatient settings. In our systematic review, 42 of the 57 studies included were conducted in outpatient settings. This section of the Guideline details situations where additional support may be required to accomplish BZD tapering. The level of care options for many patients may be limited to outpatient or inpatient settings or, in rare instances, skilled nursing facilities.

Patients with suspected or confirmed SUD or other psychiatric disorders may require additional support, especially if they have had previous unsuccessful attempts to taper from BZDs. These patients may be eligible for a broader range of services, including intensive outpatient treatment, partial hospitalization programs, and residential care within the specialty addiction and mental health treatment systems. Specific considerations for these patients are discussed in “Patients with Benzodiazepine and Other Substance Use Disorders” and “Patients with Co-occurring Psychiatric Disorders.”

Clinicians should consider inpatient settings if patient presentation indicates an immediate risk of serious harm related to continued BZD use. For example, patients who have experienced recent falls, motor vehicle accidents, or overdose related to BZD use or are exhibiting acute suicidality or self-harm behaviors are potential candidates for inpatient management and stabilization if a significant risk of serious harm is unlikely to be mitigated rapidly by the initial dose reduction of the BZD taper or other interventions that can be provided in outpatient settings.

Clinicians should consider inpatient care for patients who have significant and unstable co-occurring physical or mental health conditions (e.g., under-controlled or uncontrolled seizure disorder) that cannot be managed safely in outpatient settings. Additionally, inpatient care is generally indicated if patients are experiencing or anticipated to experience severe or complicated withdrawal. Although withdrawal risk is difficult to predict, a history of complicated withdrawal involving seizure or delirium is a significant predictor of future complications and should be considered when assessing current risk. Patients who have a history of moderate to severe alcohol withdrawal may be more likely to experience more severe BZD withdrawal symptoms due to the cross-tolerance of alcohol and BZDs (see “Assessing the Potential for Physical Dependence and Withdrawal”).

In certain situations, patients may desire a more rapid taper. The CGC noted that individual circumstances (e.g., work requirements, child custody issues) may motivate patients to discontinue BZD use relatively rapidly. Assuming medical necessity can be established, these patients may be candidates for inpatient tapering.

It is important to note that the tapering process may take place in more than one setting. For example, patients who have significant risk factors may begin BZD tapering in inpatient settings and transition to outpatient settings for continued management once they are stable and able to tolerate the ongoing tapering process (see “Management of Severe or Complicated Withdrawal Symptoms”).

Considerations related to the potential impact of treatment in inpatient settings for a given patient are also important. For example, hospital admission can trigger distress, confusion, and delirium and lead to worse outcomes in patients with dementia or other neurological issues.^73,74^ These risks should be considered when determining the most appropriate treatment setting.

## BENZODIAZEPINE TAPERING STRATEGIES

### The Tapering Process

**Recommendations for the Tapering Process**

6. Clinicians should generally consider dose reductions of 5 to 10% when determining the initial pace of the BZD taper. The pace of the taper should typically not exceed 25% every 2 weeks (*Clinical Consensus*, Strong Recommendation).
7. Clinicians can consider transitioning patients without contraindications to a comparable dose of a longer-acting BZD medication for the taper (*Clinical Consensus*, Conditional Recommendation).
8. Clinicians should tailor tapering strategies to each individual patient and adjust the taper based on a patient’s response (*Clinical Consensus*, Strong Recommendation).
9. Clinicians should evaluate patients undergoing tapering for signs and symptoms related to the BZD taper with each dose reduction (*Clinical Consensus*, Strong Recommendation).

**Implementation Considerations**

- Prior to beginning a taper, clinicians should conduct a thorough medication and health review, with particular attention to other psychoactive medications and conditions that may be impacted during the taper.
- Clinicians should design the overall tapering strategy to minimize harms, considering the risk of harm related to continued BZD use and the risk of harms related to tapering the BZD (e.g., withdrawal symptoms, recurrence of symptoms for which the BZD was originally prescribed).
- Clinicians should consider the lower end of the dose reduction range (i.e., 5%) for the first reduction to assess a patient’s initial response, unless there are imminent safety concerns.
- For patients who are likely to have strong physical dependence (e.g., those who have been taking a high dose for more than a year), clinicians should consider a slower taper.
  - For the first reduction, consider the lower end of the dose reduction range (e.g., 5%).
  - For further reductions, clinicians should adjust based on patients’ initial response, considering reduction of 5 to 10% every 6–8 weeks, or slower as appropriate.
- Clinicians can consider the higher end of the dose reduction range (i.e., 10–25%) for patients who are unlikely to have significant physical dependence (i.e., patients who have been taking a lower dose of BZD for a shorter period of time [e.g., less than 3 months]) but for whom tapering is indicated.
- When developing tapering strategies, clinicians should consider patients’ current BZD dose and half-life, frequency and duration of BZD use, co-occurring physical and mental health conditions, and responses to previous missed doses and any prior BZD tapering attempts. Clinicians should also consider patient concerns and anxiety around tapering.
- When patients are taking multiple doses of BZDs each day, clinicians should carefully consider which dose to reduce first. For example, first reducing earlier doses may be appropriate if insomnia is a greater concern, whereas first reducing later doses may be more appropriate if daytime anxiety is a greater concern.
- Tapering strategies, including dosing frequency, should account for the pharmacokinetic properties of the BZD to avoid sharp declines in BZD receptor occupancy.
- Clinicians should monitor patients for symptoms of withdrawal and recurrence with each dose reduction. Virtual or telephonic check-ins can be leveraged for this purpose.
- Clinicians should monitor patients for post-acute signs and symptoms of withdrawal for 2–4 weeks after full discontinuation of the BZD. Clinicians should manage any ongoing symptoms, as appropriate (see “Management of Protracted Withdrawal”), and regularly monitor patients until symptoms are resolved. Telehealth, including audio-only check-ins, may help facilitate monitoring.
- Clinicians should consider pausing or slowing the pace of the taper and/or making smaller dose reductions for patients experiencing significant symptoms related to the BZD taper.
- The BZD tapering process can be more difficult for patients as they approach the point of discontinuation. Clinicians should proactively consider smaller dose reductions and/or slowing the pace of dose reductions as the taper progresses.
- If patients are unable to tolerate further BZD dose reductions, clinicians can consider—in partnership with patients, their care partners, and other members of the care team—maintaining patients on the lower BZD dose with regular risk–benefit assessments consistent with Recommendation 1.
- In some limited instances when patients are experiencing intolerable symptoms, returning to the prior BZD dose and pausing the taper until symptoms stabilize may be appropriate.
- In limited instances when necessary for patient safety, inpatient and medically managed residential settings may use more rapid tapering strategies (see “Tapering with Very Long-Acting Agents”).

#### Rationale

There is significant heterogeneity in patient response to BZD tapering. In the CGC’s experience, some patients who have been taking moderate BZD doses for months experience minimal challenges when tapering at a rate of 10 to 25% every 4 weeks. Other patients—even some who have been taking low BZD doses for a relatively short time (e.g., weeks)—may experience significant withdrawal symptoms, even when tapering at a slower rate. Clinical trials evaluating BZD tapering strategies, which typically have relatively fast (e.g., 25% every 1–2 weeks) and inflexible dose reduction schedules, often have high patient dropout.^75–77^ While no direct evidence was found comparing various tapering strategies, the CGC considered results of these studies, existing guidance, and their own experience in recommending these tapering strategies.

Recommended BZD dose reductions can be achieved in many ways. For example, a goal of reducing the BZD by 20% over 4 weeks could be achieved by any of the following or a combination of the following strategies:

- Reducing the BZD dose by 5% per week
- Reducing the BZD dose by 10% every other week
- Reducing the BZD dose by 20% and maintaining at that lower dose for 4 weeks
- Reducing the number of pills consumed—as an example, clinicians could reduce the number of pills for a 5 mg diazepam twice daily prescription from 60 to 48 for 4 weeks, and patients can decide which pills to skip and when

Smaller, consistent dose reductions may be best for patients who are experiencing significant withdrawal symptoms. However, providing patients with options can help increase patient buy-in and agency in the tapering process.

Although factors that increase the risk of withdrawal are known, no established way to accurately predict which patients may have more difficulty with the taper currently exists. Many patients who have been taking BZDs for less than a month, particularly at low doses, are unlikely to be physically dependent and typically able to discontinue the medication without a taper. However, physical dependence can develop within weeks and is heterogeneous across patients.^4^ As a result, determining whether a patient is at risk of withdrawal is not always clear (see Table 3).^78^ Depending on the specific BZD medication and patient characteristics, some patients who have been taking prescribed BZDs daily or near daily for less than a month may benefit from tapering. One of the most significant challenges the CGC faced in writing this Guideline was developing tapering recommendations that apply broadly in the context of this patient heterogeneity. The recommendations in this Guideline provide flexibility and encourage close patient monitoring to account for the heterogeneity of patient responses.

The CGC noted that patient support is a key factor in the success of a taper, particularly given the heterogeneity in responses to BZD tapering. It is important to educate patients on what to do if they experience concerning symptoms and how to contact their clinicians, if necessary, before the next scheduled visit. This can help patients feel more confident and in control of a process that is often associated with some level of apprehension.

Prior to initiating a BZD taper, clinicians should attempt to coordinate care with a patient’s other BZD prescribers, if applicable, and clinicians managing co-occurring conditions that may be impacted by the taper. In addition, clinicians managing the taper should ideally assume management of all of a patient’s BZD prescriptions. If patients have been taking different BZDs, clinicians should convert and consolidate the medications to an equivalent dose of a single BZD prior to beginning the taper (see Appendix B for BZD dose equivalents). A mutually agreed upon tapering rate between patients and clinicians that avoids a very prolonged taper duration can be an effective strategy for BZD discontinuation.^79^

#### Assessing the Potential for Physical Dependence and Withdrawal

Clinicians should consider the likelihood of a given patient developing withdrawal symptoms during the taper and the anticipated severity of those symptoms (see Table 3). BZD withdrawal symptoms can range from anxiety and sleep problems to seizures and delirium.^2,79–81^ Table 4 provides an overview of common BZD withdrawal signs and symptoms but does not represent an exhaustive list. Distinguishing between withdrawal symptoms and recurrence or rebound of symptoms for which the BZD had been prescribed is often difficult. The most commonly experienced symptoms of withdrawal—such as anxiety, insomnia, and irritability—are often indistinguishable from previously experienced symptoms associated with underlying conditions.^82^ As discussed previously, the pace of the BZD taper should seek to minimize withdrawal symptoms when possible, and clinicians should treat underlying conditions with evidence-based non-BZD therapies.

**Table 4:** Common Benzodiazepine Withdrawal Signs and Symptoms

General	Affective	Cardiovascular	Gastrointestinal	Neurological	Neuromuscular	Neuropsychiatric	Sleep
• Elevated blood pressure • Headaches • Sweating, night sweats	• Anxiety, panic attacks • Depression, dysphoria • Irritability, agitation, aggression	• Chest pain • Palpitations • Tachycardia	• Abdominal cramps • Diarrhea • Nausea and vomiting	• Cognitive impairment (e.g., poor memory, reduced concentration) • Confusion, delirium* • Perceptual disturbance • Seizures* • Sensory hypersensitivity (i.e., to light, sound, taste, and smell) • Tingling, numbness, altered sensation • Tinnitus	• Coordination, balance problems • Dysesthesia, kinetic disorders • Muscle pain (e.g., tension, weakness, spasms) • Muscle twitches, jerks, and fasciculations • Tremors	• Akathisia, restlessness • Depersonalization, derealization • Psychosis (e.g., paranoia)* • Suicidality and self-harm	• Hypersomnia • Insomnia • Nightmares

Examples of common BZD withdrawal signs and symptoms are grouped by body system. Adapted from Soyka,^2^ Baldwin,^79^ Gold and Ward,^83^ and *The Maudsley Deprescribing Guidelines*.^81^ This table does not represent a comprehensive list of withdrawal symptoms. See *The Maudsley Deprescribing Guidelines* ^81^ and *The Ashton Manual* ^80^ for a more comprehensive list

*Typically associated with abrupt cessation of high doses of BZDs

The development of more severe BZD withdrawal symptoms is associated with use of BZDs with a shorter half-life and fewer active metabolites (e.g., alprazolam), daily use, higher total daily dose, longer duration of use, and history of severe withdrawal.^69,79,84^ A slower initial pace of BZD tapering is likely to be safer and more effective for patients who have a high likelihood of physical dependence and significant risk of withdrawal. As discussed previously, patients should be involved in determining the initial and ongoing tapering pace with clinicians, with the pace ideally agreed upon in a shared decision-making process.

The presence of certain psychiatric symptoms has been associated with an increased likelihood of experiencing more severe withdrawal symptoms, which can present challenges to successful completion of BZD tapering.^59,84^ For example, patients with higher levels of anxiety may have more difficulty with tapering, and patients who exhibit traits associated with borderline, histrionic, or narcissistic personality disorder often experience considerable difficulty discontinuing BZDs (see “Patients with Co-occurring Psychiatric Disorders”).^84^

#### Managing Mild to Moderate Withdrawal Symptoms

Many patients will experience mild to moderate withdrawal symptoms during the BZD taper. If patients experience challenging symptoms, the CGC recommends first pausing or slowing the tapering schedule per Recommendation 11 and incorporating adjunctive psychosocial interventions per Recommendation 10. If pausing or slowing the taper has not been successful, clinicians and patients may decide through a shared decision-making approach to explore adjunctive pharmacological interventions (see “Adjunctive Interventions During the Tapering Process”).

#### Assessing and Managing Seizure Risk

Clinicians should pay particular attention to ascertaining if patients have experienced seizures in the past, as such a history can increase the risk of BZD withdrawal seizures.^85^ Clinicians should also conduct a thorough medication review, as medications that lower the seizure threshold can increase the risk of BZD withdrawal seizures. PDMPs can help detect multiple BZD prescriptions and concurrent prescriptions of controlled medications that lower the seizure threshold. If seizure risk is identified, clinicians can consider a slower taper rate and should have a clear plan for how to address a seizure if it does occur, including the immediate response with appropriate medication.

The CGC noted that clinicians from various medical subspecialties differ in their management of seizure risk. Addiction medicine specialists commonly use adjunctive pharmacotherapies (e.g., levetiracetam, carbamazepine) to prevent seizures in patients experiencing BZD withdrawal who have a history of withdrawal-related seizures. In these instances, addiction medicine clinicians are particularly concerned about the phenomenon of increasing seizure severity with repeated episodes of withdrawal (i.e., kindling). However, neurologists generally do not treat seizure risk prophylactically. As such, the CGC did not come to consensus on management of seizure risk in patients experiencing BZD withdrawal. The CGC recommends that clinicians manage seizures and seizure risk according to current standards of care, which may differ across disciplines.

#### Transitioning to a Longer-Acting Benzodiazepine

Existing CPGs disagree on whether patients who are currently taking a short-acting BZD (e.g., alprazolam) should be transitioned to a longer-acting BZD (e.g., clonazepam, diazepam) for the taper.^86^ Some existing guidance suggests that switching to a longer-acting BZD allows the body “to adjust slowly to a decreasing concentration of the BZD” and, therefore, reduces withdrawal symptoms.^69,80^ Conversely, switching to a longer-acting BZD may not be appropriate for patients who have contraindications (e.g., significant liver dysfunction) and/or are taking multiple medications due to a risk of pharmacodynamic and pharmacokinetic interactions. The CGC suggested that the decision to switch to a longer-acting BZD should be patient-specific, and that clinicians should consider patients’ liver function and concurrent medication use before making a recommendation to switch to a longer-acting formulation (see Box 5).

**Box 5:** Note of Caution: Sedative–Hypnotic Medications

In general, clinicians should avoid transitioning patients from BZDs to other sedative–hypnotic medications (e.g., barbiturates, Z-drugs) with similar risk profiles. Evidence suggests that Z-drugs are associated with a similar increase in the risk of adverse events, including mortality, as BZDs.^87–91^	

Alprazolam tends to be difficult to taper given that it is short-acting and has no active metabolites.^62^ As such, clinicians may consider transitioning patients currently taking alprazolam to a longer-acting BZD for the taper. However, alprazolam may exhibit higher cross-tolerance with other BZDs, and some patients may have challenges with this transition.^62^ When patients have significant withdrawal symptoms in response to the transition to an alternative BZD, clinicians should typically transition patients back to their original BZD medication.

Appendix B compiles dose equivalents in the existing literature. It is important to emphasize that determining the equivalent dose of an alternative BZD is inexact and can vary across patients. Many conversion tools exist (e.g., online, mobile apps, in EHRs); however, unlike with opioid medications, no precise strategies for conversion exist. The widely available equivalent doses which were established based on the average doses of diazepam that patients have reported provide similar symptom management as the previously used BZD. As these equivalent doses were initially based on patient perception, patient experience should be considered when converting between agents. Some patients may require higher doses than the reported equivalent, while others may require lower doses. Transition to an alternative BZD may be more successful if the doses are slowly transitioned over 1–2 weeks rather than 1–2 days.

Issues related to switching to a longer-acting BZD are of particular concern in older adults, who may be at greater risk of medication-related harm because of age-related changes in pharmacokinetics and pharmacodynamics, such as reduced clearance of certain sedative–hypnotic medications and increased sensitivity to CNS effects.^92,93^ Older adults’ decreased hepatic metabolism changes how the body processes and responds to medications, causing them to stay in the body longer and increasing the risk of adverse effects.^92,93^ Chronic BZD use is also a concern for older adults as they are likely to be prescribed multiple medications, increasing their risk of morbidity and mortality from polypharmacy.^94,95^ In a recent scoping review of several international CPGs for BZD tapering,^86^ the two guidelines that did not recommend switching to a longer-acting BZD were focused on older adults.^33,96^ The CGC agreed that switching to a longer-acting BZD for tapering is less likely to be appropriate for older adults.

Some patients with SUD may report nonmedical use of prescribed BZDs or use of nonprescribed BZDs, which can make determining their daily BZD dose difficult. The CGC recommends that clinicians refer these patients for assessment of potential SUD. Unless contraindicated, these patients should typically be transitioned to a long-acting agent due to the uncertainty regarding the strength of the BZDs they are taking.

Guidelines that recommend transitioning to a longer-acting BZD most commonly endorse switching to diazepam or clonazepam, though a few suggest chlordiazepoxide.^86,97^ However, all of these medications are metabolized in the liver and have active metabolites and, thus, should not be used in patients with significant hepatic impairment.^83^ Instead, lorazepam—which is not impacted by hepatic function and does not have active metabolites—is a better agent to use in these patients.^83,86^ As discussed previously, conversion to diazepam equivalents is not straightforward and depends on patient factors such as age, metabolism, and other medications (see Appendix B for estimated BZD dose equivalents).

#### Tapering Strategies

BZDs should not be discontinued abruptly in patients who are likely to have developed physical dependence and are at risk of significant withdrawal symptoms (see Table 3).^33,69,86^ Most existing clinical guidance documents emphasize the importance of gradual dose reductions to discontinue BZD use in these patients.^81,86^ Clinicians can suggest a trial dose reduction for patients who are extremely reluctant or anxious about tapering rather than asking them to commit to a tapering plan. This approach may increase patients’ motivation, self-efficacy, and willingness to continue with tapering.^98^ However, clinicians should clearly communicate any concerns for patients’ safety with ongoing BZD use.

Several BZD tapering strategies have been described in the literature.^86^ Evaluated tapering schedules vary from a faster pace of reductions of 10 to 25% every 1–2 weeks to a slower pace of 5 to 10% every 2–4 weeks, with even more gradual reduction at lower doses when approaching discontinuation.^86^ Clinical trials that reduced doses at a faster pace tended to have high patient dropout rates.^75,76^ Schweizer et al.^75^ noted that 25% weekly dose reductions was too fast for about half of the participants. Oude Voshaar et al.^76^ evaluated the same pace and found that nearly a quarter of participants dropped out. Guidelines that outline specific tapering protocols generally recommend limiting dose reductions to no more than 25% every 2 weeks.^86,97^ The CGC highlighted the importance of considering a patient’s BZD dose, frequency, and duration of use when determining an approach to tapering.

Clinicians should take each patient’s risk–benefit balance into account when developing tapering strategies. A more rapid taper may be indicated for patients who have significant imminent safety risks associated with continued BZD use that will not be mitigated sufficiently with smaller dose reductions. If risks are not imminent, clinicians should consider patient preferences more heavily when developing tapering strategies and seek to minimize risks associated with tapering, including withdrawal symptoms.

Feasibility issues may influence the tapering strategy. When patients are taking the lowest available dose for a given BZD, reducing the dose by 5% or 10% can be challenging. Although some tablets can be accurately cut in half or even quarters with a pill splitter, smaller dose reductions are more difficult to achieve. Clinicians can consider converting the prescription to lower strengths of the same medication as an initial step to facilitate the tapering process. The availability of a greater range of low-dose formulations for commonly prescribed BZDs would help facilitate BZD tapering.

Some available guidance points to the availability of liquid formulations for some BZD medications (e.g., diazepam oral solution concentrate) and the use of compounding pharmacies for custom dosage tablets. However, these options come with a higher cost, and not all patients have access to them. In addition, measuring out liquid doses can be challenging for some patients. These strategies are discussed in detail in *The Maudsley Deprescribing Guidelines*. See Appendix 7 for resources on managing challenging dosage reductions with available formulations.

Patients who have been taking lower doses of BZDs for shorter periods of time may desire or be able to taper from the medication more quickly than recommended in this Guideline. Clinicians can reasonably consider if a faster taper may be indicated or if a taper is necessary for patients whose presentation and history suggest a low risk of significant physical dependence and withdrawal.

Clinicians should also consider patients’ underlying conditions or symptoms for which BZDs are being used to manage when developing tapering strategies. For example, if BZDs have been used to manage anxiety with insomnia, clinicians can recommend tapering the morning dose first. See Appendix 3 for case descriptions and associated sample tapering strategies.

The CGC emphasized that clinicians should engage patients as active partners in a shared decision-making approach to develop and dynamically adjust individualized tapering strategies that reflect a given patient’s goals, needs, and preferences. The FDA also underscored the importance of developing individualized tapering strategies in a 2020 Drug Safety Communication^4^^(2)^:

To reduce the risk of acute withdrawal reactions, use a gradual taper to reduce the dosage or to discontinue benzodiazepines. No standard benzodiazepine tapering schedule is suitable for all patients; therefore, create a patient-specific plan to gradually reduce the dosage, and ensure ongoing monitoring and support as needed to avoid serious withdrawal symptoms or worsening the patient’s medical condition.

#### Adjusting the Taper Strategy

Tapering often does not proceed at the same pace over the entire process; rather, pacing should be flexibly adjusted based on patient response. Although clinicians and patients can prepare for the BZD tapering process by setting realistic expectations around the potential withdrawal and/or rebound symptoms a given patient may be likely to experience, accurately predicting the extent and severity of symptoms that may manifest once tapering is underway is difficult. For this reason, clinicians should monitor patients for signs and symptoms of withdrawal with each dose reduction and counsel them to report any concerning symptoms. Clinicians should discuss this inherent uncertainty with patients so that, together, they can adjust planned tapering strategies as necessary.

Some patients may interpret the emergence of symptoms as evidence that BZD medication is necessary to manage their underlying condition. Clinicians should help patients understand that these symptoms commonly reflect physical dependence. Chronic BZD use leads to changes in BZD receptor expression and response. As the BZD dose is reduced, the BZD receptors slowly adjust. Symptoms should resolve as the receptors return to homeostasis. Clinicians should reassure patients that tapering strategies can be adjusted to address significant symptoms that may occur.

Symptoms can also reflect the reduction in BZD-induced sedation. For example, patients who are taking high doses of BZDs may have increased sleep duration above their age-appropriate sleep needs. As the BZD is tapered, they may return to age-appropriate sleep needs. Patients may be concerned that this reduced sleep indicates insomnia, but it may instead be evidence of previous oversedation with the BZD.

In general, tapering strategies should be adjusted when patients experience significant symptoms related to the taper. Adjustments could include slowing the pace of the taper, making smaller dose reductions, and/or pausing the taper. The CGC noted that clinicians should generally avoid going back up to a previous dose as this can undermine the goal of resetting BZD receptor levels in the brain. However, if patients are experiencing intolerable symptoms that are not addressed adequately by the above strategies, clinicians can consider resuming the previous dose until patients stabilize and are able to continue with the taper.

This Guideline uses two terms to describe an interruption to the planned taper: pausing and maintaining. When tapering is paused, the intent is for patients to remain at the current dose until their symptoms stabilize, and then continue with dose reductions. When patients are ready to resume tapering, clinicians may need to reassess the amount and pace of subsequent dose reductions more frequently. Maintaining refers to circumstances in which no current plan is in place to continue dose reductions; instead, patients are expected to continue taking BZDs at a lower dose (i.e., a partial taper). This may occur when the risks of continuing the taper outweigh the benefits of achieving a lower BZD dose or the benefits of taking the BZD medication now outweigh the risks for a given patient. The dose should be maintained at the reduced level achieved by the partial taper; dose increases should be avoided unless absolutely necessary, such as in response to severe withdrawal symptoms.^69^ The harms of BZDs are dose-dependent.^22,99^ In some cases, maintaining patients at a lower BZD dose may be sufficient to reduce their current risk of harm such that risks no longer outweigh benefits.

Clinicians can use hyperbolic tapering for patients who experience withdrawal symptoms to reduce the likelihood of symptoms with each dose reduction. Hyperbolic tapering is a strategy of nonlinear sequential reduction of a substance, such that dose reductions are smaller and smaller over time.^81^ Each dose reduction is based on the previous dose, not on the dose at the start of the taper. For example, a patient who began a taper on 10 mg diazepam/day with plans for dose reductions of 10% would first reduce their dose to 9 mg/day, then to 8.1 mg/day, then to 7.2 mg/day, then to 6.5 mg/day, and so on. The theory behind hyperbolic tapering is to maintain a consistent impact of each dose reduction on receptor occupancy throughout the taper.^81^ As the hyperbolic taper progresses, clinicians can also slow the pace of the dose reductions to give the BZD receptors more time to adjust. Many patients may benefit from a slower taper as they approach the point of discontinuation. However, as discussed in “Tapering Strategies”, limitations on available dosages may limit feasibility.

The Patient Panel noted that some patients may experience significant withdrawal symptoms even when tapering with 5% dose reductions and may benefit from microtapering. No research was identified that addresses this topic. However, existing guidance provides information on microtapering (see Appendix 7 for additional resources).^81^

#### Taper Duration

Most existing guidance recommend a flexible approach to tapering, reducing the dose at a rate dictated by each patient’s ability to tolerate withdrawal symptoms and allowing the process to take as long as patients need.^2,59,69,79,80,100–102^ The CGC recognized that the tapering process may take a year or more for patients who have been taking BZDs for a long period of time (e.g., years). This Guideline recommends engaging patients as partners; individualizing tapering strategies to each patient’s unique goals, needs, and preferences; and modifying tapering strategies as needed based on a patient’s response to the taper.

#### Follow-Up

A patient’s adjustment to BZD discontinuation and need for clinician support may last well beyond the time it takes for the BZD to be eliminated from the body. Some patients may experience protracted withdrawal symptoms that can last for months or years after the BZD has been discontinued (see “Management of Protracted Withdrawal”).^4^ Although gradual dose reductions and slow tapers may help prevent protracted withdrawal, clinicians should follow up with patients after the BZD has been discontinued to monitor for these symptoms and manage them if they do arise. Other patients, particularly those who have been taking BZD for a long time, may be so accustomed to using BZDs to cope with stress and anxiety that they struggle to avoid returning to BZD use. As such, patients may benefit from ongoing monitoring after the tapering process.

Clinicians should educate patients experiencing protracted withdrawal symptoms on the cause of these symptoms and reassure them that symptoms are likely to resolve with time as their brains adjust to the lack of BZD, which may take months. Psychosocial support (e.g., CBT, peer support) may be helpful for patients during this time. Clinicians should avoid reinstating the BZD but can consider prescribing non-BZD medications for symptom management when patients are unable to tolerate withdrawal symptoms as discussed in the following section.

### Adjunctive Interventions During the Tapering Process

**Recommendations for Adjunctive Interventions**

10. Clinicians should offer patients undergoing BZD tapering behavioral interventions tailored to their underlying conditions (e.g., CBT, CBT-I) or provide them with referrals to access these interventions (***Low Certainty***, Strong Recommendation).
11. Clinicians should first consider pausing or slowing the pace of the BZD taper when patients experience symptoms that significantly interfere with the taper (e.g., sleep difficulty, anxiety), although clinicians can also consider use of adjunctive medications (*Clinical Consensus*, Conditional Recommendation).

**Implementation Considerations**

- Clinicians should educate patients on lifestyle factors that could support BZD tapering (e.g., sleep hygiene, physical activity as appropriate to ability).
- Clinicians can consider other evidence-based approaches such as mindfulness-based interventions.^103^
- Clinicians can consider referring patients to peer specialist services for support during the taper.

#### Rationale

##### Adjunctive Psychosocial Interventions

A systematic review which found gradual tapering supported by adjunctive psychosocial interventions was more effective than gradual tapering alone.^104^ Psychosocial interventions encompass evidence-based behavioral interventions (e.g., CBT, CBT-I; see Appendix 4 for a summary of adjunctive psychosocial interventions). In addition, patients may find approaches tailored to withdrawal-related symptoms helpful (e.g., sleep hygiene for withdrawal-related sleep difficulties, evidence-based mindfulness practices). Some patients may also benefit from peer specialist services when experiencing challenges with tapering. The CGC recommends that clinicians offer adjunctive psychosocial interventions to patients tapering BZDs, especially those whose daily functioning has been negatively impacted by withdrawal symptoms.

A Cochrane review by Darker et al.^105^ found moderate-quality evidence that patients were more likely to have successfully discontinued BZDs at 1 month and 3 months post-treatment when they received CBT during the tapering process. Although CBT has the most evidence, other behavioral interventions that have been studied include MI, direct-to-consumer educational interventions (e.g., letters and booklets mailed to patients), relaxation therapy, and counseling via telemedicine.^56,105^ A recent meta-analysis by Lynch et al.^106^ showed a significantly higher rate of BZD discontinuation at 6 months and 12 months among patients who received a brief intervention delivered in primary care (e.g., short consultation with prescribers, letters from prescribers recommending discontinuation) compared to those receiving usual care, with risk ratios of 2.73 and 3.41, respectively, favoring the intervention. See Table 10 in Appendix 5 for the full Evidence to Decision table on CBT.

Sleep hygiene interventions may also help support successful tapering. Sleep hygiene refers to the sleep environment and behaviors around sleep—such as adopting a nightly routine, following a sleep schedule, avoiding caffeine and alcohol near bedtime, and avoiding napping during the day—that are conducive to optimizing restorative sleep.^107,108^ Although sleep hygiene education is not a standalone treatment for primary insomnia, some evidence suggests it may help support the tapering process.^107^ For example, incorporating sleep hygiene education and psychosocial support during BZD tapering has been shown to lead to short-term reductions in BZD use as well as long-term discontinuation in older adults.^107^

Peer specialist services are another resource that can support patients during BZD tapering. Peer specialists are individuals who have relevant lived experience with BZD tapering, mental health conditions, and/or SUD and are trained to provide services that promote recovery, foster resilience, and build on patients’ strengths as they work through the BZD tapering process.^109^ Peer specialist services can be delivered one-on-one or in group settings, as well as in-person or virtually.

The most important considerations when considering adjunctive psychosocial interventions during tapering are an individual patient’s treatment preferences, their response to the BZD tapering process, and their access to adjunctive services.

##### **Adjunctive Pharmacological Interventions**

Considerable disagreement exists in the literature on the utility of pharmacological interventions as an adjunct to BZD tapering. Existing clinical guidelines that endorse adjunctive medications do not offer clear guidance on implementation (e.g., dosing, duration).^86^ In a Cochrane review, Baandrup et al.^77^ were unable to draw conclusions on the effectiveness and safety of various medications in facilitating BZD discontinuation because the quality of the evidence was low or very low and with a high risk of bias. The systematic literature review for this CPG review yielded 28 RCTs on various adjunctive pharmacological interventions, including over-the-counter aids such as melatonin, to support BZD tapering (see Table 7 in Supplementary Material for methodology). The CGC considered the evidence for medications that are currently available in the USA.

A few small studies have suggested the anticonvulsant carbamazepine might have limited effectiveness as an adjunct during the BZD tapering process to reduce anxiety and withdrawal symptoms.^77,110–112^ The CGC considered these findings and agreed there is no robust evidence that carbamazepine facilitates discontinuation and, thus, it is not recommended as an adjunct medication for BZD withdrawal management.

Buspirone had the most evidence in the systematic literature review. A total of six studies compared buspirone to placebo to support the tapering process in adults.^113–118^ While the combined evidence suggested a slight benefit for buspirone on the outcome of BZD discontinuation, the CGC cited methodological issues that would limit applicability. For example, the CGC noted that many of the studies did not utilize a therapeutic dose of buspirone, and outcomes were inconsistently measured. They also discussed that the risk of drug–drug interactions should raise the threshold for recommending a medication with relatively low likelihood of benefit. The CGC agreed that although buspirone may be helpful in some patients, there was not adequate evidence to single it out as a recommended pharmacological intervention for BZD tapering, giving the impression that it is superior to other potentially useful agents.

After carefully considering existing evidence on various pharmacological interventions, the CGC agreed that no single medication had enough data to support recommending it. The CGC emphasized that the primary clinical strategy for supporting an effective taper is going slow and adjusting based on the patient’s response. The recommendations seek to highlight the importance of first pausing or slowing the taper if a patient is experiencing taper-related symptoms, minimizing polypharmacy where possible. If a slower taper does not control patient symptoms, medications may be indicated and those decisions should be made on a case-by-case basis.

The CGC noted that although gabapentin and pregabalin may be useful in certain circumstances, they have potential for misuse and should not be considered prior to other potential adjunctive medications.

The Patient Panel emphasized that some patients who are experiencing protracted withdrawal have trouble tolerating adjunctive psychoactive medication. In their collective experience, medications and supplements that act directly or indirectly on GABA receptors (e.g., SSRIs, gabapentin, magnesium) can exacerbate and extend the duration of protracted withdrawal symptoms. They emphasized the importance of a slow taper and giving the brain time to recover.

Clinicians should first consider whether patients’ symptoms are likely to be primarily attributable to BZD withdrawal or underlying conditions. The CGC noted that distinguishing BZD withdrawal symptoms from recurrence of symptoms related to underlying conditions can be difficult. Based on clinical experience, symptoms that change in parallel with BZD dosage changes and/or resolve rapidly after pausing the taper are more likely to be related to BZD withdrawal. However, if symptoms do not resolve after pausing the taper, it may be unclear whether the symptoms are related to protracted withdrawal (which can last for months or years), worsening or new physical or mental health conditions (e.g., anxiety or sleep-related disorders), or a combination of withdrawal and underlying conditions. If the patient experiences physical or psychological symptoms that are distinct from symptoms of the underlying condition (e.g., neurological or sensory symptoms), they may be related to protracted withdrawal.^81^ In these instances, more frequent monitoring may be warranted. Clinicians may also consult with specialists appropriate to patients’ symptoms.

Although evidence for medications to treat BZD withdrawal symptoms is lacking, treating symptoms of underlying conditions can be effective (e.g., SSRIs for GAD; see Appendix 7 for a list of CPGs on the management of conditions for which BZDs are commonly prescribed). Clinicians should attempt to optimize evidence-based treatment for any psychiatric disorder prior to or, if clinically indicated (e.g., due to imminent risks related to continued BZD use), concurrent with the taper. Clinicians should attempt to minimize the risks of polypharmacy whenever possible when selecting adjunctive medications (see Appendix 5).

### Management of Severe or Complicated Withdrawal Symptoms

**Recommendations for Management of Severe or Complicated Withdrawal Symptoms**

12. Clinicians should manage patients experiencing severe or complicated withdrawal in inpatient or residential medically managed settings (e.g., residential withdrawal management program) with:
  a Monitoring for signs and symptoms of BZD withdrawal, including regularly measuring vital signs and using structured assessment tools (*Clinical Consensus*, Strong Recommendation)
  b Assessments for seizure risk and managed as appropriate (*Clinical Consensus*, Strong Recommendation)

13. Tapering with very long-acting agents such as phenobarbital:
  a Can be considered for BZD withdrawal management in inpatient settings (***Low Certainty***, Strong Recommendation).
  b Should only be conducted by or in consultation with clinicians experienced in the use of these agents for the purpose of BZD withdrawal management (*Clinical Consensus*, Strong Recommendation).
14. Clinicians should avoid rapid BZD reversal agents such as flumazenil for the purpose of BZD tapering due to risks for refractory seizure, cardiac dysrhythmias, and other adverse effects (*Clinical Consensus*, Strong Recommendation).
15. Clinicians should avoid general anesthetics such as propofol or ketamine for the purpose of BZD tapering (*Clinical Consensus*, Conditional Recommendation).

**Implementation Considerations**

- Tapering initiated in an inpatient or residential medically managed level of care may be continued in a less intensive level of care once it is safe to do so.
- When tapering with very long-acting agents, discharge planning should include an outpatient follow-up appointment, ideally within 7 days.
  - Clinicians should assess patients for ongoing signs or symptoms related to discontinuation of the BZD, including re-emergence of symptoms for which the BZD was originally prescribed.
  - Clinicians should consider medications and/or behavioral interventions to address ongoing signs or symptoms related to discontinuation of the BZD.

#### Rationale

##### Monitoring During Withdrawal Management

Although most patients can successfully taper from BZD in outpatient settings, inpatient or medically managed residential settings may be indicated if patients experience severe acute BZD withdrawal. As with any sedative–hypnotic medication, seizure and delirium are two of the more serious adverse events that can occur as part of withdrawal. Clinicians should prioritize assessment and monitoring for seizure risk and other clinically significant symptoms during BZD withdrawal management. Patients who are experiencing or imminently expected to experience severe acute symptoms of BZD withdrawal should be managed in settings appropriate to their risk (see “Level of Care Considerations”).

Regular patient monitoring is critical during withdrawal management. What constitutes regular monitoring depends on the treatment setting. Inpatient and other medically managed settings where withdrawal management occurs (i.e., specialty medically managed SUD treatment settings) typically have protocols for monitoring withdrawal. The CGC noted that the two most important items to monitor are vital signs and patient-reported withdrawal symptoms.

Scales designed for monitoring BZD withdrawal symptoms exist, including the Clinical Institute Withdrawal Assessment Scale-Benzodiazepines (CIWA-B)^119^ and the BZD Withdrawal Symptom Questionnaire (BWSQ).^120^ However, both these scales were developed with a small number of patients and little to no evidence of validation was found for either; as such, they are not used frequently in clinical practice. Although no validated scales exist for monitoring BZD withdrawal symptoms, the CGC noted collecting structured information can help improve objectivity and consistency in symptom measurement.

##### Inpatient Withdrawal Management

As discussed in “Level of Care Considerations” clinicians should consider inpatient BZD withdrawal management when:

- Patients are at imminent risk of significant harm from continued BZD use that is unlikely to be mitigated rapidly by the taper’s initial dose reduction
- Patients have co-occurring physical or mental health conditions that makes BZD tapering unsafe in outpatient settings
- Patients are experiencing or imminently expected to experience severe or complicated withdrawal

As with any tapering plan, BZD tapering in inpatient settings should focus on providing supportive care and managing and minimizing withdrawal symptoms and co-occurring conditions, as appropriate. Patients who initiate BZD tapering in inpatient or residential medically managed settings may complete their taper in outpatient settings if appropriate.

##### Tapering with Very Long-Acting Agents

Some limited evidence exists for a loading dose strategy using very long-acting agents that modify responses to gamma-aminobutyric acid (GABA) such as phenobarbital to initiate a BZD taper to discontinuation in patients with BZD use disorder.^121^ Phenobarbital is a barbiturate with a very long half-life (80–120 h) that results in a gradual taper of effects after the medication is discontinued. The CGC emphasized that this approach should be limited to situations involving imminent patient safety concerns that cannot be appropriately mitigated by an initial dose reduction (see “Level of Care Considerations”). This approach may also be effective for patients with SUD who have been unable to accomplish a gradual taper in outpatient settings. In some instances, patients may request this type of approach due to a desire to discontinue BZD use quickly.^122^

Two retrospective studies that cumulatively evaluated outcomes from over 650 patients found phenobarbital-based protocols for tapering in inpatient settings to be safe and effective.^121,123^ A retrospective case series by Kawasaki et al.^123^ of 310 patients who were treated with a 3-day phenobarbital protocol found that, while 27% of patients experienced sedation and 17% self-discharged from treatment, none experienced falls or seizures and only 1% experienced delirium. A more recent chart review by Sartori et al.^121^ of 355 patients who underwent a 6-day phenobarbital protocol found that no patients experienced seizures, falls, or sedation, although 5% self-discharged from treatment. Although both studies had noted limitations as retrospective studies with no comparison group or long-term follow up data, they suggest phenobarbital-based protocols may be a reasonable approach to BZD tapering for select patients. See Table 3 in Supplemental Material for the full Evidence to Decision table on phenobarbital for BZD tapering.

The Patient Panel expressed significant concerns about the potential harms of tapering with phenobarbital, including severe protracted withdrawal. Current research in this area is insufficient; however, the high self-discharge rate in available studies should be taken into account.

In general, tapering with very long-acting medications should be conducted in inpatient or medically managed residential settings due to the increased risk of overdose associated with barbiturate medications (e.g., phenobarbital). In limited instances, specialist clinicians (e.g., addiction medicine) with appropriate experience and the necessary capacity for adequate patient monitoring can use these medications in medically managed intensive outpatient settings (e.g., ASAM Criteria Level 2.7) to support BZD tapering in patients with SUD. As with other tapering strategies, adjunct medications may be helpful during the tapering process. Examples of tapering with very long-acting medications can be found in Appendix 3.

##### **Discharge Planning**

Discharge planning is critical following BZD withdrawal management in inpatient or medically managed residential settings. If tapering is not completed during the inpatient or residential stay, clinicians should ensure that patients have access to any medications needed for continuing the tapering process, including BZDs. Discharge planning should include an outpatient follow-up appointment, ideally within 7 days, and referral for co-occurring physical and mental health conditions (e.g., insomnia) as needed.

During the follow-up appointment, clinicians should assess patients for ongoing signs and symptoms related to the reduction or discontinuation of the BZD, including recurrence, rebound, and residual withdrawal symptoms. See “Adjusting the Taper Strategy” and “Management of Protracted Withdrawal” for further discussion.

##### **Other Pharmacological Interventions**

Flumazenil, a GABA-A receptor antagonist, is effective at reversing CNS and respiratory depression due to BZD overdose. Recent RCTs have suggested that low-dose flumazenil may be effective for facilitating BZD discontinuation, especially among patients taking high doses of BZDs.^124,125^ Despite these findings, the CGC had concerns about the high potential for refractory seizures, cardiac dysrhythmias, and other adverse effects when using flumazenil.^126^ Therefore, the CGC agreed that flumazenil should not be utilized for the purposes of BZD tapering. Similarly, very limited evidence was found for use of anesthetics such as ketamine and propofol for facilitating BZD withdrawal.^127^ Both ketamine and propofol are associated with a significant risk of increased respiratory depression when combined with BZDs, and no evidence supports their use for routine BZD tapering. Therefore, the CGC agreed that the risks of anesthetics (e.g., ketamine, propofol) for BZD tapering outweigh potential benefits and could not be recommended. Similarly, there is no evidence for the use of medications used for procedural sedation (e.g., dexmedetomidine) in BZD withdrawal management.

### Management of Protracted Withdrawal

Some patients may experience protracted symptoms of withdrawal after BZD discontinuation (see Box 6).^81,128^ Protracted withdrawal may result from a combination of physical and psychological BZD dependence and the neurological effects of BZDs.^129^ Longer-term BZD use and use of high-dose, rapid-acting BZDs increase the risk of protracted withdrawal; however, these post-acute symptoms can also occur after discontinuation of low-dose BZDs.^80,130,131^ Protracted symptoms persist beyond the expected elimination of the BZD from a patient’s system after discontinuation (e.g., 4–6 weeks), with some patients experiencing these symptoms for months or years.^78,130,132^ Protracted withdrawal symptoms can adversely affect patients’ relationships, family life, careers, and mental health. In a convenience sample of 1,200 individuals recruited through several patient-facing internet and social media sites with content tailored to patients facing challenges with BZD discontinuation, Reid Finlayson et al.^128^ found that 54% of respondents reported suicidal thoughts or attempted suicide after BZD discontinuation. Although limited research exists on protracted withdrawal and BZD discontinuation, the CGC agreed it causes significant harms for a subset of patients.

The patient panel emphasized the importance of appropriate recognition and accurate diagnosis of protracted withdrawal. They noted that when clinicians do not recognize patients’ symptoms as protracted withdrawal, they may recommend medications for symptom management that have direct or indirect effects on GABAergic signaling that can exacerbate or lengthen the duration of these symptoms. Current guidance suggests gradual dose reductions and slow tapers may reduce the risk of protracted withdrawal symptoms.^80^

**Box 6** Protracted Withdrawal Symptoms

**Table:** 

Protracted withdrawal symptoms may include but are not limited to: • Psychological: anxiety, depression, agitation, anhedonia, hallucinations • Neurological: poor memory and cognition, distractedness, formication, paresthesia, tinnitus • Neuropsychiatric: akathisia, psychosis • Other: motor disturbances, gastrointestinal disturbances, insomnia, dizziness	

Some researchers have proposed that some protracted withdrawal symptoms may be better categorized as neurological dysfunction given the potential neurological risks associated with BZD use.^133^ The term benzodiazepine-induced neurological dysfunction (BIND) has been proposed to describe persistent neurological disturbance and CNS damage that may emerge from BZD use.^133^ However, neurological mechanisms of protracted BZD withdrawal are not well established and require further research.

## POPULATION-SPECIFIC CONSIDERATIONS

### Patients Co-prescribed Benzodiazepines and Opioids

**Recommendations for Patients Co-prescribed Benzodiazepines and Opioids**

16. Because all patients co-prescribed BZDs and opioids are at increased risk of respiratory depression, clinicians should assess the risks and benefits of continued BZD prescribing with every related clinical encounter or prescription renewal and at least every 3 months (*Clinical Consensus*, Strong Recommendation).
17. Clinicians should offer to provide or prescribe opioid overdose reversal medication (e.g., naloxone) for all patients co-prescribed BZDs and opioids (*Clinical Consensus*, Strong Recommendation).
18. Clinicians should consider additional strategies for mitigating risk, including using the lowest effective doses of BZD and opioid medications and optimizing non-opioid interventions (*Clinical Consensus*, Strong Recommendation).

**Implementation Considerations**

- Prior to initiating a BZD taper for patients who are co-prescribed BZDs and opioids, clinicians should seek to coordinate care with other clinicians who are prescribing BZDs or opioids to a given patient. This may entail obtaining releases or other agreements for clinicians to contact other prescribers and/or consulting the PDMP.
- Clinicians should conduct risk–benefit assessments more often when patients have additional risk factors for adverse events related to concurrent BZD and opioid use.^134^ Additional risk factors may include but are not limited to having an SUD, a bipolar disorder, or schizophrenia and/or taking fentanyl, morphine, or methadone.^135,136^

#### Rationale

Although not generally recommended, patients with chronic pain are commonly prescribed BZD and opioid medication for pain management concurrently.^137^ Patients prescribed this combination of medications tend to be on relatively higher doses of opioids and report higher levels of pain and lower self-efficacy for pain management.^138^ They also have greater healthcare utilization, especially ED visits.^138^ Finally, these patients are at greater risk of nonmedical substance use and co-occurring psychiatric conditions compared to patients who are prescribed opioids but have never used BZDs.^138^

Patients taking both opioids and BZDs may be prescribed these medications by different clinicians.^137^ When the risks associated with the combined use of these medications outweigh the benefits, clinicians should engage in shared decision-making with patients to determine which medication to taper. Prior to initiating a BZD taper, clinicians should attempt to coordinate care with patients’ other prescribers. The CGC noted that reaching other clinicians may be challenging. Clinicians can consider coordinating with payers or pharmacies, as they may have alternative mechanisms for communicating with other clinicians involved in a patient’s care.

Patients prescribed both opioids and BZD comprise a high-risk population. Clinicians should use caution when prescribing opioid pain medication and BZDs concurrently and consider whether the risks of concurrent use of opioids with other CNS depressants outweigh the benefits. It is important to note that use of BZDs is not a reason to withhold or suspend treatment with methadone or buprenorphine for the treatment of opioid use disorder (OUD; see “Patients with Benzodiazepine and Other Substance Use Disorders”).

As discussed in Recommendation 1, the CGC recommends that clinicians review the risks and benefits of continued BZD prescribing for patients who take both opioids and BZDs at least every 3 months or at every related clinical encounter or prescription renewal, whichever is sooner. Clinicians should conduct more frequent risk–benefit assessments for patients who have additional risk factors for adverse events. The Risk Index for Overdose or Serious Opioid-induced Respiratory Depression (RIOSORD) is a tool that can be used for this purpose (see Box 7).^135,136^ According to the RIOSORD, the most significant risk factors include having an SUD, a bipolar spectrum disorder, or schizophrenia and/or taking fentanyl, morphine, or methadone.^135,136^

**Box 7** The RIOSORD

**Table:** 

The RIOSORD is a screening instrument designed to provide clinically practical guidance for safer opioid prescribing. It was originally developed using administrative healthcare data from a large sample of patients served by the US Veterans Health Administration (VHA) and validated using a health plan claims dataset with data from over 115 million individuals.^135,136^ The risk assessment looks at co-occurring SUD, mental health diagnoses, and biomedical conditions, as well as the type and formulation of opioids used and co-prescribing of BZDs and other medications. The RIOSORD showed strong predictive accuracy in both datasets.	

Clinicians should consider additional strategies for mitigating risk, including using the lowest effective doses of BZD and opioid analgesic medications and optimizing non-opioid interventions to manage pain. As emphasized in the *2022 CDC Clinical Practice Guideline for Prescribing Opioids for Pain*^139^^(11)^:

When opioids are initiated for opioid-naïve patients with acute, subacute, or chronic pain, clinicians should prescribe the lowest effective dosage. If opioids are continued for subacute or chronic pain, clinicians should use caution when prescribing opioids at any dosage, should carefully evaluate individual benefits and risks when considering increasing dosage, and should avoid increasing dosage above levels likely to yield diminishing returns in benefits relative to risks to patients.

The CGC recommends that clinicians use the lowest effective dose of BZDs and follow the CDC guidelines for minimizing risks related to opioid prescribing.^139^ This includes minimizing opioid doses where possible and optimizing non-opioid interventions for managing pain, such as nonpharmacological treatments for pain management, including exercise, mindfulness-based interventions, and CBT.^139^ The CDC guideline, and the joint US Department of Veterans Affairs (VA) and US Department of Defense (DoD) *Guideline on Chronic Pain Prescribing* also recommend that clinicians consider using buprenorphine, a partial opioid agonist with reduced risk of overdose, to manage pain in patients at risk of withdrawal or overdose, including those who are co-prescribed BZDs.^139,140^ Patients at risk of opioid overdose should be provided with or prescribed opioid overdose reversal medication (e.g., naloxone; see “Harm Reduction”).

### Recommendations for Patients with Benzodiazepine and Other Substance Use DisorderPatients with Benzodiazepine and Other Substance Use Disorders

**Recommendations for Patients with Benzodiazepine and Other Substance Use Disorders**

19. Clinicians should consider more frequent assessments of the risks and benefits of continued use of BZDs for patients with co-occurring SUDs and/or other co-occurring addictions (e.g., behavioral addictions) who have a prescription for BZD medication compared with the general guidance in Recommendation 1 (*Clinical Consensus*, Strong Recommendation).
20. When tapering BZD medication in patients with SUD, clinicians should manage the underlying SUD concurrently with the BZD taper (*Clinical Consensus*, Strong Recommendation).
21. Clinicians should not use BZD prescribing or tapering considerations as a reason to discontinue or disrupt a patient’s medications for SUD treatment, including buprenorphine and methadone (*Clinical Consensus*, Strong Recommendation).
22. Following the taper, clinicians should continue to monitor and treat the underlying SUDs or refer patients to an appropriate level of care for continuing care (*Clinical Consensus*, Strong Recommendation).
23. Clinicians should offer patients harm reduction services or provide them with referrals to access these services.
  a Clinicians should provide opioid overdose reversal medication (e.g., naloxone) and related education (*Clinical Consensus*, Strong Recommendation).
  b Clinicians can consider providing drug checking or other safe use supplies (e.g., fentanyl test strips, xylazine test strips, sterile syringes) and related education (*Clinical Consensus*, Conditional Recommendation).

**Implementation Considerations**

- Clinicians should refer patients with SUD who are undergoing BZD tapering for SUD treatment in parallel with the BZD taper. Care should ideally be coordinated between the clinicians providing SUD treatment and managing the BZD taper, when applicable.
- Clinicians should consider using existing standards for level of care recommendations such as *The ASAM Criteria* when considering treatment setting for patients with SUD (Clinical Consensus, Strong Recommendation).
- Clinicians may consider conducting BZD tapers in residential or inpatient settings for patients with SUD who are unlikely to participate effectively in outpatient tapering.
- As discussed in *Tapering with Very Long Acting Agents*, tapering with phenobarbital should typically be conducted in acute care settings (i.e., hospital or ED) or medically managed residential settings (e.g., *The ASAM Criteria* Level 3.7). However, for patients with SUD, tapering with phenobarbital may also be conducted in outpatient settings with extended nurse monitoring (e.g., *The ASAM Criteria* Level 2.7, where nurse monitoring is available during the day) by or in consultation with clinicians experienced in the use of these medications for BZD tapering.
- Clinicians can consider using toxicology testing to support risk–benefit assessments for patients with SUD if indicated based on clinical concern (see “Drug Testing”).

#### Rationale

Some patients with BZD use disorder may be able to successfully taper the BZD in outpatient settings. However, other patients—such as those who are taking very high doses (e.g., supratherapeutic doses) of BZD and/or using other substances—may require a more intensive level of care. For example, BZD tapering for patients with SUDs who are at high risk of medical instability or severe withdrawal or have a history of withdrawal-related seizure should be initiated in inpatient or medically managed residential settings because of the availability of 24-h nurse monitoring and medical care to support stabilization and withdrawal management.^141^ *The ASAM Criteria* provides guidance on determining the appropriate level of care for patients with SUD (see Box 8).^141^

**Box 8** *The ASAM Criteria*: Levels of Care

**Table:** 

First published in 1991, *The ASAM Criteria* offers a standardized, evidence-based way of determining the appropriate level of SUD treatment services based on an individual’s needs and circumstances. A multidimensional assessment is used to determine the most appropriate level of care based on intoxication and withdrawal-related risks; need for addiction medications; co-occurring biomedical, psychiatric, and cognitive conditions; substance-use related risks; and recovery environment considerations. *The ASAM Criteria* describes SUD treatment as a continuum marked by 4 broad levels of care: outpatient, intensive outpatient, residential, and inpatient. The decimal number expresses gradations of intensity and types of care provided. Level *x*.7 programs are medically managed programs that provide withdrawal management, including management of BZD withdrawal, and biomedical services along with integrated psychosocial services. • Level 1: outpatient treatment • Level 1.5: outpatient therapy • Level 1.7: medically managed outpatient • Level 2: intensive outpatient/high-intensity outpatient treatment • Level 2.1: intensive outpatient • Level 2.5: high-intensity outpatient • Level 2.7: medically managed intensive outpatient • Level 3: residential treatment • Level 3.1: clinically managed low-intensity residential • Level 3.5: clinically managed high-intensity residential • Level 3.7: medically managed residential • Level 3.7 BIO: biomedically enhanced medically managed residential • Level 4: medically managed inpatient treatment	

For more information, see https://www.asam.org/asam-criteria

#### Assessing Risks and Benefits of Continued Benzodiazepine Prescribing

Clinicians should review BZD use frequently for patients who have a history of SUDs, as these individuals are at increased risk of developing SUDs to other substances compared to those without a history of SUD.^142^ In addition, patients who use BZDs and have co-occurring alcohol use disorder (AUD) or OUD are at higher risk of morbidity and mortality because of the cross-tolerance and combined CNS and respiratory depressant effects of these substances.^23,52^ Clinicians should carefully consider these risks when determining the appropriateness of continued BZD prescribing.

#### Considerations for Benzodiazepine Tapering in Patients with Substance Use Disorder

Abrupt cessation of BZDs is dangerous. The CGC recommends clinicians develop gradual tapering strategies that are individualized based on a patient’s response. If more rapid tapering is indicated—for example, due to imminent safety risks or when alternate treatment options have been unsuccessful—clinicians can consider use of very long-acting agents (see “Tapering with Very Long-Acting Agents”). Clinicians should consider patients’ psychosocial concerns and co-occurring disorders when determining the appropriate timing of BZD tapering.

Tapering can be complicated when patients have been obtaining BZDs from the illicit drug market, where counterfeit pills can include novel synthetic BZDs (e.g., etizolam, flubromazolam). These novel synthetic BZDs have not been well studied and may not be detected with standard drug testing or toxicology assays. The European Union Drugs Agency’s *New benzodiazepines in Europe – a review* provides helpful information on emerging new BZDs.^143^ In addition, the US Drug Enforcement Administration (DEA) tracks emerging threats related to BZDs.^144^ Determining an equivalent BZD dose to begin tapering is complicated when patients are taking BZDs from the illicit drug market. In general, clinicians should titrate the BZD dose to the minimum dose necessary to control a patient’s withdrawal symptoms and taper from that point. Clinicians should consider residential treatment if patients need after-hours clinical monitoring or medical management to support safe and effective BZD tapering.

Counterfeit BZD pills may also contain HPSOs (e.g., fentanyl). As such, patients may be unaware they are at risk of opioid withdrawal. Clinicians should monitor patients who have been using nonprescribed BZDs for signs and symptoms of opioid withdrawal. These patients should also be provided with or prescribed opioid overdose reversal medications (e.g., naloxone; see “Harm Reduction”).

If BZD tapering is indicated, clinicians should manage the underlying SUD in parallel with the taper. Clinicians should refer patients to an appropriate level of care for SUD treatment concurrent with BZD tapering. Some SUD treatment programs may be able to take over management of BZD tapering.^145^ Patients with OUD should typically be initiated and stabilized on medications for OUD (MOUD) prior to initiating a BZD taper, and the MOUD dose should be kept stable throughout the BZD tapering process.^145,146^ Clinicians should provide psychosocial interventions (e.g., psychotherapy, counseling, psychoeducation) to treat underlying SUDs in parallel with pharmacotherapy.^145^ As emphasized in *The ASAM National Practice Guideline for the Treatment of Opioid Use Disorder: 2020 Focused Update*^145^:

The use of benzodiazepines and other sedative–hypnotics should not be a reason to withhold or suspend treatment with methadone or buprenorphine. While the combined use of these medications increases the risk of serious adverse effects, the harm caused by untreated opioid use disorder can outweigh these risks.

Monitoring patients during and after BZD tapering is a key aspect of clinical management for successful BZD discontinuation. Approaches to reduce return to BZD use include providing ongoing treatment of underlying SUDs and co-occurring physical and mental health conditions, engaging with recovery support services (e.g., peer support), and addressing environmental risk factors (e.g., housing instability, lack of a recovery-supportive network).

#### Drug Testing

Although drug testing can help detect the use of substances, urine immunoassays for BZDs have limited sensitivity. These immunoassays vary by lab and may only detect select agents. Some are not sensitive enough to detect therapeutic doses of BZDs, and performance of the tests vary depending on the manufacturer.^147^ Interpretation of test results can be complicated by the presence of BZD metabolites, as some metabolites are themselves parent compounds.^148^ For this reason, urine drug screening for BZDs carries an increased risk of false negatives, and confirmatory gas chromatography–mass spectrometry (GCMS) testing is often indicated. Although confirmatory GCMS testing has higher sensitivity, even for low BZD concentrations, and specificity is virtually 100%, it does not detect all BZDs. Clinicians should be familiar with the accuracy and limitations of these assays.

Because of the high risk of false negatives, it is important for clinicians to generally trust patients’ self-reports regarding their BZD use, even if they test negative for BZDs. This is particularly important for patients in inpatient, residential, or correctional settings, who may be placed at significant risk of harm with abrupt discontinuation of BZDs.

The application and frequency of drug testing should be determined by a patient’s clinical needs and the treatment setting. Multiple existing guidance documents emphasize that clinicians should not use drug test results punitively, rather, clinicians should use test results to engage patients therapeutically and inform treatment plans.^79,101,145^

#### Harm Reduction

In most areas of the country, heroin, cocaine, methamphetamine, and counterfeit prescription drugs, including counterfeit BZDs, are commonly contaminated with HPSOs (e.g., fentanyl), presenting significant risk of overdose. This risk is exacerbated by BZD use. All patients who may use opioids, whether intentionally or unintentionally, should be educated about this risk and given or prescribed opioid overdose reversal medication (e.g., naloxone). Clinicians should assess each patient’s individual harm reduction service needs and connect them to available community resources (e.g., harm reduction organizations) for provision of services (e.g., education, safe use supplies [e.g., drug checking kits, fentanyl test strips, sterile syringes]) as appropriate based on their patterns of substance use. Clinicians can also consider counseling patients on other harm reduction strategies, such as not using substances alone and using a test dose first. Harm reduction practices can also be useful when patients decline referrals for SUD treatment. Clinicians can consult the Substance Abuse and Mental Health Services Administration’s (SAMHSA) *Harm Reduction Framework* for more information regarding best practices.^149^

### Patients with Co-occurring Psychiatric Disorders

**Recommendations for Patients with Co-occurring Psychiatric Disorders**

24. Clinicians should optimize evidence-based treatment for any psychiatric disorder prior to the taper or concurrently if clinically indicated (*Clinical Consensus*, Strong Recommendation).
25. Clinicians should strongly consider tapering BZD medication in patients with PTSD (*Clinical Consensus*, Strong Recommendation).
26. Clinicians should monitor sleep closely during BZD tapering in patients with mood or psychotic disorders, particularly for patients with bipolar disorder as sleep disturbance can trigger episodes of mania (*Clinical Consensus*, Strong Recommendation).
  a If patients with a mood and/or psychotic disorder experiences significant sleep disturbance, clinicians should pause the taper until the symptoms resolve due to the risk of destabilization (*Clinical Consensus*, Strong Recommendation).

**Implementation Considerations**

- Clinicians should refer patients with psychiatric disorder who are undergoing BZD tapering for psychiatric treatment in parallel with the BZD taper. Care should ideally be coordinated between the clinicians providing psychiatric treatment and managing the BZD taper, when applicable.
- Clinicians should consider using existing standards for level of care recommendations such as the Level of Care Utilization System (LOCUS) when considering treatment setting for patients with psychiatric disorders.
- Clinicians can consider offering patients with psychiatric disorders behavioral interventions (e.g., CBT-I with sleep hygiene education) or providing them with referrals to access these interventions.
- Clinicians can consider consulting with clinicians with psychiatric expertise when tapering BZDs in patients with co-occurring psychiatric disorders.

#### Rationale

Many patients with psychiatric disorders are able to taper from BZDs in outpatient settings, but some may require a more intensive level of care. BZD tapering may exacerbate or cause recurrence of psychiatric symptoms that may warrant more intensive clinical oversight.^2,150^ Clinicians should consider any underlying psychiatric conditions and relevant treatment history, prior to beginning a BZD taper. Clinicians can consider using the LOCUS for guidance determining the appropriate treatment setting for patients with psychiatric disorders (see Box 9).

**Box 9** Level of Care Utilization System: Levels of Care

**Table:** 

Developed in the 1990 s by the American Association for Community Psychiatry (AACP), the LOCUS offers a standardized, evidence-based way for connecting adults with mental health services based on their individual needs and circumstances. A multidimensional assessment is used to determine the most appropriate level of care for an individual based on their risk of harm; functional status; co-occurring medical, addictive, and psychiatric conditions; recovery environment; treatment and recovery history; and engagement and recovery status. The LOCUS describes seven levels of care of different service intensities, including: • Basic services: prevention and health maintenance • Level 1: recovery maintenance and health management • Level 2: low-intensity community-based services • Level 3: high-intensity community-based services • Level 4: medically monitored non-residential services • Level 5: medically monitored residential services • Level 6: medically managed residential services	

For more information, see https://www.communitypsychiatry.org/locus

Patients who have used BZDs for a long time may be reluctant to taper the medication due to fear of experiencing adverse effects related to discontinuation.^66,151,152^ As BZD tapering can lead to rebound psychiatric symptoms (e.g., anxiety, insomnia), clinicians should optimize evidence-based treatments for any co-occurring psychiatric disorders prior to initiating a BZD taper or concurrently if clinically indicated (e.g., due to significant imminent risks related to continued BZD use).^153,154^ Non-BZD therapies such as SSRIs, CBT, and other evidence-based interventions may be appropriate alternatives to BZD for many patients (see Appendix 4).^155–157^ Clinicians should also consider evidence-based suicide screening such as the Columbia Suicide Severity Rating Scale (C-SSRS) or Ask Suicide-Screening Questions (ASQ) tool for patients at risk.^158,159^

Clinicians should educate patients regarding potential rebound psychiatric symptoms and how they will be managed and offer or refer for appropriate mental health services. As discussed in the “Adjunctive Interventions During the Tapering Process”, providing behavioral interventions during the BZD taper is associated with successful discontinuation of BZD.^155–157^

#### Patients with PTSD

The VA recommends that clinicians avoid prescribing BZDs to patients with symptoms of PTSD and provides guidance on alternative treatments for management of anxiety and insomnia in these patients.^160^ BZDs are ineffective for the treatment of PTSD; they do not reduce the core symptoms of PTSD or improve PTSD-related sleep dysfunction.^161,162^ BZD use is associated with an increased risk of substance use, depression, and aggression; increased PTSD severity; and decreased efficacy of trauma-focused psychotherapy.^163^ When tapering BZD in patients with PTSD, clinicians should consider that withdrawal from BZDs can worsen existing PTSD symptoms (e.g., increased anxiety, rage, increased nightmares, intrusive thoughts, hyperalertness). The CGC noted that clinicians can consider consulting with psychiatric specialists to develop a tapering strategy that minimizes these risks.

#### Management of Sleep Disturbance in Patients with Co-occurring Psychiatric Conditions

Sleep disturbance is a common symptom during BZD tapering and may contribute to symptom exacerbation of underlying mood or psychotic disorders.^2,164,165^ The CGC recommends that clinicians monitor sleep closely in these patients, particularly those with bipolar disorder because sleep disturbance can trigger episodes of mania. If patients with psychiatric conditions experience sleep disturbance, clinicians should pause the taper until symptoms resolve, unless continued BZD use presents imminent safety concerns. In addition to pausing the taper, clinicians can provide patients with information on sleep hygiene and offer or provide them with referrals for alternative treatment options such as CBT-I.^157,166^ Clinicians can also consider consulting with psychiatrists or sleep medicine specialists to help guide treatment plans.

### Older Adults

**Recommendation for Older Adults**

29. Clinicians should generally taper BZD medication in older adults unless there are compelling reasons for continuation (*Clinical Consensus*, Strong Recommendation).

**Implementation Considerations**

- In general, the CGC recommends tapering BZDs in older adults because the risks of continued use tend to be higher in this population. However, clinicians should still base decisions to taper BZD in older adults on a careful assessment of risks and benefits for each individual patient.
- The goal of tapering for older adults may be discontinuation of the BZD or reducing the BZD dose to the point where the risks no longer outweigh the benefits.
- Care should ideally be coordinated between the clinician managing the BZD taper and other clinicians managing conditions that may be impacted by BZD prescribing or the BZD taper.
- An estimated 2 million older adults in the USA have been taking prescribed BZDs for more than 120 days.^9,167^ Many healthcare systems may not be able to manage the volume of older adult patients who would benefit from a BZD taper. As such, clinicians and healthcare systems may need to triage patients, prioritizing those at higher risk of harm related to continued BZD use. See “Implementing this Guideline” for further discussion.

#### Rationale

Although BZDs may offer short-term benefits, the adverse effects associated with their use—including risk of falls and cognitive impairment—have generally been shown to outweigh the marginal benefits in adults 65 years and older.^35^ Chronic BZD use is also a significant concern for older adults given they are likely to be prescribed multiple medications, increasing their risk of morbidity and mortality from polypharmacy.^94,95^ For these reasons, the *AGS Beers Criteria* recommends avoiding the use of both long- and short-acting BZDs in adults over 65 years of age.^168^ Clinicians should generally consider alternative treatment options with more favorable safety profiles.

The CGC recommends that clinicians make every effort to taper BZD use in older adults unless there are compelling reasons for continuation. However, they noted that the decision to taper should still be made based on a careful assessment of risks and benefits for the patient. For example, BZD are sometimes prescribed to control agitation in older adults. The benefits of controlling the patient’s agitation may outweigh the potential adverse effects of the BZD and be a compelling reason for continuing the medication.

Fragmented care can be a barrier to effective BZD tapering because attitudes, knowledge, and conflicting advice from a patient’s medical teams (e.g., primary care, psychiatry, neurology, other specialty clinicians) and care partners can influence the BZD tapering process.^92,169,170^ Although this situation may exist for any patients with multiple healthcare providers, it is particularly common among older adults. Further complicating the matter is that metabolic changes associated with aging make older adults more sensitive to BZDs, increasing their risk of adverse events such as cognitive impairment, particularly in the domains of memory, learning, attention, and visuospatial ability.^92,171,172^ Because older adults are often taking multiple medications from multiple providers, a full medication review and reconciliation should be conducted prior to attempting a BZD taper.

Tapering BZDs in older adults—particularly those with cognitive impairment—can be challenging, especially when patients may lack the capacity to make independent healthcare decisions. Direct educational interventions (e.g., brochures) can help engage older adults—including those with mild cognitive impairment—and their care partners in shared decision-making around BZD tapering and discontinuation.^173^ A patient’s medical teams and care partners are essential in shared decision-making between patients and clinicians regarding BZD tapering methods that consider a patient’s individual needs. If the patient has demonstrated cognitive impairment, they (as well as any care providers) should be provided with clear instructions about the tapering process.

The patient panel noted that in older adults, even lower BZD doses might be associated with significant withdrawal risk due to metabolism changes. They emphasized the importance of starting with smaller dose reductions and proceeding more slowly with tapering in this population. As with all patients, clinicians should prioritize developing individualized tapering plans through shared decision-making.

#### Transitioning Older Adults to a Longer-Acting Benzodiazepine for Tapering

As discussed in “The Tapering Process,” clinicians can consider transitioning patients without contraindications (e.g., liver dysfunction) to a comparable dose of a longer-acting BZD for the taper. However, metabolic changes associated with aging—namely, reduced hepatic clearance—may increase risk of adverse events and toxicity.^171^ As a result, the CGC cautioned against transitioning older adults to longer-acting BZDs prior to tapering.

#### Level of Care Considerations for Older Adults

Older adults, especially those with any degree of cognitive impairment, are at increased risk of poor outcomes in inpatient settings due to hospital-induced delirium and decompensation.^174^ The CGC emphasized that clinicians should attempt to taper BZDs in older adult patients in outpatient settings unless there is a specific indication for inpatient tapering, such as an imminent safety concern that will not be rapidly mitigated by the initial BZD dose reduction. Tapering may need to occur in inpatient or residential settings if outpatient tapering would be unsafe—for example, because family members and the care team cannot manage the older adult in their home environment. In these cases, specialized inpatient units for older adults or skilled nursing facilities are preferred, if available.

### Patients Who Are Pregnant and Lactating

**Recommendations for Patients Who Are Pregnant and Lactating**

28. Clinicians should weigh the risks and benefits for the maternal–fetal dyad when considering continued BZD prescribing or tapering for pregnant patients (*Clinical Consensus*, Strong Recommendation).
29. For infants who have been exposed to BZD in utero and are at risk of neonatal withdrawal symptoms, clinicians should:
  a Encourage breastfeeding, which can reduce neonatal withdrawal symptoms (*Clinical Consensus*, Strong Recommendation)
  b Communicate with the infant’s healthcare provider (with parental consent) regarding exposure to BZDs (*Clinical Consensus*, Strong Recommendation)

**Implementation Considerations**

- Clinicians should monitor patients who are pregnant closely for psychiatric symptoms during the BZD taper, as these symptoms may evolve rapidly during the pregnancy and postpartum period due to shifts in metabolism that influence the effects of medications, including BZDs. Clinicians should address evolving psychiatric symptoms as clinically indicated.
- Care should ideally be coordinated between the clinician managing the BZD taper and the prenatal care provider.
- Clinicians can consider consulting with healthcare professionals who have expertise in reproductive psychiatry or providing patients with referrals to these specialists.

#### Rationale

Although causation remains unclear, BZD use in pregnancy has been found to be associated with an increased risk of miscarriage, preterm birth, and low birth weight.^175–177^ However, antenatal exposure to BZDs is not associated with major congenital malformations.^175,178^ Approximately 20 to 40% of neonates who have been exposed to BZDs in utero during late pregnancy develop neonatal withdrawal, with symptoms including irritability, increased sedation, abnormal muscle tone, poor feeding, sleep problems, and mild respiratory distress.^179–183^ Floppy infant syndrome (FIS)—which presents with hypotonia, lethargy, sucking difficulties, low Apgar score, hypothermia, apnea, cyanosis, hyperbilirubinemia, and CNS depression—has also been observed in newborns who have been exposed to BZDs in utero during the third trimester and may be a result of BZD toxicity.^184,185^ Both neonatal BZD withdrawal and FIS typically present within the first hours of life and continue for up to 14 days.^184^

Although BZD use during pregnancy may carry some risk to the fetus, similar risks are also present if patient anxiety, mood, and sleep disorders are left untreated, including an increase in miscarriage, preterm birth, and low birth weight.^175,186^ In general, existing clinical guidelines recommend optimizing alternative therapeutic approaches (e.g., CBT, CBT-I) and advise caution with BZD dosing during pregnancy.^187^ The CGC suggests that clinicians prescribe BZDs sparingly at the lowest effective dose and with consideration of the pharmacokinetic changes that occur during pregnancy (see Appendix 6). BZD tapering can be done safely in pregnancy^188,189^; however, ACOG has noted that^186^^(1278)^:

[I]t is also critical to consider the risks of a taper for the pregnant individual and the fetus. For example, if attempts to taper the benzodiazepine precipitate re-emergence of anxiety, the benefits of continuation may outweigh the risks.

Due to these considerations, the CGC recommends clinicians discuss the risks and benefits of BZD use and discontinuation for the maternal–fetal dyad with pregnant patients, considering each patient’s unique needs and engaging in shared decision-making to determine whether to taper. Lorazepam is generally preferred in pregnancy and lactation due to its lack of active metabolites and low relative infant dose (RID; i.e., the percent of a patient’s dose ingested by an infant who is exclusively fed with breastmilk). However, if a patient is stable on another BZD, it is not typically necessary to require them to switch. Although, for the reasons outlined in “Transitioning to a Longer-Acting Benzodiazepine,” clinicians should consider transitioning to lorazepam for pregnant patients currently taking alprazolam. Clinicians may consider consulting with specialists in reproductive psychiatry or providing patients with referrals to these specialists, if available.

#### Breastfeeding

In general, breastfeeding is not contraindicated in the presence of BZD use.^190^ The long-term effects of BZD exposure are unknown, but evidence has suggested that the amount of BZD transferred into breast milk is low.^191,192^ Although breastfeeding is unlikely to prevent neonatal abstinence syndrome (NAS), research has suggested breastfeeding can substantially delay the onset and reduce the severity of NAS, decrease the need for pharmacologic treatment, and lead to shorter hospitalization stays compared to formula-fed infants.^193^ Thus, the CGC recommends that clinicians encourage breastfeeding to help reduce potential symptoms of NAS in the infant. Furthermore, breastfeeding has been shown to enhance parental bonding and promote attachment and is associated with a reduced rate of child removal.^194^

## HEALTH DISPARITIES

It is well established that implicit bias can affect how health professionals engage with their patients, diagnose health conditions, determine treatment options, and prescribe medications.^54,195^ Biases, which may be positive or negative, can contribute to disparities in care, including in prescribing and discontinuing medications.^54^ Multiple studies have shown disparities in BZD prescribing.^195–197^ Black, Asian, Hispanic, and multiracial patients are less likely to be prescribed BZDs than White patients.^196,197^ Middle- and lower-income individuals, especially lower-income Black men, are among the least likely to be prescribed BZDs.^195,196^ Additionally, clinicians’ implicit biases may influence responses to prescription policies: BZD prescriptions were most likely to be discontinued for Black patients after prescription monitoring programs went into effect despite lower baseline use.^198^ Taken together, these findings raise concerns that clinician biases can impact decision-making regarding BZD prescribing and discontinuation practices. The CGC encourages clinicians to consider their assumptions and implicit biases and be mindful of how they may impact decision-making as they decide how to implement this Guideline.

## TAPERING WITHOUT PATIENT AGREEMENT

Throughout this Guideline, the CGC has emphasized the importance of clinicians working with patients in a shared decision-making process when considering BZD tapering (see “Partnering with Patients”). However, prescribers may initiate a taper in some instances when patients are ambivalent about or against tapering, including when there are concerns for:

- Patient safety, for example:
  - When patients do not agree to initiate a taper despite collaborative discussions outlining how the risks significantly outweigh the benefits for them
- Community safety, for example:
  - When patients pose a threat to the safety of clinicians, staff, or other patients
  - When patients are diverting their medication
  - When patients engage in criminal behaviors within treatment settings

BZD tapering should not be punitive in these situations. In cases of concerns for patient safety, clinicians should base decisions on careful assessment of the risks and benefits for the patient. In cases of community safety, clinicians should base decisions on assessment of the risks to the patient, clinicians, staff, other patients, and others in the patient’s community.

### Patient Safety

The Patient Panel expressed strong reservations about tapering without a patient’s consent. The CGC understands the Patient Panel’s reservations and encourages clinicians to discuss their concerns that continued BZD use is not in the patient’s best interest with the patient and consider the patient’s concerns and reasons for disagreement. Clinicians should be mindful of any potential bias when initiating a taper against a patient’s wishes. If clinicians and patients and their care partners continue to disagree on the need for a taper after this discussion, clinicians may consider referral for a second opinion.

When initiating a taper without a patient’s consent, clinicians should carefully explain the reasons for their decision to the patient and their care partners, if applicable. Clinicians should also carefully document the rationale for initiating the taper and related discussions. Clinicians should then explain to patients that their next prescription will be at a lower dose and describe how they will monitor and manage patient symptoms and concerns during the tapering process. As emphasized throughout this Guideline, the tapering process should be patient-centered. It is important for clinicians to closely monitor a patient’s response to the taper and adjust the strategy as appropriate.

Some patients may have negative responses to proceeding with tapering without their buy-in. The CGC noted that, in their collective experience, some patients may become aggressive, threaten legal action, or suggest that progressing with the taper may lead them to suicide. The CGC recommends that healthcare systems have established policies and procedures to guide clinicians in their response to these situations in ways that are responsive to a patient’s needs and supports ready access to risk management services.

#### Safety Concerns for Inherited Patients

Clinicians sometimes inherit patients who have been prescribed high-dose and/or long-term BZDs. The same risk–benefit considerations apply when determining whether to continue prescribing BZD medications for these patients (see Table 2). Clinicians can attempt to consult with prior prescribers and other relevant physical and mental health providers. Clinicians should follow the recommendations in this Guideline when assuming responsibility for new patients, including assessing the risks and benefits of continued BZD prescribing and engaging in a shared decision-making process with patients and their care partners. Clinicians should recognize that working with new providers can be stressful for patients. Patients may require extra time to understand the rationale behind a recommendation for tapering and buy into the tapering plan.

If clinicians are not comfortable assuming responsibility for these prescriptions, they can consider referring these patients to another provider or a more intensive level of care, as appropriate, with a bridging prescription to prevent abrupt discontinuation of the BZD medication. However, as discussed in Box 2, it is critical that patients at risk of BZD withdrawal are not abandoned. Alternatively, clinicians may consider initiating a taper without patient agreement (see “Tapering Without Patient Agreement”).

### Community Safety

When continued BZD prescribing jeopardizes community safety, clinicians should explain the reasons for their decision to taper to patients, carefully documenting the rationale and related discussions. Best practices include providing a written summary to patients. If concerns for community safety necessitate discharging patients from their care, clinicians should offer referrals to appropriate alternative providers or treatment services that can manage the patient’s individualized needs during the tapering process, providing warm handoffs as appropriate if patients are amenable. If patients decline the referral, clinicians may consider a BZD tapering plan that accounts for the safety of all parties.

When community safety is a concern, clinicians may need to initiate a more rapid taper than would typically be indicated to balance conflicting obligations. For example, clinicians have a duty to report suspected medication diversion and discontinue prescribing medications if they are being diverted. At the same time, clinicians have a duty to patients who may be at risk of life-threatening withdrawal if medications are discontinued abruptly. Clinicians should consider seeking the advice of legal counsel, risk management, and health systems administrators in these complex situations. State licensing boards, professional organizations, and clinician malpractice insurance organizations may also have guidance available.

Clinicians may consider implementing a discharge taper to prevent severe or complicated withdrawal—for example, providing patients with a 14-to- 30-day prescription with detailed instructions on how to taper the medication over that time period. Weekly prescriptions can be considered to reduce the risk of misuse, but they may not always be feasible for the prescriber or the patient due to appointment availability, safety concerns, cost, or transportation barriers. When determining the dose and number of pills for a discharge taper, clinicians should carefully consider an individual patient’s risks, including suicidality and overdose. Given uncertainties regarding patient follow-up after discharge, clinicians may also consider offering patients prescriptions for adjunctive medications to help alleviate potential withdrawal symptoms (see Appendix 5). Clinicians should clearly communicate to patients that this will be the last BZD prescription provided, as well as the risks of abrupt discontinuation of BZD and the symptoms that should trigger patients to seek emergency medical care. Clinicians should document this encounter carefully.

Some patients may be upset at the prospect of BZD tapering. Clinicians should be aware of the potential for this response and consider how to mitigate risks to themselves, their staff, and other patients. De-escalation strategies may help reduce patients’ anger and frustration. Other strategies clinicians can consider include positioning themselves close to the door, having another staff person in the room, conducting the appointment via telemedicine, and alerting clinic security in advance, if available. Clinics that experience these types of challenges more often can consider implementing help buttons in appointment rooms that allow clinicians to silently alert other staff of their need for assistance. Clinicians can also develop a code word or phrase to subtly warn staff of dangerous situations and prompt them to summon clinic security for help.

These situations are challenging for clinicians, staff, and patients. Clinicians should consider consulting with their organization’s legal and/or risk management teams and their malpractice carrier if they have concerns. Furthermore, the CGC recommended that organizations have policies and procedures in place to support clinicians and staff in situations where a patient’s preferences are not congruent with safe medical prescribing. Clinicians and staff should also be cognizant of their own mental wellness when dealing with difficult patient encounters and be able to pursue support without fear of repercussions.

## CONSIDERATIONS FOR EMERGENCY DEPARTMENTS

EDs have unique considerations for BZD tapering as they are subject to the Emergency Medical Treatment and Active Labor Act (EMTALA), which requires them to provide any individual who comes to the hospital with necessary stabilizing treatment for emergency medical conditions. Clinicians should not routinely refer patients to EDs unless they are experiencing or imminently expected to experience severe acute withdrawal. However, ED clinicians may commonly encounter patients who:

- Are withdrawing from nonprescribed BZDs
- Are not tolerating a BZD taper from their regular prescriber
- Have lost access to their BZD prescription (e.g., discontinued by their regular prescriber)

When applicable, ED clinicians should attempt to coordinate with patients’ regular prescribers. However, the CGC recognized this is often not possible in a reasonable time frame. Clinicians should screen patients who are experiencing withdrawal from nonprescribed BZD for SUD and consider referring them to an appropriate level of specialty care for SUD (see “Patients with Benzodiazepine and Other Substance Use Disorders”).

Due to the lack of capacity for direct follow-up, ED clinicians are not well positioned to provide ongoing management of BZD tapering. However, ED clinicians can consider:

- Providing patients with a bridging BZD prescription at the same or slightly lower BZD dose, as appropriate, with referral to outpatient providers as needed
- Initiating a short taper as discussed in “Community Safety”
- Initiating a taper using a very long-acting agent (e.g., phenobarbital) as discussed in “Tapering with Very Long-Acting Agents” and referring patients to appropriate providers for ongoing care needs

The specific strategies used depend on a patient’s presentation and available resources. However, if continued BZD prescribing presents safety concerns, a clear plan for safe tapering and follow-up should be in place at the time of discharge from the ED. If available, clinicians should consider engaging social workers, patient navigators, or peer recovery specialists to support this process.

## STRATEGIES FOR PREVENTING DIVERSION

Continued prescribing when clinicians are aware patients are diverting controlled medication creates legal risk of them. In addition, clinicians’ DEA registration and license to practice could be in jeopardy. This can lead to complex situations wherein prescribers have to balance these legal and professional risks against the risks associated with rapid BZD discontinuation for patients. Prescribers should educate patients on the consequences of medication diversion, including required reporting and medication discontinuation, in a patient-centered manner. Prescribers who are concerned about the potential for diversion can consider:

- Screening for and addressing substance misuse and use disorders
- Implementing pill checks
- Implementing medication agreements with patients
- Writing prescriptions with shorter durations
- Limiting prescription refills
- Partnering with collateral contacts (e.g., family members, friends, care partners)
- Coordinating with pharmacies
- Checking the PDMP when initiating or refilling prescriptions
- Conducting periodic confirmatory drug testing for the prescribed BZD

Prescribers can include a note to pharmacists in e-prescriptions requesting that pharmacists only fill BZD prescriptions from their office. Integrated care systems may consider including pharmacists on treatment teams. Some payers, including Medicaid, can implement controlled substance agreements to restrict who is allowed to prescribe controlled substances for a given patient. Controlled substance agreements can specify that patients can only fill prescriptions for controlled substances at a specific pharmacy. Prescribers can also work with payers to request case managers who can conduct drug utilization reviews, which allows prescribers to see all of a patient’s medications, not just those in the PDMP.

## IMPLEMENTING THIS GUIDELINE

As clinicians and healthcare systems implement this Guideline, they may identify a large population of patients who would benefit from tapering. The recommendation to taper BZD in most older adults “unless there are compelling reasons for continuation” has implications for the estimated 2 million older adults in the USA who have been using BZDs for more than 120 days.^9,167^

The CGC recognizes healthcare systems are already overburdened, and significant workforce challenges may limit the capacity to manage BZD tapering at scale. This may be particularly evident in primary care settings, which are responsible for the majority of BZD prescriptions in the USA.^19^

As emphasized throughout this Guideline, BZD tapering requires close monitoring and can be clinically complex. Although tapering may be a relatively simple process for some patients, others will experience significant challenges and require closer management. Clinicians and healthcare systems may need to develop strategies for prioritizing those patients who are at the highest risk in the short term. For example, patients who have recently experienced adverse events related to BZDs may be prioritized for tapering over those who have not.

It will be important for healthcare systems and policymakers to consider how to best leverage existing healthcare resources to meet the needs of the population. Models for scaling dissemination of healthcare best practices, such as hub and spoke models and Project ECHO (Extension for Community Healthcare Outcomes), may help address these challenges.^199^ Telemedicine may also help extend the existing workforce’s capacity. However, telehealth-based interventions will not be appropriate or accessible for all patients; clinicians should determine its appropriateness for a given patient.

Healthcare providers should also be cautious in how they measure and evaluate success. Focusing on reductions in BZD prescribing may lead healthcare systems to ignore important patient outcomes. Evaluations should consider patient experiences as well as adverse events associated with the tapering process. How many patients experience significant protracted withdrawal symptoms? How long do these symptoms last? What are the impacts of BZD tapering on patient quality of life, functionality, physical health outcomes, and mental health outcomes, including suicidality? If BZD tapers are managed poorly there is a real risk of patient harm. Efforts to reduce BZD prescribing must remain focused on improving patient outcomes, considering the whole of their experiences.

### Expert Consultation

Some patients will experience more challenges with tapering than others and would benefit from expert consultation. Specialists in addiction medicine, addiction psychiatry, and medical toxicology have the requisite expertise. For older adults, geriatric psychiatry or geriatric medicine specialists may be appropriate. However, workforce shortages limit access to these specialists in many areas of the country.

### Clinician Education

Patients have reported difficulty finding knowledgeable providers for BZD tapering.^128,200^ Patients have also reported that their withdrawal symptoms were often ignored, misattributed to recurrence of the conditions for which the BZD was initially prescribed, or misdiagnosed as another condition.^128,200^

Clinician training is needed on the appropriate use of BZDs, their adverse effects, risks of dependence, withdrawal symptoms, tapering methods, and protracted withdrawal. Education on BZD prescribing and tapering, with monthly feedback on their BZD prescribing rate compared to other local clinicians, has been shown to lead to a reduction in BZD prescriptions and fewer patients taking BZDs long-term.^201^

### Technological Innovations

Existing technologies can help support implementation of this Guideline. Automated or smart pill dispensers may support adherence among patients who have memory or cognitive concerns or are at risk of medication misuse.^202,203^ In addition, multiple mobile applications support tracking and management of symptoms common during BZD tapering (e.g., sleep impairment, anxiety, depression).^204–208^ Clinicians can consider whether these technologies may support an individual patient’s needs.

## FINAL THOUGHTS

Many of the topics discussed in this Guideline lacked controlled studies. Our systematic review found no trials comparing BZD tapering strategies or other important aspects of managing patients who are taking prescribed BZDs and likely to have developed physical dependence. Further research into best practices for BZD tapering strategies that support patient safety and optimal outcomes is urgently needed.

## Supplementary Information

Below is the link to the electronic supplementary material.

Supplementary file1 (DOCX 96.6 KB)

Supplementary file2 (DOCX 760 KB)
