# Supplementary material for: Joint Clinical Practice Guideline on Benzodiazepine Tapering: Considerations When Risks Outweigh Benefits
Source: J Gen Intern Med. 2025 Jun 17;40(12):2814–59. doi: 10.1007/s11606-025-09499-2 (PMC12463801; doi:10.1007/s11606-025-09499-2)
Supplement: Supplementary file 1 — Supplementary file1 (DOCX 96.6 KB) [file 11606_2025_9499_MOESM1_ESM.docx]

# Appendix 1. Pharmacokinetic Properties of Benzodiazepines

Table 5 summarizes pharmacokinetic properties of BZD that are important to consider in determining relative risk of physical withdrawal and planning tapering strategies.

**Table 5 Pharmacokinetic Properties of Benzodiazepines^209^**

| **Benzodiazepine** | **Time to peak plasma level (h; via oral)** | **Relative lipid solubility*** | **Onset of action (min)^†^** | **Elimination half-life (h)^‡^** | **Metabolism^§^** |
| --- | --- | --- | --- | --- | --- |
| Alprazolam | 1–2 h (tablet or ODT)  5–11 h XR | Moderate | 15–30 | 6–12 | CYP3A4 |
| Chlordiazepoxide | 0.5–4 h | Moderate | 15–30 | 5–10  36–200 (AM) | CYP3A4 |
| Clonazepam | 1–2 h | Low | 15–30 | 18–50 | CYP3A4 |
| Clorazepate^‖^ | 0.5–2 h | High | 15 |  | CYP2C19  CYP3A4 |
| Diazepam | 0.5–2 h | High | ≤ 15 | 20–100  36–200 (AM) | CYP1A2  CYP2C9  CYP2C19  CYP3A4 |
| Estazolam | 2 h | Low | 30–60 | 10–24 | CYP3A4 |
| Flurazepam | 0.5–2 h | High | ≤ 15 | 40–250 (AM) | CYP2C19  CYP3A4 |
| Lorazepam | 2–4 h | Moderate | 15–30 | 10–20 | Glucuronide conjugation |
| Oxazepam | 2–4 h | Low | 30–60 | 4–15 | Glucuronide conjugation |
| Quazepam^210^ | 2 h | High | 15 | 39  73 (AM) | CYP2C9  CYP2C19  CYP3A4 |
| Temazepam | 2–3 h | Moderate | 30–60 | 10–20 | Glucuronide conjugation |
| Triazolam | 1–2 h | Moderate | 15–30 | 1.5–5 | CYP3A4 |

This table outlines pharmacokinetic properties of various BZD medications, including the time to peak plasma level following oral administration, relative lipid solubility, onset of action, elimination half-life of the active metabolite, and metabolism

*AM* active metabolite, *ODT* orally disintegrating tablet, *XR* extended release

*Increased lipid solubility results in more rapid onset of CNS activity but can also result in rapid redistribution into adipose tissue resulting in a shorter duration of action even in agents with long elimination half-life (e.g., diazepam)

^†^Rapid onset of action is associated with high lipid solubility and increased potential for misuse

^‡^Agents with moderate to high lipid solubility will have shorter duration of action with single or intermittent doses than suggested by the elimination half-life as these medications distribute rapidly into adipose tissue. With initial dosing, multiple daily doses may be needed to maintain effect. With chronic use and repeated dosing, accumulation is more likely to occur with these agents, especially those with long elimination half-lives (e.g., diazepam)^211^

^§^Agents metabolized via glucuronide conjugation do not have pharmacokinetic interactions and are considered to be safer in older adults and patients with hepatic impairment

^‖^Hydrolyzed to nordiazepam in the stomach

# Appendix 2. Benzodiazepine Dose Equivalents

Table 6 summarizes approximate dose equivalents to inform decisions about transitioning from one BZD to another for the purpose of tapering.

**Table 6 Benzodiazepine Approximate Dose Equivalents to 10 mg Oral Diazepam**

| **Benzodiazepine** | **ATC therapeutic class** | **VA/DoD SUD CPG (2021)^212^** | **Ashton manual (2002)^213^*** |
| --- | --- | --- | --- |
| Alprazolam | Anxiolytic | 1 | 0.5 |
| Chlordiazepoxide | Anxiolytic | 25 | 25 |
| Clonazepam | Antiepileptic | 1 | 0.5 |
| Clorazepate | Anxiolytic | 15 | 15 |
| Diazepam | Anxiolytic | 10 | 10 |
| Estazolam | Sedative–hypnotic | 1 | 1–2 |
| Flurazepam | Sedative–hypnotic | 15 | 15–30 |
| Lorazepam | Anxiolytic | 2 | 1 |
| Oxazepam | Anxiolytic | 30 | 20 |
| Quazepam | Sedative–hypnotic | 10 | 20 |
| Temazepam | Sedative–hypnotic | 15 | 20 |
| Triazolam | Sedative–hypnotic | 0.25 | 0.5 |

Approximate dose equivalents of various BZD medications to a 10 mg dose of oral diazepam as determined by the VA/DoD SUD guideline and *The Ashton Manual*. The doses are intended for guidance only. Clinical decisions on dose should be individualized based on the patient response

*ATC* Anatomical Therapeutic Chemical classification system, *CPG* clinical practice guideline, *DoD* US Department of Defense, *SUD* substance use disorder, *VA* US Department of Veterans Affairs

*Same equivalents in Ashton^214,215^

**Appendix 3. Sample Tapering Strategies and Case Descriptions**

## Tapering Case Descriptions

This Appendix contains five case descriptions highlighting a variety of aspects of BZD tapering, including patient engagement, considerations for tapering, tapering strategies, withdrawal management, and population considerations. These cases are not meant to endorse specific tapering strategies or protocols but are meant to illustrate how the recommendations in this Guideline may be applied to a variety of clinical scenarios.

### Case 1: Taking Over a Long-Term BZD Prescription in Primary Care

Mr. Z is a 59-year-old male who has been taking 2 mg clonazepam twice daily for an unknown number of years. He was started on the medication “years ago” during a period of high stress when he had lost his job and gotten divorced. You have an established relationship with Mr. Z as his primary care physician treating him for hypertension and diabetes. Mr. Z’s psychiatrist recently retired, leaving you to manage his psychiatric medication.

You engage Mr. Z in a discussion of his BZD medication. You express concern that his dose is fairly high, especially considering his other medical conditions. He objects at first, stating that his psychiatrist never saw a problem with the amount of medication he was taking. You educate Mr. Z on the risks of continued use and share that he may feel better taking less medication. However, he is afraid to stop taking the medication because he experienced intolerable anxiety when he missed a dose once. You educate Mr. Z on withdrawal symptoms, explaining the symptoms he experienced when missing a dose may have been related to withdrawal. You explain to Mr. Z that he will likely experience some withdrawal symptoms, but you will work with him to minimize these and make them tolerable. Mr. Z agrees to try tapering.

Prior to beginning the taper, you help Mr. Z locate a therapist to help with stress management. You and Mr. Z agree that a small reduction of his total daily clonazepam dose from 4 to 3.75 mg (i.e., a 6.25% reduction) would be the best place to start, given the symptoms he experienced with missing an entire dose previously. You instruct Mr. Z to finish his remaining 1-week supply of 2 mg tablets. You then prescribe him 0.5 mg tablets, such that his dose becomes 3.5 tablets in the morning and 4 tablets at bedtime for a total daily dose of 3.75 mg. Mr. Z reports some sleep difficulty and anxiety, so the 3.75 mg daily dose is maintained for 2 months.

After 2 months, you and Mr. Z agree to decrease his dose to three 0.5 mg tablets in the morning and four tablets in the evening, bringing his daily dose down to 3.5 mg (a 6.67% decrease from the prior dose). After 1 month, Mr. Z is ready for the next decrease in dose to three 0.5 mg tablets in the morning and three and a half 0.5 mg tablets at bedtime for a total daily dose of 3.25 mg (7.14% reduction from prior dose). Further dose decreases included 0.25 mg reductions every 4–6 weeks beginning with the morning dose followed by the evening dose, until Mr. Z reached a total 2.5 mg dose. At that point, Mr. Z begins to experience increased anxiety. You and Mr. Z agree to pause the taper for 10 weeks.

After 10 weeks, you and Mr. Z agree to continue with smaller dose reductions, reducing the dose by 0.125 mg. You prescribe 0.25 mg tablets and instruct Mr. Z to take four and a half in the morning and five at bedtime for a total daily dose of 2.375 mg. You continue reducing the daily dose by 0.125 mg every 4–6 weeks, based on shared decision-making and symptom tolerability. Although it takes nearly 2 years, Mr. Z is able to completely stop his BZD.

### Case 2: Challenging Alprazolam Taper in Primary Care

Ms. D is a 36-year-old female who has been taking 0.5 mg alprazolam 3 times per day for 3 years. She was initially prescribed alprazolam for anxiety with panic attacks, but reports it is also helpful for her irritable bowel syndrome, migraines, and menstrual cramps. She had not tried other medication classes or therapy before starting alprazolam. Ms. D previously received medication from her gynecologist and gastroenterologist at separate times, and she is now transitioning care to you as primary care physician. Ms. D is requesting an increase in her dose because she is experiencing an increase in anxiety.

Given the potential harms associated with BZDs, current guidelines are that they should be reserved for treatment-resistant cases of anxiety disorders where other treatment options have failed. For Ms. D, it would be best to try some other strategies with fewer associated risks to see if they might be effective. You engage Ms. D in a discussion of the evidence-based treatment options for her medical conditions, and share that BZD are not first-line treatments for these conditions. You educate Ms. D about the risks associated with ongoing use of BZD, and you assure her there are other pharmacological and nonpharmacological treatments that can be helpful. You reassure Ms. D that you are committed to finding an approach that will treat her symptoms, but that this process may take time. Ms. D is amenable to trying an SSRI and CBT and to tapering from her alprazolam once the SSRI has been titrated to an effective dose for her.

You start Ms. D on sertraline to address symptoms of anxiety as well as IBS and migraines. In parallel, you locate a CBT treatment provider and facilitate the referral. When the sertraline begins to show clinical effect, Ms. D begins the tapering process. Due to the potential difficulty in tapering from alprazolam given its short half-life and lack of active metabolites, you begin by slowly switching Ms. D to an equivalent dose of diazepam, explaining that a longer-acting medication may be easier to taper. Ms. D agrees that twice daily dosing would be more convenient and you begin the transition to 7.5 mg ([one and a half 5 mg tablets] 2×/day, for a total daily dose of 15 mg), switching one dose at a time over the course of 2 weeks. She reports experiencing significant withdrawal symptoms at this dose and you adjust to a 17.5 mg daily dose (two 5 mg tablets in the morning and one and half tablets in the evening).

After she has acclimated to the new medication, you begin tapering. Ms. D opts to begin with a reduction of her morning dose by 2.5 mg (for a total daily dose of 15 mg, a 14.3% reduction). Ms. D continues making 2.5 mg dose reductions every 4 weeks until she reaches an overall daily dose of 5 mg. At this point, Ms. D begins to experience increased withdrawal symptoms, so you pause the taper and maintain her on the current dose for 8 weeks. At this point, she is ready to continue with a slower pace of taper. Ms. D agrees to a 1 mg dose reduction so you prescribe 2 mg tablets. She begins taking one 2 mg tablet in the morning and one in the evening for a total of 4 mg daily, but reports a significant increase in withdrawal symptoms. You again pause the taper; however, she continues to report intolerable symptoms. You agree to try a 4.5 mg daily dose (one and one-fourth pills in the morning and one in the evening). At this dose, her symptoms are more manageable, but she is very nervous about continuing to taper. You agree to pause at the current dose for 8 weeks and then try an even slower taper.

At 8 weeks, Ms. D indicates that she has just taken on new caregiving responsibilities for her father who is receiving cancer treatment and is experiencing a lot of stress as a result. She asks to delay the taper and you agree, with a plan to revisit the plan in another 8 weeks. You recommend that she can try skipping the one-fourth tablet on days when it feels manageable. At her next visit, she notes that she skipped the one-fourth tablet a few times and it went okay. She agrees to try the 4 mg daily dose again.

After 4 weeks, she reports that she is very nervous about continuing the taper but that she is willing to try. You reassure her that she can reach out at any time if the symptoms are intolerable and you will come up with a plan together. She agrees to a 3.5 mg dose (one 2 mg tablet in the morning and three-fourths of a pill in the evening). Ms. D begins to experience intolerable insomnia. She calls your office to say she does not think she can handle this right now with all of the stress she is under. You discuss adding an adjunctive medication, such as mirtazapine or trazadone, to help her sleep. She is hesitant to add another psychiatric medication. After further discussion, she reveals that her sleep problems relate to nightmares that interrupt her sleep and prevent her from falling back to sleep. You note that nightmares are commonly associated with PTSD and recommend that she get assessed for this, noting the effective treatments are available, including eye movement desensitization and reprocessing (EMDR). You also discuss adding prazosin, a medication that is commonly used to treat high blood pressure but can also be used to treat PTSD associated nightmares. She agrees to try it.

You ask Ms. D if she thinks she can tolerate the current dose of BZD if you plan to maintain it while she begins prazosin and schedules an assessment for PTSD with her therapist. She expresses confidence in this plan. After a few weeks on prazosin, she is ready to continue with BZD dose reductions. She agrees to a 3 mg dose (three-fourths of a 2 mg tablet in the morning and three-fourths of a tablet in the evening). Ms. D tolerates this dose reduction and after 4 weeks agrees to a 0.5 mg dose reduction, beginning with the morning dose (one-half of a 2 mg tablet in the morning and three-fourths of a tablet in the evening, for a 2.5 mg total daily dose). Ms. D is able to tolerate two more 0.5 mg dose reductions, However, when she reaches a 2 mg total daily dose (one-half of a 2 mg tablet in the morning and one-half of a tablet in the evening), her father passes away. Her grief is overwhelming, and she expresses significant anxiety about continuing BZD dose reductions. You agree to maintain the current dose of BZD, noting that her risks are greatly reduced compared to when she started because of the lower dose. You plan to check in on how she is doing at her next medication refill appointment.

### Case 3: Managing a Long-Term Prescription for an Older Adult

Mr. M is a 75-year-old male who was prescribed lorazepam 2 mg at bedtime PRN for insomnia. He does not recall when he was first prescribed the medication, but he remembers that his dose was increased a few years ago when he was having more trouble sleeping after the loss of his brother. He lives at home with his wife. Electronic records indicate that the patient is filling the PRN prescription regularly, and Mr. M confirmed he is taking the medication daily.

Mr. M denies excessive daytime sedation. However, Mr. M’s wife is concerned that his memory is declining, and at times, he seems confused and disorganized. You engage Mr. M in a conversation about the relationship of BZD with cognitive impairment. Mr. M admits that he feels “foggy” sometimes, but that he did not realize his medication could be a contributing factor to this. He confirms that he is willing to try tapering the BZD but worries that he will not be able to sleep. You share with Mr. M that BZDs are not intended to be used long-term for sleep. You reassure Mr. M that there are other strategies that might even help him sleep better. Unfortunately, you are unable to locate any providers who specialize in CBT-I; however, you show him a mobile CBT-I app that is recommended by the Veterans Administration and you provide education on sleep hygiene strategies. You also provide education on withdrawal symptoms that he might experience, and you encourage Mr. M to let you know right away if these symptoms are intolerable. The goal is to reduce the overall dose down to a safer level and hopefully improve cognition.

Mr. M agrees to reduce his dose by 0.5 mg for 1 week by quartering tablets and taking three-fourths of a tablet at bedtime. After 1 week, Mr. M reports a few bothersome withdrawal symptoms and says he does not feel ready to reduce the dose any further. While avoiding returning to the previous dose, you hold the current dose until Mr. M is ready to reduce his dose further. Within 2 weeks, he reports fewer symptoms and agrees to try another reduction, this time reducing to one-half tablet (dose = 1 mg) at bedtime. After 1 month, Mr. M’s wife reports that his memory seems to be improving. When he is due for a prescription refill, you prescribe 0.5 mg tablets to allow for more dose flexibility. You jointly agree to reduce his dose by 0.25 mg, instructing him to take one and a half 0.5 mg tablets at bedtime (dose = 0.75 mg). After 6 weeks, Mr. M is ready to reduce his dose down to 0.5 mg at bedtime (one 0.5 mg tablet). Toward the end of the taper, you slow the pace (reducing by one-fourth tablet or 0.125 mg every 4–6 weeks) until Mr. M is ready to start skipping doses, and after a year, Mr. M is able to discontinue the medication.

### Case 4: Managing Anxiety in a Pregnant Patient

Ms. L is a 32-year-old female who has been taking 10 mg diazepam 2×/day for anxiety for about 2 months. She just discovered she is 8 weeks pregnant and expresses a strong desire to taper from her BZD for the health of her baby, although she is also concerned about how she will manage her anxiety during pregnancy.

You engage Ms. L in a discussion about the risks and benefits of continuing her BZD, as well as alternative treatment options. You reassure her of treatment options to address anxiety that are safe for her baby, including SSRI/SNRI. While educating Ms. L on SSRI/SNRI, you explain that while these medications can cause neonatal withdrawal symptoms, these are generally less severe and shorter duration compared to BZD-related neonatal withdrawal. You also provide education on withdrawal symptoms and encourage her to let you know if they become intolerable. Ms. L expresses high motivation to try SSRI medication and virtual therapy sessions with a mental health provider, and taper from her BZD. You locate a referral for a therapist skilled in CBT and prescribe a course of sertraline.

At 10 weeks, Ms. L initially reduces her morning dose to 7.5 mg (one and a half 5 mg tablets) and continues to reduce her dose every 3 weeks through the second trimester. At 24 weeks, after switching to 2 mg tablets, she has tapered down to 3 mg and reports increased withdrawal symptoms. You adjust the tapering process to smaller and less frequent dose reductions, and by 34 weeks, she has tapered from the BZD medication completely. Ms. L delivers a healthy baby. You continue to follow Ms. L closely to monitor for postpartum anxiety.

### Case 5: Managing Risk of Severe Withdrawal in a Patient with Benzodiazepine Use Disorder

Mr. B is a 22-year-old male, who started using alprazolam he obtained from friends to “deal with stress.” Mr. B then began purchasing BZD pills from websites. He has been taking BZD for about 3 years and also drinking alcohol in combination with the BZD. He has a history of a seizure in the context of prior alcohol withdrawal. Mr. B presents to a withdrawal management service in an ASAM Criteria Level 3.7 residential addiction treatment facility, requesting help with tapering because he has tried stopping and is unable to do so on his own. He reports that he does not have a PCP.

Mr. B meets criteria for a severe BZD use disorder. Because of his current estimated dose of alprazolam (5–7.5 mg daily) and history of seizure, Mr. B is at risk of severe withdrawal. You would not consider outpatient treatment for this patient due to safety concerns. You admit this patient to the residential withdrawal management unit to begin phenobarbital taper (see “[Sample Residential (Level 3.7) Protocol for Phenobarbital Taper](#_Sample_Residential_(Level)”).

However, once admitted, you conducted a drug screen that is positive for opioids. You suspect Mr. B has been taking counterfeit alprazolam that are contaminated with opioids (including fentanyl), and it is apparent he is also experiencing opioid withdrawal. The patient is transferred to the hospital as management of BZD and opioid withdrawal concurrently is likely to be more complex. Buprenorphine is initiated in the hospital along with a phenobarbital taper (see “[Sample Hospital (Level 4) Protocol for Phenobarbital Taper](#_Sample_Hospital_(Level)”).

During discharge planning, Mr. B is offered ongoing care for SUD, and treatment options are discussed. Mr. B states he prefers to begin a residential treatment program, as his partner is continuing to use substances, and is referred to a local program for SUD treatment and management.

## Sample Residential (Level 3.7) Protocol for Phenobarbital Taper

Disclaimer: This is an example protocol that is currently used in a residential SUD treatment setting for tapering with phenobarbital. It should not be interpreted as an exact recommended protocol.

Clinicians should note the following global considerations for implementing a phenobarbital taper in residential settings:

- Do not start phenobarbital until at least 8 h after the patient’s last BZD use.
  - Patients who primarily use alprazolam may have significant withdrawal symptoms less than 8 h since their last dose. If patients have significant objective signs and symptoms of withdrawal, a phenobarbital protocol can be initiated prior to 8 h.
- Consider a patient’s risk of seizure and manage as appropriate.
- If patients show signs of oversedation, delay the following phenobarbital dose.
- Although the protocol is only 6 days, phenobarbital’s long half-life ensures the medication will continue to be active for several days afterward, resulting in an auto-taper.

During the first day, patients must be assessed at least every 4 h for safety, even if this involves waking them up. Although in rare cases, patients may receive a phenobarbital loading dose intramuscularly or intravenously, the doses in the protocol described in Table 7 are oral.

**Table 7. Sample Residential (Level 3.7) Protocol for Phenobarbital Taper**

| Day 1 | - 64.8 mg initial dose and then 32.4 mg every 4 h - If the patient is experiencing significant withdrawal symptoms, clinicians may add a 32.4 mg dose - 226.8 mg total scheduled daily dose, with a maximum total daily dose of 330 mg |
| --- | --- |
| Day 2 | - 32.4 mg every 4 h - If the patient is experiencing significant withdrawal symptoms, clinicians may add a 32.4 mg dose - 194.4 mg total scheduled daily dose, with a maximum total daily dose of 300 mg |
| Day 3 | - 32.4 mg every 6 h - If the patient is experiencing significant withdrawal symptoms, clinicians may add a 32.4 mg dose - 129.6 mg total scheduled daily dose, with a maximum total daily dose of 240 mg |
| Day 4 | - 32.4 mg every 8 h - If the patient is experiencing significant withdrawal symptoms, clinicians may add a 32.4 mg dose - 97.2 mg total scheduled daily dose, with a maximum total daily dose of 180 mg |
| Day 5 | - 32.4 mg every 12 h - If the patient is experiencing significant withdrawal symptoms, clinicians may add a 32.4 mg dose - 64.8 mg total scheduled daily dose, with a maximum total daily dose of 150 mg |
| Day 6 | The patient may be discharged (or, for patients with SUD, transitioned to a less intensive level of addiction care) when the total daily dose is < 60 mg within 24 h |

An example of a 6-day tapering protocol using oral phenobarbital in a medically managed residential setting

**Sample Hospital (Level 4) Protocol for Phenobarbital Taper**

Disclaimer: This is an example protocol that is currently used in a hospital setting for tapering with phenobarbital. It should not be interpreted as an exact recommended protocol.

- Administer a test dose of 64.8 mg oral phenobarbital.
- Assess patients 1 h after administering the test dose to ensure they are not oversedated or intoxicated.

If patients tolerates the test dose, continue with the following phenobarbital taper schedule:

- 129.6 mg oral phenobarbital every 4 h for a daily total of 6 doses
- 129.6 mg oral phenobarbital every 6 h for a daily total of 4 doses
- 129.6 mg oral phenobarbital every 8 h for a daily total of 3 doses

If patients exhibit any signs or symptoms of oversedation or intoxication, hold the next scheduled dose.

After 72 h, patients should be safe for discharge (or, for patients with SUD, transitioned to a less intensive level of addiction care) without additional phenobarbital or BZD doses.

# Appendix 4. Adjunctive Psychosocial Interventions

Table 8 was created to support [Recommendation 10](#_Recommendations_for_Adjunctive):

Clinicians should offer patients undergoing BZD tapering behavioral interventions tailored to their underlying conditions (e.g., CBT, CBT-I) or provide them with referrals to access these interventions (***Low Certainty***, Strong Recommendation). Table 8 is not intended to be an exhaustive list, but rather a sample of interventions identified by the CGC that may be considered.

**Table 8 Adjunctive Psychosocial Interventions**

|  | **Brief description** | **Papers/resources** |
| --- | --- | --- |
| **Behavioral interventions** | | |
| CBT^216–222^ | Cognitive behavioral therapy is a structured psychological treatment that helps to change thoughts, feelings, and behaviors, to treat a variety of problems | CBT for panic (Otto et al.^216^; Otto et al.^217^; Spiegel et al.^218^)  CBT for BZD withdrawal (O’Connor et al.^219^; Oude Voshaar et al.^220^)  CBT for GAD (Gosselin et al.^222^)  Digital CBT (Klein et al.^222^) |
| CBT-I^223–225^ | Cognitive behavioral therapy for insomnia is a structured psychological treatment that helps to change thoughts, feelings, and behaviors that are contributing to insomnia | Coteur et al.^223^; Morin et al.^224^; Baillargeon et al.^225^ |
| Behavior modification^226^ | Behavior modification is a psychotherapeutic intervention used to eliminate or reduce unwanted behavior | Pottie et al.^226^ |
| Mental health counseling | A variety of psychotherapy approaches are used in practice. Although CBT and behavior modification have the most evidence as adjunctive interventions for BZD withdrawal, other methods may be as or even more effective for specific patients. In general, any mental health provider who is comfortable addressing the reason for a patient’s initial BZD prescription and managing symptoms that may develop during the withdrawal process (e.g., anxiety, insomnia) will likely be helpful for patients | American Counseling Association  National Association of Social Workers  National Alliance on Mental Illness |
| **Lifestyle factors** | | |
| Sleep hygiene^223,227^ | Sleep hygiene refers to environment and behaviors that are conducive to optimizing restorative sleep. These may include avoiding caffeine, stimulants, alcohol near bedtime. Along with setting up a night routine and sleep schedule that is conducive to good sleep | Lähteenmäki et al.^227^; Coteur et al.^223^ |
| Exercise and physical activity^228,229^ | Gentle exercise (e.g., walking or swimming) may be helpful. *The Ashton Manual* recommends regular moderate enjoyable exercise during a benzodiazepine taper | Reconnexion. *The Benzodiazepine Toolkit* (2018:54)  *The Ashton Manual* (2002) |
| Diet^228,229^ | Staying well hydrated, eating a well-balanced diet, and eliminating caffeine (including energy drinks) and alcohol may be helpful | Reconnexion. *The Benzodiazepine Toolkit* (2018:53)  *The Ashton Manual* (2002) |
| Mindfulness^230^ | Mindfulness is a cognitive skill, usually developed through meditation | Barros et al.^230^ |
| **Complementary health approaches** | | |
| Acupuncture^231^ | Yeung described acupuncture as the insertion of “fine needles at special acupoints on the body according to the traditional Chinese meridian theory. The inserted acupuncture needles can be connected by an electric-stimulator to deliver electric-stimulation and is termed as electroacupuncture”^231^ | Electroacupuncture (Yeung et al.^231^) |
| Progressive muscle relaxation^216^ | Progressive muscle relaxation involves alternately tensing then relaxing muscles, one by one | Otto et al.^216^ |
| Anxiety management training (AMT)^232^ | In AMT, patients are “asked to imagine unpleasant events which they had experience, concentrate on early signs of distress and counteract them with relaxation”^232^ | Elsesser et al.^232^ |
| **Peer specialist services** | | |
| Peer support^233,234^ | Individuals who typically have lived experience in BZD tapering, mental health, and/or substance use provide support one-on-one or in group settings, either in-person or virtually, to support people going through BZD tapering | National Institutes for Health and Care Excellence (2022)  Lynch et al.^234^ |

This table presents some common psychosocial interventions that can be considered for use as adjuncts to support management of withdrawal symptoms during BZD tapering

# Appendix 5. Adjunctive Pharmacological Interventions

Tables 9 and 10 were created to support [Recommendation 11](#_Recommendations_for_Adjunctive): Clinicians should first consider pausing or slowing the pace of the BZD taper when patients experience symptoms that significantly interfere with the taper (e.g., sleep difficulty, anxiety), although clinicians can also consider use of adjunctive medications (Clinical Consensus, Conditional Recommendation). These tables provide illustrative examples that may be considered based on the experience of the CGC and are not intended to represent an exhaustive list.

**Table 9 Medications for Anxiety-Related Symptoms**

| **Medication** | **Class/mechanism** | **Considerations for use** | **Other population considerations** |
| --- | --- | --- | --- |
| **Acute anxiety** | | | |
| Clonidine* | Central alpha-2 agonist | Monitor blood pressure; avoid in hypotension  If used as a scheduled medication, taper to discontinue |  |
| Gabapentin* | GABA analog | Risk of misuse  Risk associated with combining with other medications, particularly opioids | Avoid in patients with history of sedative use disorder |
| Hydroxyzine^†^ | Antihistamine | Avoid in patients with history of QTc prolongation | Avoid in older adults |
| Propranolol* | Beta-blocker | Contraindicated in bradycardia, greater than first-degree block; avoid in uncontrolled bronchial asthma  May be scheduled or dosed as needed for situational anxiety |  |
| **Chronic anxiety (GAD, panic, PTSD, social anxiety)** | | | |
| Buspirone^‡^ | 5HT1A receptor agonist | Not effective as an as needed agent  Only effective for GAD |  |
| SSRIs^§^ | Antidepressant^‖^ | May be anxiogenic upon initiation and dose increase; start at a low dose and titrate slowly  Variable interactions with other medications |  |
| SNRIs^§^ | Antidepressant^‖^ | May be anxiogenic upon initiation and dose increase; start at a low dose and titrate slowly  May increase blood pressure  Caution in uncontrolled hypertension |  |
| Mirtazapine^†^ | Serotonin and norepinephrine modulator | Not FDA approved for treatment of anxiety disorders  May be anxiolytic upon initiation  More sedating than SSRIs and SNRIs upon initiation |  |
| Prazosin* | Central alpha-1 antagonist | Approved for hypertension; may be used off-label for PTSD-related nightmares but not other symptoms of anxiety  Monitor blood pressure; avoid in hypotension |  |

This table presents some medications that can be considered for use as adjuncts to support management of anxiety-related symptoms during BZD tapering. Use in individual patients should always include review of medical and medication history and individual prescribing information to assess for any relative/absolute contraindications

*Not FDA approved for anxiety disorders. The CGC noted that gabapentin has potential for misuse and, therefore, while it may be useful in certain circumstances, should not be considered prior to other potential adjunctive medications

^†^FDA approved

^‡^FDA approved for GAD only

^§^Variably approved for GAD, panic disorder, PTSD, and social anxiety disorder

^‖^Antidepressants have boxed warnings regarding suicidality, especially in adolescents and emerging adults

**Table 10 Medications for Insomnia-Related Symptoms**

| **Medication*** | **Class/mechanism** | **Considerations for use^†^** | **Other population considerations** |
| --- | --- | --- | --- |
| Doxepin^‡^ | Antihistaminic tricyclic antidepressant | AASM approved for sleep maintenance insomnia^235,236^  Avoid in patients with suicidal ideation and behavior due to a risk of overdose | Caution in older adults, coronary artery disease, arrhythmia |
| Diphenhydramine^§^ | Antihistamine | AASM does not recommend for sleep onset or sleep maintenance insomnia^236^ | Avoid in older adults, may have paradoxical effects in children |
| Doxylamine^§^ | Antihistamine |  | Avoid in older adults, may have paradoxical effects in children |
| Hydroxyzine‖ | Antihistamine | Avoid in patients with history of QTc prolongation | Avoid in older adults |
| Melatonin^§^ | Sedative–hypnotic | AASM does not recommend for sleep onset or sleep maintenance insomnia^235,236^ | Avoid during pregnancy and breastfeeding; insufficient safety evidence |
| Ramelteon^‡^ | Agonist of melatonin receptors 1 and 2 | AASM approved for sleep onset insomnia^235,236^  Prone to significant interactions with CYP inhibitors and inducers |  |
| Trazodone‖ | Antidepressant | Start with lower doses to avoid orthostasis in older adults  AASM does not recommend for sleep onset or sleep maintenance insomnia^236^ | Use with caution in older adults |

This table presents some medications that can be considered for use as adjuncts to support management of insomnia-related symptoms during BZD tapering

*Non-BZD sedative–hypnotics (e.g., Z-drugs) are not recommended for patients with sleep issues who are undergoing BZD taper due to similar receptor action. Further information on adjunctive medications may be found on UpToDate, which has topics on benzodiazepine withdrawal and complementary and alternative treatments for anxiety symptoms and disorders: herbs and medications

^†^Use in individual patients should always include review of medical and medication history and individual prescribing information to assess for any relative/absolute contraindications

^‡^FDA approved

^§^FDA approved, available over the counter

^‖^Not FDA approved for insomnia

# Appendix 6. Pregnancy-Related Considerations

Tables 11 and 12 summarize specific considerations regarding BZD use and tapering during pregnancy. These tables are intended to be a resource for clinicians implementing this Guideline in individuals who are pregnant or lactating.

**Table 11 Relative Infant Dose (RID) of BZD Medications**

| **Medication*** | **RID^†^** |
| --- | --- |
| Alprazolam | 2–9%^237^ |
| Chlordiazepoxide | Unknown |
| Clonazepam | 2.5–4.6%^237^ |
| Clorazepate | Unknown, shares metabolite with diazepam |
| Diazepam | Up to 11%^238^ |
| Estazolam | Unknown |
| Flurazepam | Unknown |
| Lorazepam^‡^ | 0.7–4.4%^237^ |
| Oxazepam | 10–33%^239^ |
| Quazepam | 0.2–2.5%^240^ |
| Temazepam | Dose dependent 0–10%^241^ |
| Triazolam | Unknown |

This table outlines the relative infant dose of various BZD medications

*All BZDs are expected to cross placenta

^†^For optimal safety, the target RID is < 10%

^‡^Lorazepam is generally preferred in pregnancy and lactation due to its lack of active metabolites and low RID

**Table 12 Benzodiazepine Tapering Considerations by Pregnancy Trimester**

|  | **1st trimester** | **2nd trimester** | **3rd trimester** | **Postpartum** |
| --- | --- | --- | --- | --- |
| Potential fetal effects of BZDs | Minimal evidence of fetal malformations^242,243^  Increased risk of preterm birth |  | Increased risk of preterm birth, low birth weight, cesarean delivery, ventilatory support | Concern for withdrawal and potential fetal effects if high doses are used during lactation |
| Potential effects of pregnancy on BZD pharmacokinetics | Increased volume of distribution and CYP2C19, CYP3A4, and CYP2C9 metabolism (resulting in decreased effect)  Decreased CYP1A2 and CYP2C19 activity | Increased volume of distribution and CYP2C19, CYP3A4, and CYP2C9 metabolism (resulting in decreased effect)  Decreased CYP1A2 and CYP2C19 activity | Increased volume of distribution and CYP2C19, CYP3A4, and CYP2C9 metabolism (resulting in decreased effect)  Decreased CYP1A2 and CYP2C19 activity | Reversal of pregnancy changes may increase effect^244^ |
| Causes of insomnia | Nausea, urinary frequency, back pain | Fetal movements, heartburn, leg cramps, shortness of breath | Fetal movements, heartburn, leg cramps, shortness of breath | Infant care, pain |
| Considerations for tapering BZDs | If alternative planned (e.g., SSRI), start alternative early to allow 6–8 weeks for effect before tapering BZD. Per above, BZD effect may decrease even before taper |  | Lowest dose possible to avoid neonatal withdrawal | Monitor sleep closely |
| Alternative medication for insomnia | Diphenhydramine | Antihistamines, trazodone | Antihistamines, trazodone |  |
| Alternative medication for acute anxiety | Hydroxyzine* | Hydroxyzine | Hydroxyzine | Hydroxyzine |
| Alternative medication for severe chronic anxiety | SSRI | SSRI | SSRI^†^ | Sertraline has lowest RID |

This table outlines considerations for BZD tapering during each trimester of pregnancy and postpartum

*Limited data suggest possible low risk with first trimester use, but hydroxyzine is generally considered safe in practice

^†^Possible increase in persistent pulmonary hypertension of the newborn, with a number needed to harm of 1,000

# Appendix 7. Resources

## Resources for Screening for Substance Use

- Tobacco, Alcohol, Prescription medication, and other Substance use Tool (TAPS; https://nida.nih.gov/taps2/)

## Resources for Benzodiazepine Tapering Strategies

### Adjunctive Therapy

- How to find CBT-I resources (Endorsed by American Academy of Sleep Medicine)
  - Insomnia Toolkit for Clinicians (<https://aasm.org/clinical-resources/insomnia-toolkit/>)
  - Locate a CBT-I Provider (<https://cbti.directory>)

### Drug Interactions

- Flockhart Table™: Flockhart DA, Thacker, D., McDonald, C., Desta, Z. *The Flockhart Cytochrome P450 Drug-Drug Interaction Table*. Division of Clinical Pharmacology, Indiana University School of Medicine (Updated 2021). https://drug-interactions.medicine.iu.edu/. Accessed September 26, 2024.
  - This table includes drug interactions that are mediated by cytochrome P450 enzymes. Medications listed as minor substrates or mild inhibitors may not have clinically significant interactions.

### Taper Support

- Many patients noted that *The Ashton Manual* and *The Maudsley Deprescribing Guidelines* were helpful to read for BZD tapers.
  - Ashton CH. Benzodiazepines: How They Work and How to Withdraw (The Ashton Manual). Benzodiazepine Information Coalition; 2002.
  - Horowitz M, Taylor DM. *Chapter 3: Safe Deprescribing of Benzodiazepines and Z-drugs*. The Maudsley Deprescribing Guidelines: Antidepressants, Benzodiazepines, Gabapentinoids and Z-drugs. WILEY Blackwell; 2024.
  - Horowitz MA, Taylor D. How to reduce and stop psychiatric medication. Eur Neuropsychopharmacol. 2022;55:4-7. doi:10.1016/j.euroneuro.2021.10.001
- Liquid Tapering methods may help for smaller dose decreases.
  - BIC information: https://www.benzoinfo.com/benzodiazepine-tapering-strategies/
- Compounding pharmacies can create custom medications.
  - Alliance for Pharmacy Compounding (https://a4pc.org/)
- Educational information for patients may include, but are not limited to, https://mysleepwell.ca/ and EMPOWER programs such as ones from the VA (https://marketplace.va.gov/innovations/eliminating-medications-through-patient-ownership-of-end-results)

## Resources for Workforce Safety and Well-Being

- Workforce Safety and Well-Being Resource Center (<https://www.jointcommission.org/our-priorities/workforce-safety-and-well-being/resource-center/>)

## Resources for Population-Specific Considerations

### Patients Co-prescribed Benzodiazepines and Opioids

- [Risk Index for Overdose or Serious Opioid-Induced Respiratory Depression (RIOSORD)](https://www.naccho.org/uploads/downloadable-resources/MS-riosord-tool-toolkit47.pdf)
- Opioid Risk Tool – OUD (ORT-OUD) (<https://nida.nih.gov/nidamed-medical-health-professionals/screening-tools-resources/opioid-risk-tool-oud-ort-oud>). This is also available in: Cheatle MD, Compton PA, Dhingra L, Wasser TE, O'Brien CP. Development of the revised opioid risk tool to predict opioid use disorder in patients with chronic nonmalignant pain. J Pain. 2019 1;20(7):842-851.

### Patients with Benzodiazepine and/or Other Substance Use Disorders

#### Harm Reduction

- *Substance Abuse and Mental Health Services Administration: Harm Reduction Framework.* Center for Substance Abuse Prevention, Substance Abuse and Mental Health Services Administration; 2023. https://www.samhsa.gov/sites/default/files/harm-reduction-framework.pdf

### Older Adults

- 2023 American Geriatrics Society Beers Criteria Update Expert Panel. American Geriatrics Society 2023 updated AGS Beers Criteria(R) for potentially inappropriate medication use in older adults. *J Am Geriatr Soc*. Jul 2023;71(7):2052-2081. doi:10.1111/jgs.18372

### Patients Who Are Pregnant and Lactating

- [[Eat, Sleep, Console for NAS](https://www.cffutures.org/files/QIC_Resources/Learning_with_the_Expert/Eat_Sleep_console_manual_with_tools_Yale_Boston_NNEPQIN.pdf)](https://www.cffutures.org/files/QIC_Resources/Learning_with_the_Expert/Eat_Sleep_console_manual_with_tools_Yale_Boston_NNEPQIN.pdf): <https://www.cffutures.org/files/QIC_Resources/Learning_with_the_Expert/Eat_Sleep_console_manual_with_tools_Yale_Boston_NNEPQIN.pdf>
- ACOG Practice Bulletin: Clinical management guidelines for obstetrician-gynecologists number 92, April 2008 (replaces practice bulletin number 87, November 2007). Use of psychiatric medications during pregnancy and lactation. *Obstet Gynecol*. 2008;111(4):1001-20. doi:10.1097/AOG.0b013e31816fd910
- American College of Obstetricians and Gynecologists. Treatment and management of mental health conditions during pregnancy and postpartum: ACOG clinical practice guideline no. 5. *Obstet Gynecol*. 2023;141(6):1262-1288. doi:10.1097/aog.0000000000005202
- Shyken JM, Babbar S, Babbar S, Forinash A. Benzodiazepines in pregnancy. *Clin Obstet Gynecol*. 2019;62(1):156-167. doi:10.1097/GRF.0000000000000417
- Reproductive Psychiatry Resource & Information Center (<https://womensmentalhealth.org/>)
- Pregnancy and Breastfeeding Exposures (https://mothertobaby.org/pregnancy-breastfeeding-exposures/)

## Guidelines for the Treatment of Underlying Conditions

BZDs are prescribed for a variety of conditions. In most cases, other pharmacological and psychosocial interventions are more effective and associated with lower risk. This section includes references for CPGs for these underlying conditions that clinicians may consider incorporating into a given patient’s treatment plan before, during, or after BZD tapering.

### Insomnia

- Sateia MJ, Buysse DJ, Krystal AD, Neubauer DN, Heald JL. Clinical practice guideline for the pharmacologic treatment of chronic insomnia in adults: an American Academy of Sleep Medicine clinical practice guideline. J Clin Sleep Med. 2017;13(2):307–349.
- Edinger JD, Arnedt JT, Bertisch SM, et al. Behavioral and psychological treatments for chronic insomnia disorder in adults: an American Academy of Sleep Medicine clinical practice guideline. J Clin Sleep Med. 2021;17(2):255–262.
- Qaseem A, Kansagara D, Forciea M, Cooke M, Denberg TD; Clinical Guidelines Committee of the American College of Physicians. Management of chronic insomnia disorder in adults: a clinical practice guideline from the American College of Physicians. Ann Intern Med 2016;165(2):125-33. Epub 2016 May 3.

### Anxiety and Mood Disorders

- Baldwin DS, Anderson IM, Nutt DJ, Allgulander C, Bandelow B, den Boer JA, *et al.* Evidence-based pharmacological treatment of anxiety disorders, post-traumatic stress disorder and obsessive-compulsive disorder: a revision of the 2005 guidelines from the British Association for Psychopharmacology. *J Psychopharmacol* 2014;28:403–39.
- Gautam S, Jain A, Gautam M, Vahia VN, Gautam A. Clinical Practice Guidelines for the Management of Generalised Anxiety Disorder (GAD) and Panic Disorder (PD). Indian J Psychiatry. 2017 Jan;59(Suppl 1):S67-S73. doi: 10.4103/0019-5545.196975.
- National Collaborating Centre for Mental Health (UK). Generalised Anxiety Disorder in Adults: Management in Primary, Secondary and Community Care. Leicester (UK): British Psychological Society; 2011. PMID: 22536620.
- Melaragno AJ. Pharmacotherapy for anxiety disorders: from first-line options to treatment resistance. Focus. 2021;19(2):145-60.
- Canadian Coalition for Seniors’ Mental Health. Canadian Guidelines for the Assessment and Treatment of Anxiety in Older Adults. Toronto, Canada. 2024.

### PTSD

- Courtois CA, Sonis J, Brown LS, Cook J, Fairbank JA, Friedman M, Schulz P. Clinical practice guideline for the treatment of posttraumatic stress disorder (PTSD) in adults. American Psychological Association. 2017:119.
- Schnurr PP, Hamblen JL, Kelber M, Wolf J. VA/DoD Clinical Practice Guideline for Management of Posttraumatic Stress Disorder and Acute Stress Disorder. Department of Veterans Affairs and Department of Defense. 2023: Version 4.0.

### Seizure Disorders

- Kanner AM, Ashman E, Gloss D, Harden C, Bourgeois B, Bautista JF, Abou-Khalil B, Burakgazi-Dalkilic E, Llanas Park E, Stern J, Hirtz D. Practice guideline update summary: Efficacy and tolerability of the new antiepileptic drugs I: Treatment of new-onset epilepsy: Report of the Guideline Development, Dissemination, and Implementation Subcommittee of the American Academy of Neurology and the American Epilepsy Society. Neurology. 2018 Jul 10;91(2):74-81.
- Kanner AM, Ashman E, Gloss D, Harden C, Bourgeois B, Bautista JF, Abou-Khalil B, Burakgazi-Dalkilic E, Llanas Park E, Stern J, Hirtz D. Practice guideline update summary: Efficacy and tolerability of the new antiepileptic drugs II: Treatment-resistant epilepsy: Report of the Guideline Development, Dissemination, and Implementation Subcommittee of the American Academy of Neurology and the American Epilepsy Society. Neurology. 2018 Jul 10;91(2):82-90.

### Pain

- Katzberg HD, Khan AH, So YT. Assessment: Symptomatic treatment for muscle cramps (an evidence-based review) Report of the Therapeutics and Technology Assessment Subcommittee of the American Academy of Neurology. Neurology. 2010 Feb 23;74(8):691-6.
- NICE Guideline NG193 NI. Chronic pain (primary and secondary) in over 16s: assessment of all chronic pain and management of chronic primary pain. Methods. 2021 Apr;10
