# Supplementary material for: Joint Clinical Practice Guideline on Benzodiazepine Tapering: Considerations When Risks Outweigh Benefits
Source: J Gen Intern Med. 2025 Jun 17;40(12):2814–59. doi: 10.1007/s11606-025-09499-2 (PMC12463801; doi:10.1007/s11606-025-09499-2)
Supplement: Supplementary file 2 — Supplementary file2 (DOCX 760 KB) [file 11606_2025_9499_MOESM2_ESM.docx]

# S1. Glossary of Terms

**adjunctive interventions:** Interventions used in combination with a tapering strategy to treat withdrawal symptoms or otherwise support the tapering process.

**care partner:** A person who provides support to someone with a chronic condition to help them manage their healthcare needs. The term “care partner” emphasizes the support person’s role in shared decision-making with the patient and their providers and is preferred over “caregiver.”

**clinician:** A healthcare professional with the scope of practice to provide medical or clinical services.

**drug checking:** A harm reduction technique that provides drug composition information on drugs from the unregulated market (see **harm reduction**).

**drug testing:** The process of analyzing a biological specimen to check for the presence of chemicals that indicate exposure to selected substances.

**harm reduction:** A set of practical strategies and ideas aimed at reducing negative consequences associated with drug use.^1^

**high daily dose:** A BZD dose estimated as more than 15 mg diazepam equivalents (eg, >1.5 mg clonazepam, >3 mg lorazepam, >2 mg alprazolam). See Appendix H for BZD dose equivalents.

**inpatient treatment:** Intensive 24-hour-a-day medical services delivered in a hospital setting.^2^

**kindling:** The phenomenon of increasing severity of seizures with repeated episodes of withdrawal.

**level of care:** A discrete intensity of clinical services available in a given program or setting (see **setting**).

**low daily dose:** A BZD dose estimated as 10 mg diazepam equivalents or less (eg, ≤0.5mg clonazepam, ≤2 mg lorazepam, ≤1 mg alprazolam). See Appendix H for BZD dose equivalents.

**maintaining:** Stopping tapering dose reductions with no plan to further reduce the dose. Also referred to as a partial taper.

**medically managed setting:** A treatment setting in which care is led by a physician or advanced practice provider. In *The ASAM Criteria*, the primary focus of medically managed programs is treating withdrawal and/or stabilizing biomedical and psychiatric concerns while also providing the full spectrum of psychosocial services for patients who are able to participate effectively.

**moderate daily dose:** A BZD dose estimated as 10–15 mg diazepam equivalents (eg, 0.5–1.5 mg clonazepam, 2–3 mg lorazepam, 1–2 mg alprazolam). See Appendix H for BZD dose equivalents.

**patient:** A person receiving healthcare services.

**pausing:** Temporarily halting tapering dose reductions to allow time for withdrawal symptoms to subside.

**peer support:** A process of giving and receiving nonprofessional support from those with lived experience.

**physical dependence:** The state of an individual who has repeatedly taken a drug and experiences unpleasant physical symptoms if they stop taking it (see **withdrawal**).^3^

**polypharmacy:** The use of multiple medications concurrently.

**protracted withdrawal:** Withdrawal symptoms that persist beyond 6–8 weeks after discontinuing BZDs. Protracted withdrawal may result from a combination of physical and psychological dependence and the neurological effects of benzodiazepines. Also referred to as post-acute withdrawal.

**regular use:** Daily or near daily use of BZD.

**relative infant dose (RID):** The percent of a patient’s dose ingested by an infant who is fully breastfed.

**shared decision-making:** A process by which clinicians and patients work together to make decisions about a patient’s care, taking into account the available evidence and the patient’s needs, preferences, and values.

**short-term:** Use of BZD for less than a month.

**substance use disorder (SUD):** A medical illness consisting of a cluster of cognitive, behavioral, and physical symptoms caused by repeated use or misuse of a substance or substances. Characterized by clinically significant impairments in health and social function, as well as impaired control over substance use.^2,4^

**symptom-driven taper:** A withdrawal management strategy where medication is administered in response to withdrawal symptoms versus on a specific schedule.

**tolerance:** A condition in which a drug has diminished effect after persistent use.

**warm handoff:** A care transition in which the referring clinician facilitates a direct (ie, face-to-face and ideally in-person) introduction of the patient to the receiving clinician at their next level of care.

**withdrawal:** The collection of symptoms that occur after decrease or elimination of a drug.

Sources:

1. National Harm Reduction Coalition. Principles of Harm Reduction. Updated 2020. Accessed October 20, 2024. <https://harmreduction.org/about-us/principles-of-harm-reduction/>

2. US Department of Health and Human Services, Office of the Surgeon General. Facing Addiction in America: The Surgeon General’s Report on Alcohol, Drugs, and Health. US Department of Health and Human Services. Updated 2016. Accessed March 2, 2023. <https://addiction.surgeongeneral.gov/sites/default/files/surgeon-generals-report.pdf>

3. American Psychological Association. APA Dictionary of Psychology. Updated 11/15/2023. Accessed October 20, 2024. <https://dictionary.apa.org/physical-dependence>

4. American Society of Addiction Medicine. The ASAM National Practice Guideline for the Treatment of Opioid Use Disorder: 2020 Focused Update. *J Addict Med*. 2020;14(2S Suppl 1):1-91. doi:10.1097/adm.0000000000000633

# S2. Abbreviations and Acronyms

AACP American Association for Community Psychiatry

AAFP American Academy of Family Physicians

AAN American Academy of Neurology

AANP American Academy of Nurse Practitioners

AAPA American Academy of Physician Associates

AAPP American Association of Psychiatric Pharmacists

AASM American Academy of Sleep Medicine

ACMT American College of Medical Toxicology

ACOG American College of Obstetricians and Gynecologists

AGS American Geriatrics Society

AHRQ Agency for Healthcare Research and Quality

AMSTAR-2 Assessing the Methodological Quality of Systematic Reviews, revised

AMT anxiety management training

APA American Psychiatric Association

ASAM American Society of Addiction Medicine

ASQ Ask Suicide-Screening Questions

AUD alcohol use disorder

BIND benzodiazepine-induced neurological dysfunction

BWSQ Benzodiazepine Withdrawal Symptom Questionnaire

BZD benzodiazepine

CBT cognitive behavioral therapy

CBT-I cognitive behavioral therapy for insomnia

CDC US Centers for Disease Control and Prevention

CGC Clinical Guideline Committee

CINAHL Cumulative Index to Nursing and Allied Health Literature

CIWA-Ar Clinical Institute Withdrawal Assessment of Alcohol Scale, Revised

CIWA-B Clinical Institute Withdrawal Assessment Scale - Benzodiazepines

CNS central nervous system

CPG clinical practice guideline

CPG-MOS ASAM’s Clinical Practice Guideline Methodology Oversight Committee

C-SSRS Columbia Suicide Severity Rating Scale

CYP cytochrome P450

DEA US Drug Enforcement Agency

DoD US Department of Defense

ECHO Extension for Community Healthcare Outcomes

ED emergency department

EHR electronic health record

EMPOWER Eliminating Medications through Patient OWnership of End Results study

EMTALA Emergency Medical Treatment and Active Labor Act

EtD Evidence to Decision

FDA US Food and Drug Administration

FIS floppy infant syndrome

GABA gamma-aminobutyric acid

GAD generalized anxiety disorder

GCMS gas chromatography–mass spectrometry

GRADE Grading of Recommendations Assessment, Development, and Evaluation

GRADEpro GRADE profiler software

HPSO highly potent synthetic opioid

LLC limited liability company

LOCUS Level of Care Utilization System

MI motivational interviewing

MOUD medications for opioid use disorder

NAS neonatal abstinence syndrome

NIH National Institutes of Health

NSDUH National Survey on Drug Use and Health

OUD opioid use disorder

PDMP prescription drug monitoring program

PICO Population, Intervention, Comparator, Outcome

PRISMA Preferred Reporting Items for Systematic reviews and Meta-Analyses

PROSPERO International Prospective Register of Systematic Reviews

PTSD posttraumatic stress disorder

QIC ASAM’s Quality Improvement Council

RCT randomized controlled trial

REM rapid eye movement

RevMan Cochrane Review Manager

RID relative infant dose

RIOSORD Risk Index for Overdose or Serious Opioid-induced Respiratory Depression

RoB2 Cochrane Risk of Bias 2

SAMHSA Substance Abuse and Mental Health Services Administration

SSRI selective serotonin reuptake inhibitor

SUD substance use disorder

VA US Department of Veterans Affairs

VHA US Veterans Health Administration

YAWNS NB Your Answers When Needing Sleep in New Brunswick study

# S3. Methodology

A systematic literature review was conducted to establish a foundation of evidence for the recommendations in this Guideline. Methods followed current best practices for systematic reviews from the Agency for Healthcare Research and Quality (AHRQ), including screening and data extraction in duplicate, risk of bias assessment using standardized instruments, and a synthesized narrative summary of findings.^1^ In accordance with the Preferred Reporting Items for Systematic reviews and Meta-Analyses (PRISMA) standards, the systematic review was registered prospectively in the International Prospective Register of Systematic Reviews (PROSPERO) database (Identification Number: CRD42023408418).^2^

The literature review informed the deliberations of a committee of experts, the CGC, as they developed recommendation statements that consider an intervention’s clinical benefits and harms, as well as patient values and preferences. The GRADE method was used to develop recommendations in areas with sufficient evidence.^3^ Where evidence was lacking, a modified Delphi process was used to develop clinical consensus statements.^4^ As relatively little research has been published on discontinuation of BZD prescriptions in patients with physical dependence, this strategy allowed for the inclusion of guidance in areas with highly limited evidence.

## Clinical Practice Guideline Team

### Clinical Guideline Committee Formation and Oversight

ASAM’s QIC and CPG-MOS oversaw the development of this Guideline. The FDA provided guidance on but did not dictate the content and development of the Guideline. The QIC, working with partner medical societies and the FDA, oversaw the appointment of clinicians with broad subject matter expertise across medicine, psychiatry, and pharmacology representing regional and demographic diversity to the CGC. Partner medical and professional societies included:

- American Academy of Family Physicians (AAFP)
- American Academy of Neurology (AAN)
- American Academy of Physician Associates (AAPA)
- American College of Medical Toxicology (ACMT)
- American Association of Nurse Practitioners (AANP)
- American Association of Psychiatric Pharmacists (AAPP)
- American College of Obstetricians and Gynecologists (ACOG)
- American Geriatrics Society (AGS)
- American Psychiatric Association (APA)

A list of CGC members, their areas of expertise, and conflict of interest disclosures are available in [Appendix D](#_Appendix_D._Disclosures). Members of the CPG-MOS and ASAM’s Ethics Committee reviewed disclosures of interest. No members of the CGC had high level conflicts of interest in relation to the Guideline topic. One member (BS) was determined to have a moderate conflict of interest due to the potential for industry profit from education on the Guideline delivered through their limited liability company (LLC). As a mitigation strategy, this member was asked not to accept financial or any other compensation from for-profit or industry groups for speaking engagements related to the topic of this Guideline for a period of 24 months following the publication of the Guideline.

### Patient Panel

ASAM asked leading patient advocacy organizations to nominate representatives to serve on a panel of individuals with lived experience with BZD discontinuation (the Patient Panel). The Patient Panel was engaged during the development process, providing input on the following, in parallel with the CGC:

- Key clinical questions
- Critical and important outcomes
- Recommendation statements
- Full text of the Guideline

This feedback was considered prior to finalization.

The Patient Panel met in combination with the CGC once and separate from the CGC four times. The initial meeting with the Patient Panel (June 2023) and the meeting in combination with the CGC (July 2023) focused on Patient Panel member introductions and lived experiences. The second meeting with the Patient Panel (February 2024) focused on receiving feedback on the initial internal draft of recommendations. The third meeting with the Patient Panel (July 2024) received their feedback on the public comment full guideline draft. The fourth meeting with the Patient Panel (November 2024) focused on how their feedback was incorporated into the final draft prior to approval and endorsement.

## Key Questions and Outcome Development

The CGC, with input from the FDA and Patient Panel, identified the following key clinical questions to be addressed by the systematic review and Guideline:

1. What is the efficacy and/or safety of tapering strategies for BZDs?
2. What factors influence the outcomes of BZD tapering and should be monitored?
3. How can shared decision-making and patient-centered health care be utilized to support the effectiveness and safety of BZD tapering?

These questions were used to develop a Population, Intervention, Comparator, Outcome (PICO) framework for identifying relevant research literature to answer each of the key clinical questions.

- **Population:** Adults who have been using one or more BZD medication for at least 2–4 weeks, including those with Benzodiazepine Use Disorder
- **Intervention:** Two types of interventions were considered:
  - Interventions that promote the successful discontinuation of BZD use
  - Interventions that manage withdrawal symptoms when discontinuing BZDs
- **Comparator:** Alternative interventions, treatment as usual, placebo, or active control condition
- **Outcome:** BZD cessation or dose reduction, BZD withdrawal severity, recurrence or rebound of BZD-indicated conditions (eg, insomnia, anxiety), sleep problems, cognition, mood, quality of life and patient satisfaction, global functioning, study attrition, other substance use, and adverse events

## Literature Review

The following databases were searched during March and April 2023: EMBASE, PsycINFO, PubMed, and Cumulative Index to Nursing and Allied Health Literature (CINAHL). Search strategies can be found in Tables S3.1-S3.5. The search was limited to controlled trials, cohort studies with a comparison condition, and systematic reviews of RCTs published in English on January 1, 2000, or later. To be included, studies had to have at least 20 adult participants using one or more BZDs at baseline for at least 2 weeks and include a BZD discontinuation strategy aimed at patients (ie, not targeting healthcare systems or provider prescribing behavior). Articles were reviewed in duplicate for inclusion at the title, abstract, and full-text levels (see Figure C1). Discussion and consensus between two research associates resolved uncertainty about article inclusion. Hand-searching for included publications was also completed. Details of the systematic literature review will be published separately. An overview of included articles is included in Table S3.6.

Three supplemental searches were conducted on predictors for developing BZD withdrawal, patient preferences and values, and validated BZD withdrawal scales. A grey literature search was conducted to search websites for BZD-related literature. The CGC and Patient Panel also provided grey literature.

Table S3.1. PubMed Search Strategy

| **PubMed Completed February 3, 2023** | | | |
| --- | --- | --- | --- |
| 1 | (benzodiazepine* OR z-drug OR alprazolam OR bromazepam OR clobazam OR clonazepam OR chlordiazepoxide OR clorazepate OR diazepam OR estazolam OR flunitrazepam OR flurazepam OR halazepam OR midazolam OR medazepam OR lorazepam OR nitrazepam OR oxazepam OR prazepam OR quazepam OR temazepam OR triazolam OR zolpidem OR zopiclone OR eszopiclone OR zaleplon)  *Search in Title/Abstract* | 77,807 | Eliminated: sedative’ or ‘hypnotic*’ OR BZD-agonist |
|  | | | |
| 2 | (taper* OR withdraw* OR discontinu* OR deprescribe* OR cessation) *Search in Title/Abstract* | 382,582 | Eliminated: detoxif* OR depend* OR “long term” OR reduc* |
| **Combine and filter for English, publication date and Publication Type** | | | |
| 3 | 1 AND 2 *Filters applied: English, from 2000/1/1 - 2023/3/23 [or present date]* | 3803 |  |
| **4** | **Add filters*: Clinical Study, Clinical Trial, Guideline, Meta-Analysis, Observational Study, Practice Guideline, Pragmatic Clinical Trial, Randomized Controlled Trial, Systematic Review*** | **780** |  |
| **Search for studies missed by Publication Type filters** | | | |
|  | CLEAR ALL PUBLICATION TYPE FILTERS |  |  |
| 5* | "Cohort Studies"[Mesh] OR "Risk Assessment"[Mesh] OR Therapy/Narrow[filter] OR Clinical Prediction Guides/Broad[filter]. *Filters applied: English, from 2000/1/1 - 2023/3/23* | 6,359,097 |  |
| **6** | **5 and 3** | **1,694** |  |
| **Combine two main searches** | | | |
| 7 | 6 OR 4 | 1884 |  |

Table S3.2. Supplemental PubMed Search Strategy

| **Supplemental Pubmed (non-indexed article search) completed February 14, 2023**  **Drugs of interest limited to Title/Abstract** | | | |
| --- | --- | --- | --- |
| 1 | (benzodiazepine* OR z-drug OR alprazolam OR bromazepam OR clobazam OR clonazepam OR chlordiazepoxide OR clorazepate OR diazepam OR estazolam OR flunitrazepam OR flurazepam OR halazepam OR midazolam OR medazepam OR lorazepam OR nitrazepam OR oxazepam OR prazepam OR quazepam OR temazepam OR triazolam OR zolpidem OR zopiclone OR eszopiclone OR zaleplon)  *Search in Title/Abstract* | 77,807 |  |
|  | | | |
| 2 | (taper* OR withdraw* OR discontinu* OR deprescribe* OR cessation) *Search in Title/Abstract* | 382,582 |  |
| **Combine and filter for English, publication date and Publication Type** | | | |
| 3 | 1 AND 2 | 6776 |  |
|  | #3 *Sorted by Publication Date (diminishing recency). Filters: from 2022/10/1 - 2023/2/3* | 117 | This search picks up any article published within the 4 months prior to the main PubMed search on 2-3-22 that was as-yet not indexed on that date. Results were exported for Annie’s addition to Endnote. |

Table S3.3. PsychInfo Search Strategy

| **PsychInfo Completed February 7, 2023**  **Drugs of interest limited to Title/Abstract** | | | |
| --- | --- | --- | --- |
| 1 | (benzodiazepine* OR z-drug OR alprazolam OR bromazepam OR clobazam OR clonazepam OR chlordiazepoxide OR clorazepate OR diazepam OR estazolam OR flunitrazepam OR flurazepam OR halazepam OR midazolam OR medazepam OR lorazepam OR nitrazepam OR oxazepam OR prazepam OR quazepam OR temazepam OR triazolam OR zolpidem OR zopiclone OR eszopiclone OR zaleplon)  *Search in Title/Abstract* | 21,375 |  |
|  | | | |
| 2 | (taper* OR withdraw* OR discontinu* OR deprescribe* OR cessation) *Search in Title/Abstract* | 85,144 |  |
| **Combine and filter for English, publication date and Publication Type** | | | |
| 3 | 1 AND 2 *Filters applied: English, 2000-2023 [or present date]* | 1802 |  |
| 4 | Narrow 3 by methodology categories: *empirical study, quantitative study, longitudinal study, retrospective study, clinical trial, treatment outcome, systematic review, prospective study, meta-analysis, meta-synthesis* | 998 |  |
| **Search for studies missed by Methodology filters** | | | |
| 5 | Limit **3** by Major Subject: *benzodiazepine*s | 467 |  |
| 6 | Limit **3** by Major Subject: *drug withdrawal* | 260 |  |
| 7 | 5 AND 7 | 86 |  |
| **Combine two main searches** | | | |
| 8 | 4 OR 7 | 1026 | Search #3 (n=1802) was sorted by relevance. Articles of interest (n=10) were selected from the first 50 articles. All 10 could be found in the yield of search #8. |

Table S3.4. CINAHL Search Strategy

| **Cumulated Index to Nursing and Allied Health Literature (CINAHL) Completed February 14, 2023**  **Drugs of interest limited to Title/Abstract** | | | |
| --- | --- | --- | --- |
| 1 | (benzodiazepine* OR z-drug OR alprazolam OR bromazepam OR clobazam OR clonazepam OR chlordiazepoxide OR clorazepate OR diazepam OR estazolam OR flunitrazepam OR flurazepam OR halazepam OR midazolam OR medazepam OR lorazepam OR nitrazepam OR oxazepam OR prazepam OR quazepam OR temazepam OR triazolam OR zolpidem OR zopiclone OR eszopiclone OR zaleplon)  *Search in Title, then in Abstract; combine with ‘OR’* | 13,241 |  |
|  | CLEAR SEARCH BOXES |  |  |
| **Discontinuation terms of interest limited to Title/Abstract** | | | |
| 2 | (taper* OR withdraw* OR discontinu* OR deprescribe* OR cessation)  *Search in Title, then in Abstract; combine with ‘OR’* | 89,154 |  |
|  | CLEAR SEARCH BOXES |  |  |
| **Combine and filter for English, publication date and Publication Type** | | | |
| 3 | 1 AND 2 *Filters applied: English, from 2000-2023*  *(These filters are from the lefthand pane when viewing the View Results pane.)* | 1438 | These are the results that were exported and then imported into Annie’s Endnote library. |
| **Exclude MEDLINE records** | | | |
| 4 | Click ‘Edit’ button to the right of search #3 in the Search History pane to access Advanced Search options  Check ‘Exclude MEDLINE’  Click ‘Save’ button at bottom | 546 | Results from this final step were not used because of problems with export. |

Table S3.5. Embase Search Strategy

| **Embase Completed March 10, 2023**  **DRUGS of INTEREST LIMITTED to TITLE and ABSTRACT** | | | |  |
| --- | --- | --- | --- | --- |
| 1 | (benzodiazepine* OR z-drug OR alprazolam OR bromazepam OR clobazam OR clonazepam OR chlordiazepoxide OR clorazepate OR diazepam OR estazolam OR flunitrazepam OR flurazepam OR halazepam OR midazolam OR medazepam OR lorazepam OR nitrazepam OR oxazepam OR prazepam OR quazepam OR temazepam OR triazolam OR zolpidem OR zopiclone OR eszopiclone OR zaleplon)  *Search in Title/Abstract* | 99,689 | NOT: ‘hypnotic’ OR ‘sedative’ OR ‘BZD-agonist |  |
| 2 | (taper* OR withdraw* OR discontinu* OR deprescribe* OR cessation)  *Search in Title/Abstract* | 327,291 | NOT detoxif* OR depend* OR “long term” OR reduc* |  |
| **COMBINE SEARCH 1 and 2** | | | | |
| 3 | (benzodiazepine* OR z-drug OR alprazolam OR bromazepam OR clobazam OR clonazepam OR chlordiazepoxide OR clorazepate OR diazepam OR estazolam OR flunitrazepam OR flurazepam OR halazepam OR midazolam OR medazepam OR lorazepam OR nitrazepam OR oxazepam OR prazepam OR quazepam OR temazepam OR triazolam OR zolpidem OR zopiclone OR eszopiclone OR zaleplon) *Search in Title/Abstract*  AND  (taper* OR withdraw* OR discontinu* OR deprescribe* OR cessation) *Search in Title/Abstract* | 4,529 | NOT: ‘hypnotic’ OR ‘sedative’ OR ‘BZD-agonist  NOT detoxif* OR depend* OR “long term” OR reduc* |  |
| **FILTER SEARCH 3 for: EMBASE, ENGLISH, PUBLICATION DATE, PUBLICATION TYPE** | | | | |
| 4 | Filter for EMBASE | 4,198 |  |  |
| 5 | *Filter for: EMBASE and  English, from 2000 - 2023* | 2,867 |  |  |
| 6 | Filter for*: EMBASE and  English, from 2000 – 2023 and Clinical Study* | 1,976 |  |  |

Figure S3.1. PRISMA diagram for systematic literature review


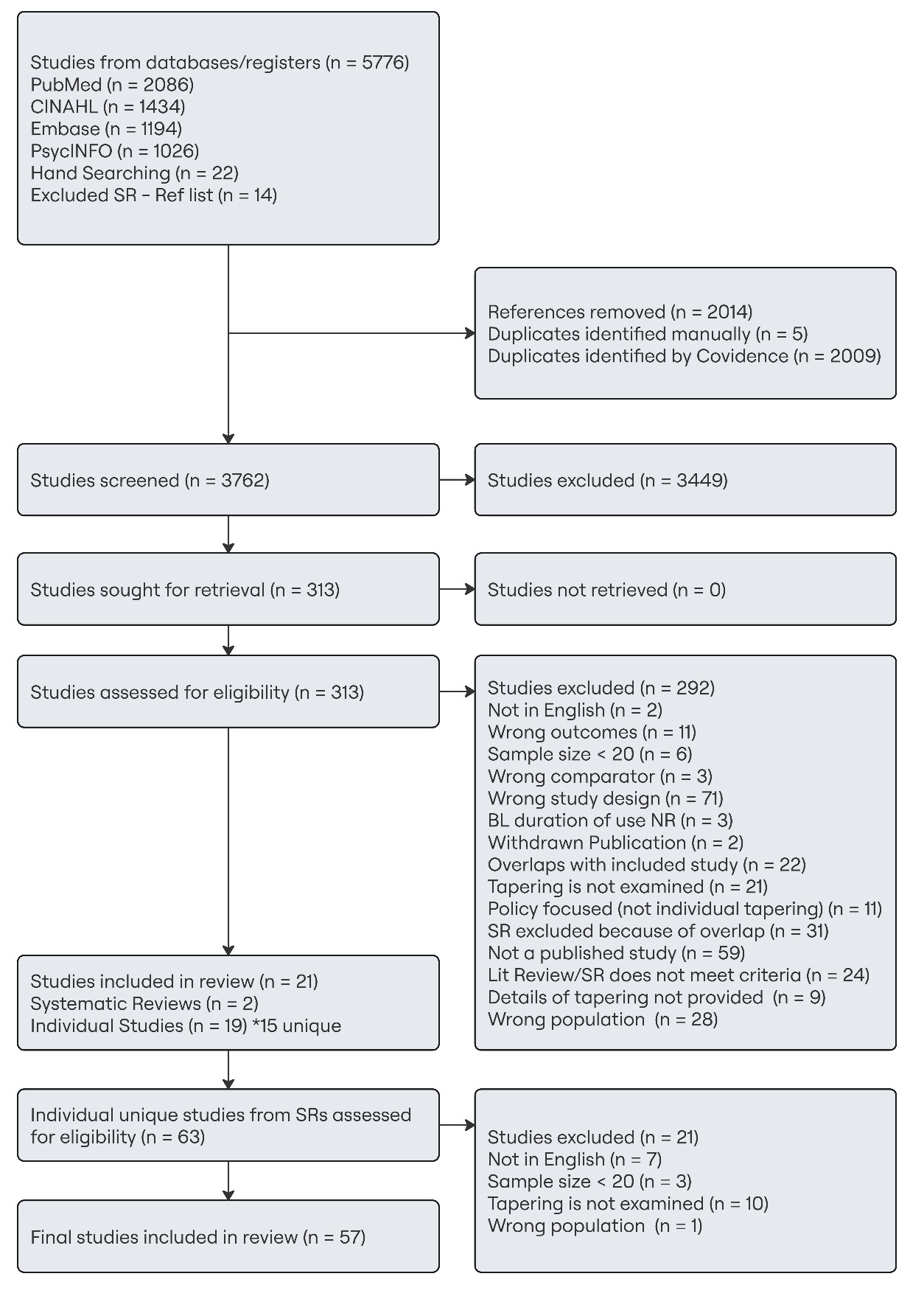


Table S3.6. Included Studies Overview

| **Study** | **Intervention** | **Comparison** | **Population** | **Design** | **N** |
| --- | --- | --- | --- | --- | --- |
| **Abrupt Cessation** | | | | | |
| Gerra 2002^5^ | Flumazenil; Taper | Flumazenil + Rapid Taper vs Placebo + Gradual Taper vs Placebo + Abrupt Cessation | Adults who contacted the Substance Abuse Center to request BZD withdrawal services | RCT | 50 |
| Petrovic 2002^6^ | Taper | Rapid Taper vs Abrupt Cessation | Older adults taking BZDs daily for at least 3 months | RCT | 40 |
| **Adjunctive Medication** | | | | | |
| Lader 1993^7^ | Alpidem | Alpidem + Taper vs Placebo +Taper | Adults referred for help in discontinuing their long-term use of BZDs after they experienced difficulty attempting to reduce their dose. | RCT | 25 |
| Ashton 1990^8^ | Buspirone | Buspirone + Taper vs Placebo + Taper | Adults taking continuous BZDs for at least 6 months who wished to withdraw from BZDs | RCT | 23 |
| Lader 1987^9^ | Buspirone | Buspirone + Taper vs Placebo + Taper | Adults referred for help discontinuing their long-term use of BZD after they experienced difficulty attempting to reduce their dose. | RCT | 24 |
| Morton 1995^10^ | Buspirone | Buspirone + Taper vs Placebo + Taper | Adults referred to the BZD Withdrawal Clinic for help with stopping BZD use. | RCT | 24 |
| Udelman 1990^11^ | Buspirone | Buspirone + Taper vs Placebo + Taper | Adults receiving alprazolam for moderate (or greater) anxiety for at least 3 months | RCT | 36 |
| Rickels 2000^12^ | Buspirone; Imipramine | Buspirone + Taper vs Imipramine + Taper vs Placebo + Taper | Adults with GAD taking BZDs for at least 12 months | RCT | 107 |
| Rynn 2003^13^ | Buspirone; Imipramine | Buspirone + Taper vs Imipramine + Taper vs Placebo + Taper | Adults seeking to discontinue their long-term use of BZD to treat panic disorder. | RCT | 40 |
| Mercier-Guyon 2004^14^ | Captodiame | Captodiame + Taper vs Placebo +Taper | Adults prescribed BZDs to treat anxiety disorder for at least 6 months. | RCT | 81 |
| Klein 1994^15^ | Carbamazepine | Carbamazepine + Taper vs Placebo +Taper | Adults with GAD or panic disorder referred to an anxiety disorder clinic (open trial of alprazolam for 2-months) | RCT | 71 |
| Schweizer 1991^16^ | Carbamazepine | Carbamazepine + Taper vs Placebo + Taper | Adults with a history of difficulty discontinuing their long-term BZD use | RCT | 40 |
| Lemoine 2006^17^ | Cyamemzine | Cyamemzine + Abrupt cessation vs Taper | Adults with anxiety disorder taking BZD for at least 3 months | RCT | 244 |
| Tyrer 1996^18^ | Dothiepin | Dothiepin + Taper vs Placebo + Taper | Outpatients with putative BZD dependence. | RCT | 87 |
| Malsch 2001^19^ | Kava lactone | Kava lactone + Taper vs Placebo +Taper | Adults taking BZD for phobic disorder, GAD, or adaptation disturbance for at least 14 days | RCT | 40 |
| Baandrup 2016a^20^ | Melatonin | Melatonin + Taper vs Placebo + Taper | Adults with schizophrenia or bipolar disorder taking BZD for at least 3 months | RCT | 80 |
| Cardinali 2002^21^ | Melatonin | Melatonin + Taper vs Placebo + Taper | Older adults with minor sleep disturbances who regularly take low-dose BZD | RCT | 45 |
| Garfinkel 1999^22^ | Melatonin | Melatonin + Taper vs Placebo + Taper | Older adults taking BZD daily for insomnia for more than 6 months | RCT | 34 |
| Lähteenmäki 2013^23^ | Melatonin | Melatonin + Taper vs Placebo + Taper | Older adults prescribed BZD for primary insomnia, taken regularly at night for at least 1 month | RCT | 92 |
| Peles 2007^24^ | Melatonin | Melatonin + Taper vs Placebo + Taper | Adults who used non-prescribed BZDs, had opioid dependence, and reported self-administration of illicit heroin for 1 year or more. | RCT | 80 |
| Vissers 2007^25^ | Melatonin | Melatonin + Taper vs Placebo + Taper | Adults with insomnia taking BZD as a sleeping medication at least 3 days per week for more than 3 months | RCT | 38 |
| Romach 1998^26^ | Ondansetron | Ondansetron + Taper vs Placebo + Taper | Adults seeking to discontinue their long-term daily use of alprazolam or lorazepam. | RCT | 187 |
| GlaxoSmithKline 2002^27^ | Paroxetine | Paroxetine + Taper vs Placebo + Taper | Adults with anxiety disorder taking BZD for at least 6 months | RCT | 54 |
| Nakao 2006^28^ | Paroxetine | Paroxetine + Taper vs Taper only vs BZD Continuation | Adults without major depression taking BZD for at least 3 months | RCT | 45 |
| Zitman 2001^29^ | Paroxetine | Paroxetine + Taper vs Placebo + Taper | Adults with major depressive disorder taking BZD daily for at least 3 months | RCT | 230 |
| Hadley 2012^30^ | Pregabalin | Pregabalin + Taper vs Placebo +Taper | Adult outpatients with GAD receiving stable treatment with a BZD for at least 8 weeks | RCT | 106 |
| Schweizer 1995^31^ | Progesterone | Progesterone + Taper vs Placebo +Taper | Adults taking BZD daily for at least one year | RCT | 43 |
| Rickels 1999^32^ | Trazodone; Valproate | Trazodone + Taper vs Valproate +Taper vs Placebo + Taper | Adults on continuous daily treatment with diazepam, lorazepam, or alprazolam for a minimum of 1 year | RCT | 78 |
| Vorma 2011^33^ | Valproate | Valproate + Taper vs Taper only | Adults with opioid dependence and BZD dependence | RCT | 30 |
| Pat-Horenczyk 1998^34^ | Zopiclone | Zopiclone + Taper vs Delayed Taper | Adults with a history of long-term use of flunitrazepam to treat insomnia | RCT | 24 |
| **Adjunctive Psychosocial** | | | | | |
| Elliott 2005^35^ | CBT | CBT + Taper + Skills Reinforcement vs CBT + Taper | Adults who used illicit drugs, undergoing mandatory reduction of BZD prescription in Scotland | RCT | 53 |
| Gosselin 2006^36^ | CBT | CBT + Taper vs Non-CBT therapy + Taper | Adults seeking to discontinue their long-term use of BZD to treat GAD | RCT | 61 |
| O'Connor 2008^37^ | CBT | CBT + Non-CBT therapy + Taper vs Non-CBT therapy + Taper vs Taper only | Adults taking BZD for at least 2 years | RCT | 89 |
| Otto 1993^38^ | CBT | CBT + Taper vs Taper only | Adult outpatients seeking treatment to discontinue their long-term use of BZD to treat panic disorder. | RCT | 33 |
| Otto 2010^39^ | CBT | CBT + Taper vs Relaxation training + Taper vs Taper only | Adult outpatients seeking to discontinue their long-term use of BZD | RCT | 47 |
| Oude Voshaar 2003a^40^ | CBT | CBT + Taper vs Taper only vs TAU | Adults taking BZD regularly for at least 3 months and are unable to stop BZD use on their own. | RCT | 180 |
| Spiegel 1994^41^ | CBT | CBT + Taper vs Taper | Adults with panic disorder with agoraphobia of at least 6 months and taking alprazolam | RCT | 21 |
| Vorma 2002^42^ | CBT | CBT + Taper vs Physician Taper | Adults seeking treatment for a primary diagnosis of BZD dependence | RCT | 76 |
| Baillargeon 2003^43^ | CBT-I | CBT-I + Taper vs Taper only | Older adults with insomnia taking BZD daily for at least 3 months | RCT | 65 |
| Belleville 2007^44^ | CBT-I | CBT-I + Taper vs Taper only | Adults with sleep difficulties taking BZDs and/or z-drugs | RCT | 53 |
| Coteur 2022^45^ | CBT-I | CBT-I + Taper vs TAU | Adults who have taken BZD continuously for at least 6 months | RCT | 727 |
| Lui 2021^46^ | CBT-I | CBT-I + Taper vs Taper only | Charts reviewed for patients referred to a pharmacist for sedative-hypnotic deprescribing | Retrospective chart review | 111 |
| Morin 2004^47^ | CBT-I | CBT-I + Taper vs CBT-I only vs Taper only | Older adults with chronic insomnia taking BZD for sleep on more than 50% of nights for at least 3 months | RCT | 76 |
| Yeung 2019^48^ | Electroacupuncture | Electroacupuncture + Taper vs Placebo + Taper | Adults with an included psychiatric diagnosis who were willing to taper their regular BZD use. | RCT | 144 |
| Barros 2022^49^ | Mindfulness | Brief hypnotic use education + Mindfulness-Based Relapse-Prevention vs Brief education alone | Adult women taking a hypnotic at least 4 times per week for at least 3 months | RCT | 52 |
| Elsesser 1996^50^ | Relaxation Training | Multi-symptom management training + Taper vs Anxiety management training + Taper | Adults with chronic BZD use with one or more prior attempts at discontinuing use | RCT | 44 |
| Gilbert 1993^51^ | Relaxation Training | Relaxation training + Reduction encouragement vs BZD Continuation | Residents of two aged care facilities | Prospective cohort study, Comparison group design. | 60 |
| **Patient Education** | | | | | |
| Tannenbaum 2014^52^ | EMPOWER | Self-taper booklet (EMPOWER) vs TAU | Older adults with polypharmacy taking a BZD continuously for at least 3 months | RCT | 303 |
| Gorgels 2005^53^ | Patient Education | Taper letter + Consultation offer vs BZD Continuation | Adults with long-term BZD use identified in the electronic medical record | Prospective controlled cohort trial | 3528 |
| Heather 2004^54^ | Patient Education | Consultation + Self-help information vs Taper letter vs BZD Continuation | Adults who have used BZDs continuously for at least 6 months | RCT | 272 |
| Ten Wolde 2008^55^ | Patient Education | Tailored taper letter vs Multiple tailored taper letters vs Generic taper letter | Adults with chronic BZD use | RCT | 508 |
| Vicens 2006^56^ | Structured Intervention | Structured Intervention + Taper plan + Follow-up visits vs TAU | Adolescents and adults taking BZDs at least 5 times per week for over a year | RCT | 139 |
| Vicens 2014^57^ | Structured Intervention | Structured Intervention + Taper plan + Follow-up visits vs Structured Intervention + Taper plan + Written Instructions vs TAU | Adults taking BZD daily for at least 6 months | RCT | 532 |
| **Rapid Taper Support** | | | | | |
| MacDonald 2022^58^ | Flumazenil | Flumazenil + Symptom-triggered treatment vs Placebo + Symptom-triggered treatment | Adults taking >10 mg diazepam equivalents BZD daily for at least 3 months. | RCT | 26 |
| **Taper** | | | | | |
| Gopalan 2019^59^ | Taper | Gradual taper + Symptom-triggered treatment vs Symptom-triggered treatment | Adults admitted to an inpatient obstetrical unit with a psychiatry consult for BZD withdrawal. | Retrospective cohort study | 118 |
| McGregor 2003^60^ | Taper | Taper vs Symptom-triggered treatment | Adults seeking treatment for BZD withdrawal who need tapered withdrawal management in an in-patient setting. | RCT | 44 |
| Curran 2003^61^ | Taper (immediate or delayed) | Immediate Taper vs Delayed Taper vs BZD Continuation | Older adults treating sleep difficulties with daily BZD for at least 6 months, identified via patient record search. | RCT | 138 |
| CBT = cognitive behavioral therapy; RCT = randomized controlled trial; TAU = treatment as usual | | | | | |

## Evidence Review

A risk of bias assessment was completed for each included study (n = 57). Quality was rated using the Assessing the Methodological Quality of Systematic Reviews, revised (AMSTAR-2) tool for systematic reviews, the Cochrane Risk of Bias 2 (RoB 2) tool for randomized trials, and the National Institutes of Health (NIH) tool for observational cohort studies.^62-64^

The CGC was provided with key information about study methods, risk of bias ratings, and narrative syntheses of the results for each intervention described in the literature review. When the CGC determined the evidence for an intervention was sufficient to potentially lead to a recommendation, the relevant study results were extracted into Cochrane Review Manager (RevMan) software.^65^ Following the best practices outlined in the *Cochrane Handbook for Systematic Reviews of Interventions*, outcome data were pooled and uploaded into GRADE profiler (GRADEpro) software to construct Summary of Findings tables and aid in assessing the quality of evidence for an intervention.^66,67^

For dichotomous outcomes, fixed-effects Mantel-Haenszel techniques were used to calculate risk ratios. Peto odds ratios were calculated instead in cases where the event rate was <1% in one study arm. The risk difference was used instead if both arms had zero events. For continuous outcomes, fixed effects inverse variance techniques were used to calculate mean differences. If multiple scales were used, the standardized mean difference was calculated.

The certainty of evidence was rated as high, moderate, or low, based on the quality (ie, risk of bias) of the included studies, the consistency and precision of the studies’ results, the direct relevance of the studies to the key questions, and the potential for publication bias. The certainty of evidence reflects a level of confidence—or certainty—in how closely effect estimates reflect the true effect and, therefore, the extent to which the evidence can be relied upon when making recommendation decisions.

## Recommendation Development

The CGC’s decisions on whether a recommendation could be made were based on the available evidence and judgments on the recommendation’s expected benefits and harms and its acceptability and feasibility to potential stakeholders. The CGC compiled EtD tables to document the evidence and their judgments for these recommendations (see [Appendix E](#_Appendix_E._Evidence_1)). The CGC based decisions on their clinical expertise when clinical evidence for a recommendation was of low quality, unclear, or nonexistent. In these instances, the CGC’s clinical expertise guided decisions on whether a recommendation could still be made or should be delayed until further evidence has been produced. The CGC also considered whether failing to make a recommendation could lead to potential harm. The CGC also considered the expected clinical impact, acceptability, and feasibility of consensus-based recommendations. Consensus-based recommendations were labeled with “Clinical Consensus,” whereas evidence-based statements include a certainty of evidence rating. A 70% agreement among CGC members was required to approve a recommendation.

The CGC graded the strength of each accepted recommendation as strong or conditional based on the overall balance of risks and benefits, the certainty of the evidence on treatment effects, and patient preferences and values. The CGC worded recommendations to reflect the strength of the statement. For example, “clinicians should” indicates a strong recommendation, while “clinicians can consider” indicates a conditional recommendation. The CGC voted on the recommendations to determine the strength of each statement, with a 70% threshold required for consensus.

## External Review

An external review period was conducted prior to publication. ASAM invited major stakeholder organizations, partner organizations, relevant committees, and its Board of Directors to provide comments, and ASAM worked with partner organizations and the FDA to broadly disseminate a call for public comment. The CGC and Patient Panel also provided comments. All comments were combined into an Excel file and summarized by concern. Together with ASAM staff, the CGC reviewed all comments and updated the Guideline as appropriate.

Sources:

1. Agency for Healthcare Research and Quality. Methods Guide for Effectiveness and Comparative Effectiveness Reviews. 2018.

2. Page MJ, McKenzie JE, Bossuyt PM, et al. The PRISMA 2020 statement: an updated guideline for reporting systematic reviews. *BMJ*. 2021;372:n71. doi:10.1136/bmj.n71

3. GRADE Working Group. Welcome to the GRADE working group. Accessed June 6, 2024, <https://www.gradeworkinggroup.org/>

4. RAND. Delphi Method. Accessed June 6, 2024, <https://www.rand.org/topics/delphi-method.html>

5. Gerra G, Zaimovic A, Giusti F, Moi G, Brewer C. Intravenous flumazenil versus oxazepam tapering in the treatment of benzodiazepine withdrawal: a randomized, placebo-controlled study. *Addict Biol*. Oct 2002;7(4):385-95. doi:10.1080/1355621021000005973

6. Petrovic M, Pevernagie D, Mariman A, Van Maele G, Afschrift M. Fast withdrawal from benzodiazepines in geriatric inpatients: a randomised double-blind, placebo-controlled trial. *Eur J Clin Pharmacol*. Jan 2002;57(11):759-64. doi:10.1007/s00228-001-0387-4

7. Lader M FI, Morton S. A comparison of alpidem and placebo in relieving benzodiazepine withdrawal symptoms. *Int Clin Psychopharmacol*. 1993;8(1):31-36.

8. Ashton CH, Rawlins MD, Tyrer SP. A double-blind placebo-controlled study of buspirone in diazepam withdrawal in chronic benzodiazepine users. *Br J Psychiatry*. Aug 1990;157:232-8. doi:10.1192/bjp.157.2.232

9. Lader M, Olajide D. A comparison of buspirone and placebo in relieving benzodiazepine withdrawal symptoms. *J Clin Psychopharmacol*. 1987;7(1):11-15.

10. Morton S, Lader M. Buspirone treatment as an aid to benzodiazepine withdrawal. *J Psychopharmacol*. 1995;9(4):331-335.

11. Udelman HD, Udelman DL. Concurrent use of buspirone in anxious patients during withdrawal from alprazolam therapy. *The Journal of Clinical Psychiatry*. 1990;51:46-50.

12. Rickels K, DeMartinis N, García-España F, Greenblatt DJ, Mandos LA, Rynn M. Imipramine and buspirone in treatment of patients with generalized anxiety disorder who are discontinuing long-term benzodiazepine therapy. *Am J Psychiatry*. Dec 2000;157(12):1973-9. doi:10.1176/appi.ajp.157.12.1973

13. Rynn M, Garcia-Espana F, Greenblatt DJ, Mandos LA, Schweizer E, Rickels K. Imipramine and buspirone in patients with panic disorder who are discontinuing long-term benzodiazepine therapy. *J Clin Psychopharmacol*. Oct 2003;23(5):505-8. doi:10.1097/01.jcp.0000088907.24613.3f

14. Mercier-Guyon C, Chabannes JP, Saviuc P. The role of captodiamine in the withdrawal from long-term benzodiazepine treatment. *Curr Med Res Opin*. Sep 2004;20(9):1347-55. doi:10.1185/030079904125004457

15. Klein E CV, Stolk J, Lenox RH. Alprazolam withdrawal in patients with panic disorder and generalized anxiety disorder: vulnerability and effect of carbamazepine. *Am J Psychiatry*. 1994;151(12):1760-1766.

16. Schweizer E, Rickels K, Case WG, Greenblatt DJ. Carbamazepine treatment in patients discontinuing long-term benzodiazepine therapy. *Arch Gen Psychiatry*. 1991;48(5):448-452.

17. Lemoine P, Kermadi I, Garcia-Acosta S, Garay RP, Dib M. Double-blind, comparative study of cyamemazine vs. bromazepam in the benzodiazepine withdrawal syndrome. *Prog Neuropsychopharmacol Biol Psychiatry*. Jan 2006;30(1):131-7. doi:10.1016/j.pnpbp.2005.08.015

18. Tyrer P, Ferguson B, Hallstrom C, et al. A controlled trial of dothiepin and placebo in treating benzodiazepine withdrawal symptoms. *Br J Psychiatry*. Apr 1996;168(4):457-61. doi:10.1192/bjp.168.4.457

19. Malsch U, Kieser M. Efficacy of kava-kava in the treatment of non-psychotic anxiety, following pretreatment with benzodiazepines. Article. *Psychopharmacology (Berl)*. 2001;157(3):277-283. doi:10.1007/s002130100792

20. Baandrup L, Fasmer OB, Glenthøj BY, Jennum PJ. Circadian rest-activity rhythms during benzodiazepine tapering covered by melatonin versus placebo add-on: data derived from a randomized clinical trial. *BMC Psychiatry*. 2016/10/13 2016;16(1):348. doi:10.1186/s12888-016-1062-8

21. Cardinali DP, Gvozdenovich E, Kaplan MR, et al. A double blind-placebo controlled study on melatonin efficacy to reduce anxiolytic benzodiazepine use in the elderly. Article. *Neuro Endocrinol Lett*. 2002;23(1):55-60.

22. Garfinkel D, Zisapel N, Wainstein J, Laudon M. Facilitation of benzodiazepine discontinuation by melatonin: a new clinical approach. *Arch Intern Med*. 1999;159(8):2456-2460.

23. Lähteenmäki R, Puustinen J, Vahlberg T, et al. Melatonin for sedative withdrawal in older patients with primary insomnia: a randomized double-blind placebo-controlled trial. *Br J Clin Pharmacol*. 2014;77(6):975-985. doi:10.1111/bcp.12294

24. Peles E, Hetzroni T, Bar-Hamburger R, Adelson M, Schreiber S. Melatonin for perceived sleep disturbances associated with benzodiazepine withdrawal among patients in methadone maintenance treatment: a double-blind randomized clinical trial. *Addiction*. Dec 2007;102(12):1947-53. doi:10.1111/j.1360-0443.2007.02007.x

25. Vissers FH, Knipschild PG, Crebolder HF. Is melatonin helpful in stopping the long-term use of hypnotics? A discontinuation trial. *Pharm World Sci*. Dec 2007;29(6):641-6. doi:10.1007/s11096-007-9118-y

26. Romach MK, Kaplan HL, Busto UE, Somer G, Sellers EM. A controlled trial of ondansetron, a 5-HT3 antagonist, in benzodiazepine discontinuation. *J Clin Psychopharmacol*. 1998;18(2):121-131.

27. GlaxoSmithKline. Clinical comparison of paroxetine and placebo on the symptoms emerging during the taper phase of a chronic benzodiazepine treatment, in patients suffering from a variety of anxiety disorders. GSK - Clinical Study Register ([www.gsk-clinicalstudyregister.com](../www.gsk-clinicalstudyregister.com)); 2002.

28. Nakao M, Takeuchi T, Nomura K, Teramoto T, Yano E. Clinical application of paroxetine for tapering benzodiazepine use in non-major-depressive outpatients visiting an internal medicine clinic. *Psychiatry Clin Neurosci*. 2006;60(5):605-610. doi:10.1111/j.1440-1819.2006.01565.x

29. Zitman FG, Couvee JE. Chronic benzodiazepine use in general practice patients with depression: an evaluation of controlled treatment and taper-off: report on behalf of the Dutch Chronic Benzodiazepine Working Group. *Br J Psychiatry*. Apr 2001;178:317-24. doi:10.1192/bjp.178.4.317

30. Hadley SJ, Mandel FS, Schweizer E. Switching from long-term benzodiazepine therapy to pregabalin in patients with generalized anxiety disorder: a double-blind, placebo-controlled trial. *J Psychopharmacol*. Apr 2012;26(4):461-70. doi:10.1177/0269881111405360

31. Schweizer E, Case WG, Garcia-Espana F, Rickels K, Greenblatt DJ. Progesterone co-administration in patients discontinuing long-term benzodiazepine therapy: effects on withdrawal severity and taper outcome. *Psychopharmacology*. 1995;117:424-429.

32. Rickels K, Schweizer E, Garcia Espana F, Case G, DeMartinis N, Greenblatt D. Trazodone and valproate in patients discontinuing long-term benzodiazepine therapy: effects on withdrawal symptoms and taper outcome. *Psychopharmacology (Berl)*. 1999;141:1-5.

33. Vorma H, Katila H. Effect of valproate on benzodiazepine withdrawal severity in opioid-dependent subjects: a pilot study. *Heroin Addict Relat Clin Probl*. 2011;13(1):15-20.

34. Pat-Horenczyk R, Hacohen D, Herer P, Lavie P. The effects of substituting zopiclone in withdrawal from chronic use of benzodiazepine hypnotics. *Psychopharmacology (Berl)*. 1998;140(4):450-457.

35. Elliott L, Glenday J, Freeman L, et al. Reducing diazepam prescribing for illicit drug users: a randomised control study. *Drug Alcohol Rev*. Jan 2005;24(1):25-31. doi:10.1080/09595230500125138

36. Gosselin P, Ladouceur R, Morin CM, Dugas MJ, Baillargeon L. Benzodiazepine discontinuation among adults with GAD: a randomized trial of cognitive-behavioral therapy. *J Consult Clin Psychol*. 2006;74(5):908-919. doi:10.1037/0022-006X.74.5.908

37. O'Connor K, Marchand A, Brousseau L, et al. Cognitive-behavioural, pharmacological and psychosocial predictors of outcome during tapered discontinuation of benzodiazepine. *Clin Psychol Psychother*. 2008;15(1):1-14. doi:10.1002/cpp.556

38. Otto MW, Pollack MH, Sachs GS, Reiter SR, Meltzer-Brody S, Rosenbaum JF. Discontinuation of benzodiazepine treatment: efficacy of cognitive-behavioral therapy. *Am J Psychiatry*. 1993;150(10):1485-1490.

39. Otto MW, McHugh RK, Simon NM, Farach FJ, Worthington JJ, Pollack MH. Efficacy of CBT for benzodiazepine discontinuation in patients with panic disorder: further evaluation. *Behav Res Ther*. 2010;48(8):720-727. doi:10.1016/j.brat.2010.04.002

40. Oude Voshaar RC, Gorgels WJ, Mol AJ, et al. Tapering off long-term benzodiazepine use with or without group cognitive-behavioural therapy: three-condition, randomised controlled trial. *Br J Psychiatry*. 2003;182:498-504. doi:10.1192/bjp.182.6.498

41. Spiegel DA, Bruce TJ, Gregg SF, Nuzzarello A. Does cognitive behavior therapy assist slow-taper alprazolam discontinuation in panic disorder? *Am J Psychiatry*. 1994;151(6):876-881.

42. Vorma H, Naukkarinen H, Sarna S, Kuoppasalmi K. Treatment of out-patients with complicated benzodiazepine dependence: comparison of two approaches. *Addiction*. 2002;97(7):851-859.

43. Baillargeon L, Landreville P, Verreault R, Beauchemin JP, Grégoire JP, Morin CM. Discontinuation of benzodiazepines among older insomniac adults treated with cognitive-behavioural therapy combined with gradual tapering: a randomized trial. *CMAJ*. 2003;169(10):1015-1020.

44. Belleville G, Guay C, Guay B, Morin CM. Hypnotic taper with or without self-help treatment of insomnia: a randomized clinical trial. *J Consult Clin Psychol*. Apr 2007;75(2):325-35. doi:10.1037/0022-006x.75.2.325

45. Coteur K, Henrard G, Schoenmakers B, et al. Blended care to discontinue benzodiazepine receptor agonists use in patients with chronic insomnia disorder: a pragmatic cluster randomized controlled trial in primary care. *Sleep*. 2023;46(4):zsac278. doi:10.1093/sleep/zsac278

46. Lui E, Wintemute K, Muraca M, et al. Pharmacist-led sedative-hypnotic deprescribing in team-based primary care practice. *Can Pharm J (Ott)*. Jul-Aug 2021;154(4):278-284. doi:10.1177/17151635211014918

47. Morin CM, Bastien C, Guay B, Radouco-Thomas M, Leblanc J, Vallières A. Randomized clinical trial of supervised tapering and cognitive behavior therapy to facilitate benzodiazepine discontinuation in older adults with chronic insomnia. *Am J Psychiatry*. 2004;161(2):332-342. doi:10.1176/appi.ajp.161.2.332

48. Yeung WF, Chung KF, Zhang ZJ, et al. Electroacupuncture for tapering off long-term benzodiazepine use: a randomized controlled trial. Article. *J Psychiatr Res*. 2019;109:59-67. doi:10.1016/j.jpsychires.2018.11.015

49. Barros VV, Opaleye ES, Demarzo M, et al. Effects of mindfulness-based relapse prevention on the chronic use of hypnotics in treatment-seeking women with insomnia: a randomized controlled trial. *Int J Behav Med*. 2022;29(3):266-277. doi:10.1007/s12529-021-10002-4

50. Elsesser K, Sartory G, Maurer J. The efficacy of complaints management training in facilitating benzodiazepine withdrawal. *Behav Res Ther*. 1996;34(2):149-156. doi:10.1016/0005-7967(95)00051-8

51. Gilbert A, Owen N, Innes JM, Sansom L. Trial of an intervention to reduce chronic benzodiazepine use among residents of aged-care accommodation. *Aust N Z J Med*. Aug 1993;23(4):343-7. doi:10.1111/j.1445-5994.1993.tb01433.x

52. Tannenbaum C, Martin P, Tamblyn R, Benedetti A, Ahmed S. Reduction of inappropriate benzodiazepine prescriptions among older adults through direct patient education: the EMPOWER cluster randomized trial. *JAMA Intern Med*. 2014;174(6):890-898. doi:10.1001/jamainternmed.2014.949

53. Gorgels WJ, Oude Voshaar RC, Mol AJ, et al. Discontinuation of long-term benzodiazepine use by sending a letter to users in family practice: a prospective controlled intervention study. *Drug Alcohol Depend*. Apr 4 2005;78(1):49-56. doi:10.1016/j.drugalcdep.2004.09.001

54. Heather N, Bowie A, Ashton H, et al. Randomised controlled trial of two brief interventions against long-term benzodiazepine use: outcome of intervention. *Addict Res Theory*. 2004/04/01 2004;12(2):141-154. doi:10.1080/1606635310001634528

55. Ten Wolde GB, Dijkstra A, van Empelen P, van den Hout W, Neven AK, Zitman F. Long-term effectiveness of computer-generated tailored patient education on benzodiazepines: a randomized controlled trial. *Addiction*. Apr 2008;103(4):662-70. doi:10.1111/j.1360-0443.2008.02141.x

56. Vicens C, Fiol F, Llobera J, et al. Withdrawal from long-term benzodiazepine use: randomised trial in family practice. *Br J Gen Pract*. Dec 2006;56(533):958-63.

57. Vicens C, Bejarano F, Sempere E, et al. Comparative efficacy of two interventions to discontinue long-term benzodiazepine use: cluster randomised controlled trial in primary care. Article. *Br J Psychiatry*. 2014;204(6):471-479. doi:10.1192/bjp.bp.113.134650

58. MacDonald T, Gallo AT, Basso-Hulse G, Bennett KS, Hulse GK. A double-blind randomised crossover trial of low-dose flumazenil for benzodiazepine withdrawal: A proof of concept. Article. *Drug Alcohol Depend*. 2022;236doi:10.1016/j.drugalcdep.2022.109501

59. Gopalan P, Moses-Kolko E, Valpey R, Shenai N, Smith E. Benzodiazepine withdrawal in pregnant women with opioid use disorders: an observational study of current clinical practices at a tertiary obstetrical hospital. *Gen Hosp Psychiatry*. 2019;57:29-33. doi:10.1016/j.genhosppsych.2018.12.005

60. McGregor C, Machin A, White JM. In-patient benzodiazepine withdrawal: comparison of fixed and symptom-triggered taper methods. *Drug Alcohol Rev*. Jun 2003;22(2):175-80. doi:10.1080/09595230100100615

61. Curran HV, Collins R, Fletcher S, Kee SC, Woods B, Iliffe S. Older adults and withdrawal from benzodiazepine hypnotics in general practice: effects on cognitive function, sleep, mood and quality of life. *Psychol Med*. 2003;33(7):1223-1237. doi:10.1017/s0033291703008213

62. Shea BJ, Reeves BC, Wells G, et al. AMSTAR 2: a critical appraisal tool for systematic reviews that include randomised or non-randomised studies of healthcare interventions, or both. *BMJ*. 2017;358:j4008. doi:10.1136/bmj.j4008

63. Sterne JAC, Savovic J, Page MJ, et al. RoB 2: a revised tool for assessing risk of bias in randomised trials. *BMJ*. 2019;366:l4898. doi:10.1136/bmj.l4898

64. National Heart Lung and Blood Institute. Quality Assessment Tool for Observational Cohort and Cross-Sectional Studies. 2021.

65. *Review Manager*. Cochrane; 2024. revman.cochrane.org

66. Higgins JPT, Thomas J, Chandler J, et al, eds. *Cochrane Handbook for Systematic Reviews of Interventions version 6.4*. Cochrane; 2023. [www.training.cochrane.org/handbook](../www.training.cochrane.org/handbook)

67. *GRADEpro GDT: GRADEpro Guideline Development Tool*. 2024. gradepro.org

# S4. Disclosures of Interest

## I. 2024 Clinical Guideline Committee Member Relationships with Industry and Other Entities

| **Clinical Guideline Committee Member** | **Employment** | **Consultant** | **Speakers Bureau** | **Ownership/ Partnership/ Principal** | **Institutional, Organizational, or Other Financial Benefit** | **Research** |
| --- | --- | --- | --- | --- | --- | --- |
| Emily Brunner, MD, DFASAM (*Chair*) | Gateway; Hazelden Betty Ford; Recovery Hope; Henry Ford Hospital (spouse) | None | None | None | None | None |
| Chwen-Yuen A. Chen, MD, FACP, FASAM | Stanford University; Private Practice | Anonymous Health*; Expert Witness* | None | Private Practice** | None | None |
| Tracy Klein, PhD, FNP, ARNP, FAANP, FRE, FAAN | Washington State University | Expert Witness* | None | None | Oregon PDMP Advisory Committee | None |
| Donovan Maust, MD, MS | University of Michigan | Expert Witness**  Predictably Human; Consultant*^ | None | None  Predictably Human; Stock Ownership*^ | None | $2.9 million NIH grant on BZD related harms among older adults (2018 – 2023) |
| Maryann Mazer-Amirshahi, PharmD, MD, PhD, MPH, FASAM | MedStar Washington Hospital Center; National Capital Poison Center; Georgetown University | Expert Witness*; FDA Advisory Panels* | None | None | None | None |
| Marcia Mecca, MD | VA Connecticut | None | None | None | None | None |
| Deanna Najera, MPAS, MS, PA-C, DFAAPA | Medstar Emergency Physicians; Carroll County Health Department; TrueNorth Wellness Services; Cinda Liggon, MD, LLC; USACS | None | PA Foundation*; AAPA*; Maryland Academy of Physician Assistants*; Pennsylvania Society of Physician Associates* | None | None | None |
| Chinyere Ogbonna, MD, MPH | Kaiser Permanente San Jose | None | None | None | None | None |
| Kiran F. Rajneesh, MD, MS, FAAN | The Ohio State University | Merck Pharmaceuticals* | None | None | None | None |
| Elizabeth Roll, MD | Yukon Kuskokwim Health Corporation | None | None | None | None | None |
| Amy E. Sanders, MD, MS, MPhil, FAAN | Sunday Health | Ionis Pharmaceuticals* | None | None | None | None |
| Brett Snodgrass, DNP, FNP-C, ACHPN, FAANP | Baptist Memorial Health Care | None | Salix Pharmaceuticals** | None | None | None |
| Amy Vandenberg, PharmD, BCPP | University of Michigan College of Pharmacy | Expert Witness* | None | None | None | None |
| Tricia Wright, MD, MS, FACOG, DFASAM | University of California San Francisco | None | None | None | None | None |
| The above table presents relationships of the Clinical Guideline Committee during the past 24 months with industry and other entities. These relationships are current as of the completion of this document and may not necessarily reflect relationships at the time of this document’s publication. A relationship is considered to be significant if the individual receives compensation valued at $5,000 or more. A relationship is considered to be modest if it is less than significant under the preceding definition. A relationship is considered to be unpaid if the individual does not receive monetary reimbursement.  ** Indicates significant relationship. * Indicates modest relationship. ^Occurred after the start of the project; not reviewed | | | | | | |

## II. 2024 ASAM Quality Improvement Council Relationships with Industry and Other Entities

| **Quality Improvement Council Member** | **Employment** | **Consultant** | **Speakers Bureau** | **Ownership/ Partnership/ Principal** | **Institutional, Organizational, or Other Financial Benefit** | **Research** |
| --- | --- | --- | --- | --- | --- | --- |
| Itai Danovitch, MD, MBA, FAPA, DFASAM | Cedars-Sinai Medical Center | Expert Witness** | None | None | Bexon Biomedical Board of Directors*; Workit Health*; California Mental Health Services Commissioner | None |
| Kenneth I. Freedman, MD, MS, MBA, FACP, AGAF, DFASAM | Aetna/CVS Health; The Recovery Research Network | None | None | None | National Quality Forum | None |
| Michael P. Frost, MD, DFASAM, FACP | Wayspring; Pocket Naloxone Corp; Frost Medical Group, LLC | Accord Healthcare UK* | Braeburn Pharmaceuticals* | Frost Medical Group, LLC** | None | None |
| R. Jeffrey Goldsmith, MD, DLFAPA, DFASAM | None | None | None | Bristol-Myers Sqiubb**; Gilead Sciences Inc**; Merck and Co, Inc**; Pfizer Inc**; Sanofi ADR** | Windhorse Zen Community Board Member* | None |
| Margaret A. Jarvis, MD, DFASAM | Geisinger | ASAM**; Expert Witness** | None | None | PA Governor’s Behavioral Health Council; American Board of Preventive Medicine Exam Subcommittee** | None |
| Navdeep Kang, PsyD | Acadia Healthcare | Bonfire Analytics* | None | Brightview Health** | Talbert House Board of Trustees | None |
| Tiffany Y. Lu, MD, MS | Albert Einstein College of Medicine | None | None | None | None | None |
| Tami Mark, PhD, MBA | RTI International | None | None | None | None | None |
| Stephen Martin, MD, FASAM | Boulder Care; Greylock Recovery | None | None | Boulder Care | None | None |
| Cynthia Vuittonet, MD, FASAM | Central Jersey Medical Center | Expert Witness** | None | None | None | None |
| Melissa B. Weimer, DO, MCR, FASAM | Yale School of Medicine; Medical Legal Consulting; St. Peters Health Partners, Yale New Haven Hospital; PCSS-MAUS | CVS Health | None | None | ASAM | None |
| The above table presents relationships of the ASAM Quality Improvement Council during the past 12 months with industry and other entities that were determined to be relevant to this document. These relationships are current as of the completion of this document and may not necessarily reflect relationships at the time of this document’s publication. A relationship is considered to be significant if the individual receives compensation which includes cash, shares, and/or anything else of value including direct ownership of shares, stock, stock options or other interest of 5% more of an entity or valued at $10,000 or more (excluding mutual funds), whichever is greater. A relationship is considered to be modest if it is less than significant under the preceding definition. A relationship is considered to be unpaid if the individual does not receive monetary reimbursement.  ** Indicates significant relationship. * Indicates modest relationship. | | | | | | |

## III. 2024 ASAM Board of Directors Relationships with Industry and Other Entities

| **Board Member** | **Employment** | **Consultant** | **Speakers Bureau** | **Ownership/ Partnership/ Principal** | **Institutional, Organizational, or Other Financial Benefit** | **Research** |
| --- | --- | --- | --- | --- | --- | --- |
| Anika Alvanzo, MD, MS, FACP, DFASAM | Health Management Associates; Absolute Care | Uzima Consulting Group, LLC** | None | None | None | None |
| Keyghobad Farid Araki, MD, FRCPC, ABAM, FASAM | Centre for Addiction and Mental Health | None | None | None | None | None |
| Nicholas Athanasiou, MD, MBA, DFASAM | University of California Los Angeles | None | None | None | None | None |
| Emily Brunner, MD, DFASAM | Gateway; Hazelden Betty Ford; Recovery Hope; Henry Ford Hospital (spouse) | None | None | None | None | None |
| Megan Buresh, MD, DFASAM | Johns Hopkins University School of Medicine | None | None | None | American Journal of Medicine* | None |
| Itai Danovitch, MD, MBA, FAPA, DFASAM | Cedars-Sinai Medical Center | Expert Witness** | None | None | Bexon Biomedical Board of Directors*; Workit Health*; California Mental Health Services Commissioner | None |
| Alta DeRoo, MD, MBA, FACOG, DFASAM | Hazelden Betty Ford Foundation | None | None | None | None | None |
| Michael Fingerhood, MD, FACP, DFASAM | Johns Hopkins University | None | None | None | American Academy of HIV Medicine | None |
| Kenneth I. Freedman, MD, MS, MBA, FACP, AGAF, DFASAM | Aetna/CVS Health; The Recovery Research Network | None | None | None | National Quality Forum | None |
| William F. Haning III, MD, DLFAPA, DFASAM | University of Hawai’i John A. Burns School of Medicine | Hawai’i State Department of Education (Spouse) | None | None | Honolulu Police Commission (Spouse) | None |
| Brian Hurley, MD, MBA, FAPA, DFASAM | Los Angeles County Department of Public Health; Private Practice; Centers for Care Innovation, PsyBAR; Camden Center | None | None | None | Frank Foundation Board of Directors | None |
| Teresa Jackson, MD, DFASAM | Lakeside-Milam Recovery Center | None | None | None | None | None |
| Margaret A. E. Jarvis, MD, DFASAM | Geisinger | ASAM**; Expert Witness** | None | None | PA Governor’s Behavioral Health Council; American Board of Preventive Medicine Exam Subcommittee** | None |
| Christina E. Jones, MD, FASAM | Teleleaf, LLC | None | None | None | None | None |
| Lori D. Karan, MD, FACP, DFASAM | VA Loma Linda Healthcare Center; Loma Linda University Health Education Consortium | None | None | None | None | None |
| Audrey M. Kern, MD, DFASAM | DynamiCare Health | None | None | None | New Hampshire Healthy Families Board of Directors* | None |
| Marla D. Kushner, DO, FACOFP, FAOAAM, FSAHM, DFASAM | Marla D. Kushner, DO, SC; Bicycle Health | None | None | Marla D. Kushner, DO, SC | None | None |
| Nicole Labor, DO, FASAM | Optimus Transformative Medicine, LLC; Laborhood Change Project, Inc; OneEighty, Inc; Interval Brotherhood Homes, Inc; Esper Treatment Center | None | None | None | None | None |
| James P. Murphy, MD, DFASAM | Murphy Pain Center | None | None | Murphy Pain Center** | Kentucky Harm Reduction Coalition Board of Directors; University of Louisville School of Medicine | None |
| Cara A. Poland, MD, MEd, FACP, DFASAM | Michigan State University College of Human Medicine | None | None | None | None | None |
| Shawn Ryan, MD, MBA, FASAM | Brightview Health | Dynamicare* | None | Brightview Health* | None | None |
| Kelly S. Ramsey, MD, MPH, MA, FACP, DFASAM | Kelly S. Ramsey Consulting, LLC.; Case Western Reserve University | None | None | None | None | None |
| Surita Rao, MD, FASAM | University of Connecticut School of Medicine | None | None | None | None | None |
| Stephen M. Taylor, MD, MPH, DFAPA, DFASAM | Stephen M. Taylor, MD, PC; Pathway Healthcare Services, LLC | None | None | Stephen M. Taylor, MD, PC** | Medical Review Officer Certification Council Board of Directors; Addiction Prevention Coalition Board of Directors | None |
| Michael F. Weaver, MD, DFASAM | University of Texas Health Science Center at Houston and Center for Neurobehavioral Research on Addiction | None | None | None | American Board of Preventive Medicine | None |
| Timothy Wiegand, MD, FACMT, FAACT, DFASAM | University of Rochester Medical Center; Huther Doyle; Helio Health/Syracuse Behavioral Health; UpToDate; Aids Institute Department of Health | Medical legal Consulting** | None | None | ACMT; Medical Toxicology Foundation | None |
| Aleksandra E. Zgierska, MD, PhD, DFASAM | Pennsylvania State University | Pennsylvania Medicaid* | None | None | American Academy of Pain Medicine* | National Institutes of Health; National Institute on Drug Abuse |
| The above table presents relationships of the ASAM Board of Directors during the past 12 months with industry and other entities that were determined to be relevant to this document. These relationships are current as of the completion of this document and may not necessarily reflect relationships at the time of this document’s publication. A relationship is considered to be significant if the individual receives compensation which includes cash, shares, and/or anything else of value including direct ownership of shares, stock, stock options or other interest of 5% more of an entity or valued at $10,000 or more (excluding mutual funds), whichever is greater. A relationship is considered to be modest if it is less than significant under the preceding definition. A relationship is considered to be unpaid if the individual does not receive monetary reimbursement.  ** Indicates significant relationship. * Indicates modest relationship. | | | | | | |

# S5. Evidence to Decision Tables

## EtD Table 1. Taper (+/- Placebo) Compared to Abrupt Cessation (+/- Placebo) for Benzodiazepine Discontinuation

### Brief Evidence Summary

This EtD table supports Key Question 1. The systematic review identified two RCTs with 70 participants that compared a gradual BZD taper to abrupt cessation. Both RCTs had an unclear risk of bias (Gerra et al [2002]; Petrovic et al [2002]). The gradual taper schedules used were relatively rapid, lasting only 7–8 days. There was no difference in the rate of complete BZD discontinuation, return to BZD use after a period of discontinuation, delirium, or study completion between groups. However, patients undergoing a gradual taper reported significantly less severe BZD withdrawal and insomnia symptoms after 4 days (ie, mid-taper) and up to 4 weeks compared to patients who abruptly stopped their BZD use. Patients undergoing a gradual taper also reported significantly less intense BZD cravings after 4 days (ie, mid-taper), but this effect was no longer detected after 7 days (ie, taper end).

### Summary of Findings Table

| **Certainty Assessment** | | | | | | | **№ of Patients** | | **Effect** | | **Certainty** | **Importance** |
| --- | --- | --- | --- | --- | --- | --- | --- | --- | --- | --- | --- | --- |
| **№ of Studies** | **Study Design** | **Risk of Bias** | **Inconsistency** | **Indirectness** | **Imprecision** | **Other Consider-ations** | **Taper** | **Abrupt Cessation** | **Relative (95% CI)** | **Absolute (95% CI)** |  |  |
| **BZD discontinuation @ taper end (assessed with: patient self-report)** | | | | | | | | | | | | |
| 1^1^ | randomized trials | not serious | not serious | not serious | very serious^a^ | none | 19/20 (95.0%) | 20/20 (100.0%) | **RR 0.95** (0.83 to 1.09) | **5 fewer per 100** (from 17 fewer to 9 more) | ⨁⨁◯◯ Low | CRITICAL |
| **BZD discontinuation @ 1-week follow-up (assessed with: patient self-report)** | | | | | | | | | | | | |
| 1^1^ | randomized trials | not serious | not serious | not serious | very serious^a^ | none | 18/20 (90.0%) | 17/20 (85.0%) | **RR 1.06** (0.84 to 1.34) | **5 more per 100** (from 14 fewer to 29 more) | ⨁⨁◯◯ Low | CRITICAL |
| **BZD discontinuation @ 3-week follow-up (assessed with: patient self-report)** | | | | | | | | | | | | |
| 1^1^ | randomized trials | not serious | not serious | not serious | very serious^a^ | none | 16/20 (80.0%) | 10/20 (50.0%) | **RR 1.60** (0.98 to 2.61) | **30 more per 100** (from 1 fewer to 81 more) | ⨁⨁◯◯ Low | CRITICAL |
| **Return to BZD use after discontinuation @ 12-month follow-up (assessed with: general practitioner report)** | | | | | | | | | | | | |
| 1^1^ | randomized trials | not serious | not serious | not serious | very serious^a^ | none | 8/16 (50.0%) | 6/10 (60.0%) | **RR 0.83** (0.41 to 1.69) | **10 fewer per 100** (from 35 fewer to 41 more) | ⨁⨁◯◯ Low | CRITICAL |
| **Experienced delirium during taper** | | | | | | | | | | | | |
| 1^1^ | randomized trials | not serious | not serious | not serious | very serious^a^ | none | 0/20 (0.0%) | 2/20 (10.0%) | **Peto OR 0.13** (0.01 to 2.13) | **10 fewer per 100** (from 25 fewer to 5 more)^b^ | ⨁⨁◯◯ Low | CRITICAL |
| **Withdrawal severity score @ mid-taper (assessed with: BWSQ; self-report study scale, higher = more severe withdrawal)** | | | | | | | | | | | | |
| 2^1,2^ | randomized trials | not serious | not serious | not serious | serious^c^ | none | 39 | 30 | — | SMD **0.72 SD lower** (1.22 lower to 0.22 lower) | ⨁⨁⨁◯ Moderate | CRITICAL |
| **Withdrawal severity score @ mid-taper (assessed with: observer-rated study scale, score range 0–4, higher = more severe withdrawal)** | | | | | | | | | | | | |
| 1^2^ | randomized trials | serious^d^ | not serious | not serious | very serious^a^ | none | 20 | 10 | — | MD **0.44 lower** (1.32 lower to 0.45 higher) | ⨁⨁◯◯ Low | CRITICAL |
| **Withdrawal severity score @ taper end (assessed with: BWSQ; self-report study scale, higher = more severe withdrawal)** | | | | | | | | | | | | |
| 2^1,2^ | randomized trials | not serious | serious^e^ | not serious | serious^c^ | none | 39 | 30 | — | SMD **0.54 SD lower** (1.05 lower to 0.04 lower) | ⨁⨁◯◯ Low | CRITICAL |
| **Withdrawal severity score @ taper end (assessed with: observer-rated study scale, score range 0–4, higher = more severe withdrawal)** | | | | | | | | | | | | |
| 1^2^ | randomized trials | serious^d^ | not serious | not serious | very serious^a^ | none | 20 | 10 | — | MD **0.22 higher** (0.27 lower to 0.7 higher) | ⨁⨁◯◯ Low | CRITICAL |
| **Withdrawal severity score @ 1-week follow-up (assessed with: BWSQ)** | | | | | | | | | | | | |
| 1^1^ | randomized trials | serious^f^ | not serious | not serious | serious^c^ | none | 18 | 17 | — | MD **1.3 lower** (1.69 lower to 0.91 lower) | ⨁⨁◯◯ Low | CRITICAL |
| **Withdrawal severity score @ 3-week follow-up (assessed with: BWSQ)** | | | | | | | | | | | | |
| 1^1^ | randomized trials | serious^f^ | not serious | not serious | serious^c^ | none | 16 | 10 | — | MD **1.88 lower** (2.37 lower to 1.39 lower) | ⨁⨁◯◯ Low | CRITICAL |
| **Dropout** | | | | | | | | | | | | |
| 2^1,2^ | randomized trials | not serious | not serious | not serious | very serious^a^ | none | 1/20 (5.0%) | 0/20 (0.0%) | **RD -0.03** (-0.07 to 0.13) | **30 more per 1,000** (from 70 fewer to 130 more)^b^ | ⨁⨁◯◯ Low | IMPORTANT |

**BWSQ:** Benzodiazepine Withdrawal Symptom Questionnaire, score range 0-40, higher = more severe withdrawal symptoms, self-report; **BZD:** benzodiazepine; **CI:** confidence interval; **MD:** mean difference; **OR:** odds ratio; **RD:** risk difference; **RR:** risk ratio; **SD:** standard deviation; **SMD:** standardized mean difference

**GRADE Working Group Grades of Evidence**

High quality: Further research is very unlikely to change our confidence in the estimate of effect.

Moderate quality: Further research is likely to have an important impact on our confidence in the estimate of effect and may change the estimate.

Low quality: Further research is very likely to have an important impact on our confidence in the estimate of effect and is likely to change the estimate.

Very low quality: We are very uncertain about the estimate.

**Explanations**

- 1. Small sample size (n = <100) and 95% CI crosses the line of null effect.
  2. Absolute effect calculated from the risk difference (RD) due to zero events in one or both arms.
  3. Small number of participants (n = <100)
  4. High risk of performance and detection bias from lack of personnel and assessor blinding for a majority of participants.
  5. Significant heterogeneity (I^2^ = 77%, p = 0.04).
  6. High risk of attrition bias. No follow-up data collected from dropouts. Dropout higher in the abrupt cessation group.

### Question

| **Should Taper or Abrupt Cessation be used for BZD discontinuation?** | |
| --- | --- |
| **POPULATION** | Patients discontinuing long-term BZD use |
| **INTERVENTION** | BZD taper (with or without placebo) |
| **COMPARISON** | Abrupt cessation of BZD (with or without placebo) |
| **MAIN OUTCOMES** | BZD discontinuation (patient self-report); return to BZD use after discontinuation (reported by patient’s general practitioner); experienced delirium during taper; withdrawal symptom severity score; dropout |
| **SETTING** | Any clinical setting where BZD tapering occurs |
| **PERSPECTIVE** | Individual-level |
| **CONFLICT OF INTERESTS** | None identified |

### Assessment

| **Problem**  Is the problem a priority? | | |
| --- | --- | --- |
| Judgment | Research Evidence | Additional Considerations |
| ○ No  ○ Probably no  ○ Probably yes  ● Yes  ○ Varies  ○ Unknown |  |  |
| **Desirable Effects**  How substantial are the desirable anticipated effects? | | |
| Judgment | Research Evidence | Additional Considerations |
| ○ Trivial  ● Small  ○ Moderate  ○ Large  ○ Varies  ○ Unknown | See [Summary of Findings Table](#_Summary_of_Findings) | Based on their experience, the CGC agreed that, in general, a gradual taper is beneficial compared to abrupt BZD cessation. However, a taper over only 1 week may be too rapid to see a significant benefit over abrupt cessation. Also, a taper without other supportive adjuncts may not be sufficient. |
| **Undesirable Effects**  How substantial are the undesirable anticipated effects? | | |
| Judgment | Research Evidence | Additional Considerations |
| ○ Trivial  ● Small  ○ Moderate  ○ Large  ○ Varies  ○ Unknown | One participant from the taper group dropped out of the study early. Two out of seventy participants experienced delirium, both following abrupt cessation of BZDs. Although the incidence of delirium was low (2.9%), the harm associated with delirium severe enough to warrant consideration. | Neither study reported the incidence of seizures. The CGC pointed out that no institutional review board of the recent era would allow randomized abrupt BZD discontinuation in patients at risk for seizures. Gerra et al (2002) did not include any post-taper follow-up. |
| **Certainty of Evidence**  What is the overall certainty of the evidence of effects? | | |
| Judgment | Research Evidence | Additional Considerations |
| ○ Very low  ● Low  ○ Moderate  ○ High  ○ No included studies | \| **Outcomes** \| **Importance** \| **Certainty of the Evidence (GRADE)** \| \| --- \| --- \| --- \| \| BZD discontinuation @ taper end  (assessed with: patient self-report) \| CRITICAL \| ⨁⨁◯◯ Low^a^ \| \| BZD discontinuation @ 1-week follow-up  (assessed with: patient self-report) \| CRITICAL \| ⨁⨁◯◯ Low^a^ \| \| BZD discontinuation @ 3-week follow-up  (assessed with: patient self-report) \| CRITICAL \| ⨁⨁◯◯ Low^a^ \| \| Return to BZD use after discontinuation @ 12-month follow-up  (assessed with: general practitioner report) \| CRITICAL \| ⨁⨁◯◯ Low^a^ \| \| Withdrawal severity score @ mid-taper  (assessed with: BWSQ; self-report study scale) \| CRITICAL \| ⨁⨁⨁◯ Moderate^b^ \| \| Withdrawal severity score @ mid-taper  (assessed with: observer-rated study scale) \| CRITICAL \| ⨁⨁◯◯ Low^a,c^ \| \| Withdrawal severity score @ taper end  (assessed with: BWSQ; self-report study scale) \| CRITICAL \| ⨁⨁◯◯ Low^b,d^ \| \| Withdrawal severity score @ taper end  (assessed with: observer-rated study scale) \| CRITICAL \| ⨁⨁◯◯ Low^a,c^ \| \| Withdrawal severity score @ 1-week follow-up  (assessed with: BWSQ) \| CRITICAL \| ⨁⨁◯◯ Low^b,e^ \| \| Withdrawal severity score @ 3-week follow-up  (assessed with: BWSQ) \| CRITICAL \| ⨁⨁◯◯ Low^b,e^ \| \| Experienced delirium during taper \| CRITICAL \| ⨁⨁◯◯ Low^a^ \| \| Dropout \| IMPORTANT \| ⨁⨁◯◯ Low^a^ \|  1. Small sample size (n = <100) and 95% CI crosses the line of null effect. 2. Small number of participants (n = <100) 3. High risk of performance and detection bias from a lack of personnel and assessor blinding for most participants. 4. Significant heterogeneity (I^2^ = 77%, p = 0.04). 5. High risk of attrition bias. No follow-up data collected from dropouts. Dropouts higher in the abrupt cessation group. |  |
| **Values**  Is there important uncertainty about or variability in how much people value the main outcomes? | | |
| Judgment | Research Evidence | Additional Considerations |
| ○ Important uncertainty or variability  ○ Possibly important uncertainty or variability  ● Probably no important uncertainty or variability  ○ No important uncertainty or variability |  |  |
| **Balance of Effects**  Does the balance between desirable and undesirable effects favor the intervention or the comparison? | | |
| Judgment | Research Evidence | Additional Considerations |
| ○ Favors the comparison  ○ Probably favors the comparison  ○ Does not favor either the intervention or the comparison  ● Probably favors the intervention  ○ Favors the intervention  ○ Varies  ○ Unknown |  |  |
| **Resources Required**  How large are the resource requirements (ie, costs)? | | |
| Judgment | Research Evidence | Additional Considerations |
| ○ Large costs  ○ Moderate costs  ○ Negligible costs and savings  ○ Moderate savings  ○ Large savings  ● Varies  ○ Unknown |  |  |
| **Cost Effectiveness**  Does the cost-effectiveness of the intervention favor the intervention or the comparison? | | |
| Judgment | Research Evidence | Additional Considerations |
| ○ Favors the comparison  ○ Probably favors the comparison  ○ Does not favor either the intervention or the comparison  ○ Probably favors the intervention  ○ Favors the intervention  ● Varies  ○ No included studies |  |  |
| **Acceptability**  Is the intervention acceptable to key stakeholders? | | |
| Judgment | Research Evidence | Additional Considerations |
| ○ No  ○ Probably no  ○ Probably yes  ● Yes  ○ Varies  ○ Unknown | Providers and key stakeholders are against abrupt cessation. The CGC agreed that the interventions included in the research evidence do not reflect a patient-centered process nor clinical practice due to the lack of patient input and sense of control. |  |
| **Feasibility**  Is the intervention feasible to implement? | | |
| Judgment | Research Evidence | Additional Considerations |
| ○ No  ○ Probably no  ○ Probably yes  ● Yes  ○ Varies  ○ Unknown |  |  |

### Summary of Judgments

|  | **JUDGMENT** | | | | | | |
| --- | --- | --- | --- | --- | --- | --- | --- |
| **PROBLEM** | No | Probably no | Probably yes | **Yes** |  | Varies | Unknown |
| **DESIRABLE EFFECTS** | Trivial | **Small** | Moderate | Large |  | Varies | Unknown |
| **UNDESIRABLE EFFECTS** | Trivial | **Small** | Moderate | Large |  | Varies | Unknown |
| **CERTAINTY OF EVIDENCE** | Very low | **Low** | Moderate | High |  |  | No included studies |
| **VALUES** | Important uncertainty or variability | Possibly important uncertainty or variability | **Probably no important uncertainty or variability** | No important uncertainty or variability |  |  |  |
| **BALANCE OF EFFECTS** | Favors the comparison | Probably favors the comparison | Does not favor either the intervention or the comparison | **Probably favors the intervention** | Favors the intervention | Varies | Unknown |
| **RESOURCES REQUIRED** | Large costs | Moderate costs | Negligible costs and savings | Moderate savings | Large savings | **Varies** | Unknown |
| **COST EFFECTIVENESS** | Favors the comparison | Probably favors the comparison | Does not favor either the intervention or the comparison | Probably favors the intervention | Favors the intervention | **Varies** | No included studies |
| **ACCEPTABILITY** | No | Probably no | Probably yes | **Yes** |  | Varies | Unknown |
| **FEASIBILITY** | No | Probably no | Probably yes | **Yes** |  | Varies | Unknown |

### Type of Recommendation

| Strong recommendation against the intervention | Conditional recommendation against the intervention | Conditional recommendation for either the intervention or the comparison | Conditional recommendation for the intervention | **Strong recommendation for the intervention** |
| --- | --- | --- | --- | --- |
| ○ | ○ | ○ | ○ | ● |

### Conclusions

| **Recommendation** |
| --- |
| **Recommendation 2:** Clinicians should avoid abruptly discontinuing BZD medication in patients who are likely to be physically dependent on BZDs and at risk for BZD withdrawal (see [Table 3](#Table3); Low Certainty, Strong Recommendation).  **Recommendation 2a:** Tapering is indicated for patients who are likely to be physically dependent when the risks of BZD medication outweigh the benefits (Low Certainty, Strong Recommendation). |
| **Justification** |
| The evidence of treatment effect is uncertain due to the small size and risk of bias in the studies evaluated. Tapering showed a small benefit over abrupt cessation by moderately reducing withdrawal symptoms. Tapering also showed a small benefit over abrupt cessation in the incidence of delirium. Two out of seventy participants experienced delirium, both following abrupt cessation. Although the incidence was low and the difference between interventions was nonsignificant, the CGC decided that the harms associated with delirium were sufficiently severe to warrant consideration. The CGC determined that the balance of effects probably favors a taper over abrupt cessation. The CGC decided the recommendation should be strong despite the low quality of evidence of effect, as CGC members agreed that the 1-week tapers included in the research evidence might be too rapid to see a significant benefit over abrupt cessation. In addition, the CGC agreed that patients place high value on reducing the severity of withdrawal symptoms.  EtD Table 1 References Summary   1. Petrovic M, Pevernagie D, Mariman A, Van Maele G, Afschrift M. Fast withdrawal from benzodiazepines in geriatric inpatients: a randomised double-blind, placebo-controlled trial. *Eur J Clin Pharmacol*. 2002;57(11):759-764. doi:10.1007/s00228-001-0387-4 2. Gerra G, Zaimovic A, Giusti F, Moi G, Brewer C. Intravenous flumazenil versus oxazepam tapering in the treatment of benzodiazepine withdrawal: a randomized, placebo-controlled study. *Addict Biol*. 2002;7(4):385-395. doi:10.1080/1355621021000005973 |

## EtD Table 2. Cognitive Behavioral Therapy for Indicated Condition + Taper Compared to Taper Alone for Benzodiazepine Discontinuation

In patients who are initiating a gradual taper to discontinue their long-term BZD use, does CBT that targets a specific underlying psychological condition (eg, CBT-I, CBT for GAD) result in better BZD reduction and clinical outcomes than tapering alone?

### Brief Evidence Summary

This EtD table supports Key Question 1. The systematic review identified six RCTs with 279 participants that compared CBT interventions for specific conditions plus a gradual BZD taper to a gradual BZD taper alone. Four RCTs had a high risk of bias from lack of blinding (Baillargeon et al [2003]; Morin et al [2004]; Otto et al [1993]; Otto et al [2010]), and two had an unclear risk of bias from partial blinding (Gosselin et al [2006]; Spiegel et al, [1994]). Three CBT interventions targeted panic disorder (Otto et al [1993]; Otto et al [2010]; Spiegel et al [1994]), two targeted insomnia (Baillargeon et al [2003]; Morin et al [2004]), and one targeted GAD (Gosselin et al [2006]). There was a higher rate of complete BZD discontinuation immediately after and up to 12 months following taper in the CBT + Taper groups compared to Taper alone (Baillargeon et al [2003]; Gosselin et al [2006]; Morin et al [2004]; Otto et al [1993]; Otto et al [2010]; Spiegel et al [1994]). Although the results were mixed on the rate of return to BZD use after a period of cessation, likely because of the significant heterogeneity at different time points, the overall pattern favors CBT + Taper.

### Summary of Findings Table

| **Certainty Assessment** | | | | | | | **№ of Patients** | | **Effect** | | **Certainty** | **Importance** |
| --- | --- | --- | --- | --- | --- | --- | --- | --- | --- | --- | --- | --- |
| **№ of Studies** | **Study Design** | **Risk of Bias** | **Inconsistency** | **Indirectness** | **Imprecision** | **Other Consider-ations** | **CBT for Indicated Condition + Taper** | **Taper** | **Relative (95% CI)** | **Absolute (95% CI)** |  |  |
| **BZD discontinuation @ 0–4 weeks post-taper** | | | | | | | | | | | | |
| 6^1-6^ | randomized trials | serious^a^ | not serious | not serious | not serious | none | 103/136 (75.7%) | 57/142 (40.1%) | **RR 1.86** (1.48 to 2.32) | **345 more per 1,000** (from 193 more to 530 more) | ⨁⨁⨁◯ Moderate | CRITICAL |
| **BZD discontinuation @ 2–4-month follow-up** | | | | | | | | | | | | |
| 6^1-6^ | randomized trials | serious^a^ | not serious | not serious | not serious | none | 89/136 (65.4%) | 47/142 (33.1%) | **RR 1.88** (1.48 to 2.43) | **291 more per 1,000** (from 159 more to 473 more) | ⨁⨁⨁◯ Moderate | CRITICAL |
| **BZD discontinuation @ 12–14-month follow-up** | | | | | | | | | | | | |
| 3^1,3,6^ | randomized trials | serious^a^ | serious^b^ | not serious | not serious | none | 59/92 (64.1%) | 29/85 (34.1%) | **RR 1.88** (1.35 to 2.64) | **300 more per 1,000** (from 119 more to 560 more) | ⨁⨁◯◯ Low | CRITICAL |
| **Return to BZD use @ 3-month follow-up** | | | | | | | | | | | | |
| 4^1,3-5^ | randomized trials | not serious | serious^c^ | not serious | serious^d^ | none | 10/67 (14.9%) | 8/36 (22.2%) | **Peto OR 0.60** (0.21 to 1.74) | **70 fewer per 1,000** (from 230 fewer to 80 more)^e^ | ⨁⨁◯◯ Low | CRITICAL |
| **Return to BZD use @ 6-month follow-up** | | | | | | | | | | | | |
| 2^3,4^ | randomized trials | not serious | not serious | not serious | serious^f^ | none | 3/33 (9.1%) | 8/19 (42.1%) | **Peto OR 0.15** (0.04 to 0.58) | **330 fewer per 1,000** (from 580 fewer to 90 fewer)^e^ | ⨁⨁⨁◯ Moderate | CRITICAL |
| **Return to BZD use @ 12-month follow-up** | | | | | | | | | | | | |
| 2^1,3^ | randomized trials | not serious | serious^g^ | not serious | very serious^h^ | none | 10/44 (22.7%) | 7/24 (29.2%) | **RR 0.78** (0.34 to 1.77) | **64 fewer per 1,000** (from 192 fewer to 225 more) | ⨁⨁◯◯ Low | CRITICAL |
| **BZD dose reduced 50% or more from baseline @ 0–4 weeks post-taper** | | | | | | | | | | | | |
| 1^6^ | randomized trials | serious^i^ | not serious | not serious | serious^f^ | none | 33/34 (97.1%) | 20/29 (69.0%) | **RR 1.41** (1.09 to 1.81) | **283 more per 1,000** (from 62 more to 559 more) | ⨁⨁◯◯ Low | IMPORTANT |
| **BZD dose reduced 50% or more from baseline @ 3-month follow-up** | | | | | | | | | | | | |
| 1^6^ | randomized trials | serious^i^ | not serious | not serious | very serious^h^ | none | 25/34 (73.5%) | 19/29 (65.5%) | **RR 1.12** (0.91 to 1.56) | **79 more per 1,000** (from 59 fewer to 367 more) | ⨁⨁◯◯ Low | IMPORTANT |
| **BZD dose @ 0–4 weeks post-taper (assessed in: mg/week diazepam equivalents)** | | | | | | | | | | | | |
| 2^1,3^ | randomized trials | serious^i^ | not serious | not serious | serious^d^ | none | 58 | 55 | — | MD **4.49 mg/week fewer** (17.83 fewer to 8.85 more) | ⨁⨁◯◯ Low | IMPORTANT |
| **BZD use frequency @ end of taper** | | | | | | | | | | | | |
| 1^1^ | randomized trials | serious^i^ | not serious | not serious | serious^f^ | none | 23 | 25 | — | MD **2.09 nights/week fewer** (3.35 fewer to 0.83 fewer) | ⨁⨁◯◯ Low | IMPORTANT |
| **BZD use frequency @ 3-month follow-up** | | | | | | | | | | | | |
| 1^1^ | randomized trials | serious^i^ | not serious | not serious | very serious^h^ | none | 27 | 25 | — | MD **0.7 nights/week fewer** (2 fewer to 0.6 more) | ⨁⨁◯◯ Low | IMPORTANT |
| **Withdrawal severity score @ 0–2 weeks post-taper (assessed with: PhWC, CIWA-B)** | | | | | | | | | | | | |
| 2^2,3^ | randomized trials | not serious | not serious | not serious | very serious^h^ | none | 40 | 43 | — | SMD **0.28 SD higher** (0.15 lower to 0.71 higher) | ⨁⨁◯◯ Low | IMPORTANT |
| **Anxiety score @ 2-week follow-up (assessed with: PSWQ)** | | | | | | | | | | | | |
| 1^3^ | randomized trials | not serious | not serious | not serious | serious^f^ | none | 27 | 26 | — | MD **5.63 lower** (9.72 lower to 1.54 lower) | ⨁⨁⨁◯ Moderate | IMPORTANT |
| **Anxiety score @ 3-month follow-up (assessed with: PSWQ)** | | | | | | | | | | | | |
| 1^3^ | randomized trials | not serious | not serious | not serious | serious^f^ | none | 27 | 27 | — | MD **6.11 lower** (10.77 lower to 1.45 lower) | ⨁⨁⨁◯ Moderate | IMPORTANT |
| **Persistence of GAD symptoms @ 2-week follow-up (assessed with: ADIS-IV)** | | | | | | | | | | | | |
| 1^3^ | randomized trials | not serious | not serious | not serious | serious^f^ | none | 11/31 (35.5%) | 24/30 (80.0%) | **RR 0.44** (0.27 to 0.74) | **448 fewer per 1,000** (from 584 fewer to 208 fewer) | ⨁⨁⨁◯ Moderate | CRITICAL |
| **Persistence of GAD symptoms @ 3-month follow-up (assessed with: ADIS-IV)** | | | | | | | | | | | | |
| 1^3^ | randomized trials | not serious | not serious | not serious | serious^f^ | none | 10/31 (32.3%) | 18/30 (60.0%) | **RR 0.54** (0.30 to 0.97) | **276 fewer per 1,000** (from 420 fewer to 18 fewer) | ⨁⨁⨁◯ Moderate | CRITICAL |
| **Persistence of GAD symptoms @ 6-month follow-up (assessed with: ADIS-IV)** | | | | | | | | | | | | |
| 1^3^ | randomized trials | not serious | not serious | not serious | very serious^h^ | none | 12/31 (38.7%) | 16/30 (53.3%) | **RR 0.73** (0.42 to 1.26) | **144 fewer per 1,000** (from 309 fewer to 139 more) | ⨁⨁◯◯ Low | CRITICAL |
| **Sleep problem score @ end of taper (assessed with: ISI)** | | | | | | | | | | | | |
| 2^1,3^ | randomized trials | not serious | not serious | not serious | not serious | none | 55 | 53 | — | MD **2.04 lower** (4 lower to 0.08 lower) | ⨁⨁⨁⨁ High | IMPORTANT |
| **Sleep problem score @ 3-month follow-up (assessed with: ISI)** | | | | | | | | | | | | |
| 2^1,3^ | randomized trials | serious^i^ | not serious | not serious | serious^d^ | none | 55 | 53 | — | MD **0.17 higher** (2.04 lower to 2.38 higher) | ⨁⨁◯◯ Low | IMPORTANT |
| **Serious adverse events** | | | | | | | | | | | | |
| 1^6^ | randomized trials | serious^a^ | not serious | not serious | very serious^h^ | none | 0/35 (0.0%) | 0/30 (0.0%) | **RD 0.00** (-0.06 to 0.06) | **0 fewer per 1,000** (from 60 fewer to 60 more)^e^ | ⨁⨁◯◯ Low | CRITICAL |
| **Dropout** | | | | | | | | | | | | |
| 5^1-4,6^ | randomized trials | serious^a^ | not serious | not serious | serious^d^ | none | 7/120 (5.8%) | 11/126 (8.7%) | **Peto OR 0.51** (0.24 to 1.08) | **80 fewer per 1,000** (from 160 fewer to 10 more)^e^ | ⨁⨁◯◯ Low | CRITICAL |

**ADIS-IV:** Anxiety Disorders Interview Schedule for DSM–IV; **BZD:** benzodiazepine; **CBT:** cognitive behavioral therapy; **CI:** confidence interval; **CIWA-B**: Clinical Institute Withdrawal Assessment – Benzodiazepines, score range unclear, higher = more severe, physician and patient rated; **GAD:** generalized anxiety disorder; **ISI:** Insomnia Severity Index, score range 0–28, higher = more sleep difficulty; **MD:** mean difference; **PhWC:** Physician Withdrawal Checklist, score range unclear, higher = more severe; **PSWQ:** Penn State Worry Questionnaire, score range unclear, scale direction unclear; **OR:** odds ratio; **RD:** risk difference; **RR:** risk ratio; **SD:** standard deviation; **SMD:** standardized mean difference

**GRADE Working Group Grades of Evidence**

High quality: Further research is very unlikely to change our confidence in the estimate of effect.

Moderate quality: Further research is likely to have an important impact on our confidence in the estimate of effect and may change the estimate.

Low quality: Further research is very likely to have an important impact on our confidence in the estimate of effect and is likely to change the estimate.

Very low quality: We are very uncertain about the estimate.

Note: significant heterogeneity defined as p = <0.10.

**Explanations**

- 1. High risk of performance bias from lack of blinding for a majority of participants.
  2. Significant heterogeneity (I² = 65%, p = 0.06).
  3. Significant heterogeneity (I² = 74%, p = 0.01).
  4. 95% CI crosses the line of null effect.
  5. Absolute effect calculated from the risk difference (RD) due to zero events in one or both arms.
  6. Small sample size (n = <100).
  7. Significant heterogeneity (I² = 67%, p = 0.08).
  8. Small sample size (n = <100) and 95% CI crosses the line of null effect.
  9. High risk of performance and detection bias for unblinded subjective measures for a majority of participants.

### Question

| **Should CBT for Indicated Condition + Taper or Taper be used for patients discontinuing long-term BZD use?** | |
| --- | --- |
| **POPULATION** | Patients discontinuing long-term BZD use |
| **INTERVENTION** | CBT for indicated condition (eg, CBT-I, CBT for GAD) + Taper |
| **COMPARISON** | Taper |
| **MAIN OUTCOMES** | BZD discontinuation; return to BZD use after a period of cessation; BZD dose; BZD frequency; withdrawal severity score; anxiety score; persistence of GAD symptoms; sleep problem score; serious adverse events; dropout |
| **SETTING** | Any clinical setting where BZD tapering occurs |
| **PERSPECTIVE** | Patient-level |
| **CONFLICT OF INTERESTS** | None identified. |

### Assessment

| **Problem**  Is the problem a priority? | | |
| --- | --- | --- |
| Judgment | Research Evidence | Additional Considerations |
| ○ No  ○ Probably no  ○ Probably yes  ● Yes  ○ Varies  ○ Unknown |  |  |
| **Desirable Effects**  How substantial are the desirable anticipated effects? | | |
| Judgment | Research Evidence | Additional Considerations |
| ○ Trivial  ● Small  ○ Moderate  ○ Large  ○ Varies  ○ Unknown | CBT + Taper showed a benefit compared to Taper alone in a majority of critical and important outcomes. CBT + Taper increased BZD discontinuation rates and significant dose reductions, decreased the persistence of GAD, and may decrease return to BZD use after discontinuation. CBT + Taper also decreased the severity of anxiety symptoms and may decrease sleep problems. Taper alone may be slightly favored in decreasing withdrawal severity, but this effect is very uncertain. | The same outcome (BZD discontinuation, return to BZD use) has multiple time points. However, all time points favor CBT + Taper over Taper. |
| **Undesirable Effects**  How substantial are the undesirable anticipated effects? | | |
| Judgment | Research Evidence | Additional Considerations |
| ○ Trivial  ● Small  ○ Moderate  ○ Large  ○ Varies  ○ Unknown | Neither intervention is favored in critical undesirable effects; no serious adverse events were reported. CBT + Taper was favored in one important negative effect: dropout was lower in the CBT + Taper group. |  |
| **Certainty of Evidence**  What is the overall certainty of the evidence of effects? | | |
| Judgment | Research Evidence | Additional Considerations |
| ○ Very low  ● Low  ○ Moderate  ○ High  ○ No included studies | \| **Outcomes** \| **Importance** \| **Certainty of the Evidence (GRADE)** \| \| --- \| --- \| --- \| \| BZD discontinuation @ 0–4 weeks post-taper \| CRITICAL \| ⨁⨁⨁◯ Moderate^a^ \| \| BZD discontinuation @ 2–4-month follow-up \| CRITICAL \| ⨁⨁⨁◯ Moderate^a^ \| \| BZD discontinuation @ 12–14-month follow-up \| CRITICAL \| ⨁⨁◯◯ Low^a,b^ \| \| Return to BZD use @ 3-month follow-up \| CRITICAL \| ⨁⨁◯◯ Low^c,d^ \| \| Return to BZD use @ 6-month follow-up \| CRITICAL \| ⨁⨁⨁◯ Moderate^e^ \| \| Return to BZD use @ 12-month follow-up \| CRITICAL \| ⨁⨁◯◯ Low^f,g^ \| \| BZD dose reduced 50% or more @ 0–4 weeks post-taper \| IMPORTANT \| ⨁⨁◯◯ Low^e,h^ \| \| BZD dose reduced 50% or more @ 3-month follow-up \| IMPORTANT \| ⨁⨁◯◯ Low^g,h^ \| \| BZD dose @ 0–4 weeks post-taper  (assessed with: mg/week diazepam equivalents) \| IMPORTANT \| ⨁⨁◯◯ Low^d,h^ \| \| BZD frequency @ end of taper \| IMPORTANT \| ⨁⨁◯◯ Low^e,h^ \| \| BZD frequency @ 3-month follow-up \| IMPORTANT \| ⨁⨁◯◯ Low^g,h^ \| \| Withdrawal severity score @ 0–2 weeks post-taper  (assessed with: PhWC, CIWA-B) \| IMPORTANT \| ⨁⨁◯◯ Low^g^ \| \| Anxiety score @ 2-week follow-up  (assessed with: PSWQ) \| IMPORTANT \| ⨁⨁⨁◯ Moderate^e^ \| \| Anxiety score @ 3-month follow-up  (assessed with: PSWQ) \| IMPORTANT \| ⨁⨁⨁◯ Moderate^e^ \| \| Persistence of GAD symptoms @ 2-week follow-up  (assessed with: ADIS-IV) \| CRITICAL \| ⨁⨁⨁◯ Moderate^e^ \| \| Persistence of GAD symptoms @ 3-month follow-up  (assessed with: ADIS-IV) \| CRITICAL \| ⨁⨁⨁◯ Moderate^e^ \| \| Persistence of GAD symptoms @ 6-month follow-up  (assessed with: ADIS-IV) \| CRITICAL \| ⨁⨁◯◯ Low^g^ \| \| Sleep problem score @ end of taper  (assessed with: ISI) \| IMPORTANT \| ⨁⨁⨁⨁ High \| \| Sleep problem score @ 3-month follow-up  (assessed with: ISI) \| IMPORTANT \| ⨁⨁◯◯ Low^d,h^ \| \| Serious adverse events \| CRITICAL \| ⨁⨁◯◯ Low^a,g^ \| \| Dropout \| CRITICAL \| ⨁⨁◯◯ Low^a,d^ \|  1. High risk of performance bias from lack of blinding for a majority of participants. 2. Significant heterogeneity (I² = 65%, p = 0.06). 3. Significant heterogeneity (I² = 74%, p = 0.01). 4. 95% CI crosses the line of null effect. 5. Small sample size (n = <100). 6. Significant heterogeneity (I² = 67%, p = 0.08). 7. Small sample size (n = <100) and 95% CI crosses the line of null effect. 8. High risk of performance and detection bias for unblinded subjective measures for a majority of participants. |  |
| **Values**  Is there important uncertainty about or variability in how much people value the main outcomes? | | |
| Judgment | Research Evidence | Additional Considerations |
| ○ Important uncertainty or variability  ○ Possibly important uncertainty or variability  ● Probably no important uncertainty or variability  ○ No important uncertainty or variability | There was no evidence in the literature review about values and preferences of outcomes.  Outcomes included BZD discontinuation, return to BZD use, BZD dose reduction, weekly BZD frequency, withdrawal severity score, recurrence or persistence of indicated condition (eg, GAD), sleep problem score, and serious adverse events. | Variability likely exists across the patient population, but direct research evidence is lacking. |
| **Balance of Effects**  Does the balance between desirable and undesirable effects favor the intervention or the comparison? | | |
| Judgment | Research Evidence | Additional Considerations |
| ○ Favors the comparison  ○ Probably favors the comparison  ○ Does not favor either the intervention or the comparison  ● Probably favors the intervention  ○ Favors the intervention  ○ Varies  ○ Unknown | Both the desirable and undesirable effects favor CBT + Taper. |  |
| **Resources Required**  How large are the resource requirements (ie, costs)? | | |
| Judgment | Research Evidence | Additional Considerations |
| ○ Large costs  ○ Moderate costs  ○ Negligible costs and savings  ○ Moderate savings  ○ Large savings  ● Varies  ○ Unknown |  |  |
| **Cost Effectiveness**  Does the cost-effectiveness of the intervention favor the intervention or the comparison? | | |
| Judgment | Research Evidence | Additional Considerations |
| ○ Favors the comparison  ○ Probably favors the comparison  ○ Does not favor either the intervention or the comparison  ○ Probably favors the intervention  ○ Favors the intervention  ● Varies  ○ No included studies |  |  |
| **Acceptability**  Is the intervention acceptable to key stakeholders? | | |
| Judgment | Research Evidence | Additional Considerations |
| ○ No  ○ Probably no  ○ Probably yes  ○ Yes  ● Varies  ○ Unknown | An Australian survey conducted at pharmacies (Sake et al [2019])^7^ reported that 48 of 75 participants did not prefer behavioral therapies for various reasons such as lack of confidence in behavioral therapies, lack of time, dependency on sleeping pills, participants’ perception that behavioral therapies take longer to produce effects, participants’ perception that seeing a psychologist is costly, and other undefined reasons (participants were allowed to select multiple answers). |  |
| **Feasibility**  Is the intervention feasible to implement? | | |
| Judgment | Research Evidence | Additional Considerations |
| ○ No  ○ Probably no  ○ Probably yes  ○ Yes  ● Varies  ○ Unknown | The CGC acknowledges that CBT is not accessible in all geographic locations. The availability of high-quality in-person CBT is likely low. Adequate training and experience of therapists is necessary. Online CBT resources are more easily available, but quality may be difficult to assess. Feasibility may vary on geographic location. |  |

### Summary of Judgments

|  | **JUDGMENT** | | | | | | |
| --- | --- | --- | --- | --- | --- | --- | --- |
| **PROBLEM** | No | Probably no | Probably yes | **Yes** |  | Varies | Unknown |
| **DESIRABLE EFFECTS** | Trivial | **Small** | Moderate | Large |  | Varies | Unknown |
| **UNDESIRABLE EFFECTS** | Trivial | **Small** | Moderate | Large |  | Varies | Unknown |
| **CERTAINTY OF EVIDENCE** | Very low | **Low** | Moderate | High |  |  | No included studies |
| **VALUES** | Important uncertainty or variability | Possibly important uncertainty or variability | **Probably no important uncertainty or variability** | No important uncertainty or variability |  |  |  |
| **BALANCE OF EFFECTS** | Favors the comparison | Probably favors the comparison | Does not favor either the intervention or the comparison | **Probably favors the intervention** | Favors the intervention | Varies | Unknown |
| **RESOURCES REQUIRED** | Large costs | Moderate costs | Negligible costs and savings | Moderate savings | Large savings | **Varies** | Unknown |
| **CERTAINTY OF EVIDENCE OF REQUIRED RESOURCES** | Very low | Low | Moderate | High |  |  | **No included studies** |
| **COST EFFECTIVENESS** | Favors the comparison | Probably favors the comparison | Does not favor either the intervention or the comparison | Probably favors the intervention | Favors the intervention | **Varies** | No included studies |
| **EQUITY** | Reduced | Probably reduced | Probably no impact | Probably increased | Increased | **Varies** | Unknown |
| **ACCEPTABILITY** | No | Probably no | Probably yes | Yes |  | **Varies** | Unknown |
| **FEASIBILITY** | No | Probably no | Probably yes | Yes |  | **Varies** | Unknown |

### Type of Recommendation

| Strong recommendation against the intervention | Conditional recommendation against the intervention | Conditional recommendation for either the intervention or the comparison | Conditional recommendation for the intervention | **Strong recommendation for the intervention** |
| --- | --- | --- | --- | --- |
| ○ | ○ | ○ | ○ | ● |

### Conclusions

| **Recommendation** |
| --- |
| **Recommendation 10:** Clinicians should offer patients undergoing BZD tapering behavioral interventions tailored to their underlying conditions (eg, CBT, CBT-I) or provide them with referrals to access these interventions (Low Certainty, Strong Recommendation). |
| **Justification** |
| The evidence of treatment effect is very uncertain due to the small size and high risk of bias in most studies evaluated. The evidence consistently showed a benefit of CBT + Taper compared to Taper alone in most critical outcomes such that the balance of desirable and undesirable effects probably favors CBT + Taper. The CGC acknowledges the potential limitations in patient acceptability and provider feasibility.  EtD Table 2 References Summary   1. Morin CM, Bastien C, Guay B, Radouco-Thomas M, Leblanc J, Vallières A. Randomized clinical trial of supervised tapering and cognitive behavior therapy to facilitate benzodiazepine discontinuation in older adults with chronic insomnia. *AJP*. 2004;161(2):332-342. doi:10.1176/appi.ajp.161.2.332 2. Otto MW, McHugh RK, Simon NM, Farach FJ, Worthington JJ, Pollack MH. Efficacy of CBT for benzodiazepine discontinuation in patients with panic disorder: further evaluation. *Behav Res Ther*. 2010;48(8):720-727. 3. Gosselin P, Ladouceur R, Morin CM, Dugas MJ, Baillargeon L. Benzodiazepine discontinuation among adults with GAD: a randomized trial of cognitive-behavioral therapy. *J Consult Clin Psychol*. 2006;74(5):908-919. doi:10.1037/0022-006X.74.5.908 4. Spiegel DA, Bruce TJ, Gregg SF, Nuzzarello A. Does cognitive behavior therapy assist slow-taper alprazolam discontinuation in panic disorder? *Am J Psychiatry*. 1994;151:876-881. 5. Otto MW, Pollack MH, Sachs GS, Reiter SR, Meltzer-Brody S, Rosenbaum JF. Discontinuation of benzodiazepine treatment: efficacy of cognitive-behavioral therapy*. Am J Psychiatry*. 1993;150(10):1485-1490. 6. Baillargeon L, Landreville P, Verreault R, Beauchemin JP, Grégoire JP, Morin CM. Discontinuation of benzodiazepines among older insomniac adults treated with cognitive-behavioural therapy combined with gradual tapering: a randomized trial. *CMAJ*. 2003;169(10):1015-1020. 7. Sake FT, Wong K, Bartlett DJ, Saini B. Benzodiazepine usage and patient preference for alternative therapies: A descriptive study. *Health Sci Rep*. 2019;2(5):e116. doi:10.1002/hsr2.116 |

## EtD Table 3. Phenobarbital Compared to No Taper for Benzodiazepine Discontinuation

### Brief Evidence Summary

This EtD table supports Key Question 1. The systematic review identified two retrospective chart reviews with 665 participants that reviewed the outcomes of a phenobarbital taper for benzodiazepine detoxification. Both chart reviews had a high risk of bias from their observational nature (Kawasaki et al [2012]; Sartori et al [2022]). Both chart reviews (Kawasaki et al [2012]; Sartori et al [2022]) had a high amount of success in tapering patients with minimal adverse events.

### Summary of Findings Table

| Certainty assessment | | | | | | | Impact | Certainty | Importance |
| --- | --- | --- | --- | --- | --- | --- | --- | --- | --- |
| № of studies | Study design | Risk of bias | Inconsistency | Indirectness | Imprecision | Other considerations |  |  |  |
| BZD Cessation, immediately following rapid phenobarbital taper | | | | | | | | | |
| 2^1,2^ | non-randomised studies | very serious^a^ | not serious | not serious | not serious | none | 540/665 (81.2%) were BZD free after phenobarbital taper. Of the patients that completed the taper and were not BZD free (125/355, 35.2%) they tapered their BZD in outpatient. | ⨁⨁◯◯ Low^a^ | CRITICAL |
| Adverse Events | | | | | | | | | |
| 2^1,2^ | non-randomised studies | very serious^a^ | not serious | not serious | not serious | none | 4/665 (0.6%) had adverse events during phenobarbital taper. Adverse events included delirium 3/310 (Kawasaki 2012) and a skin rash 1/355 (Sartori 2022). | ⨁⨁◯◯ Low^a^ | CRITICAL |
| Left against medical advice | | | | | | | | | |
| 2^1,2^ | non-randomised studies | very serious^a^ | not serious | not serious | not serious | none | 72/665 (10.8%) left against medical advice | ⨁⨁◯◯ Low^a^ | CRITICAL |

**BZD:** benzodiazepine **CI:** confidence interval

**GRADE Working Group Grades of Evidence**

High quality: Further research is very unlikely to change our confidence in the estimate of effect.

Moderate quality: Further research is likely to have an important impact on our confidence in the estimate of effect and may change the estimate.

Low quality: Further research is very likely to have an important impact on our confidence in the estimate of effect and is likely to change the estimate.

Very low quality: We are very uncertain about the estimate.

**Explanations**

1. Retrospective chart review. Unclear how patients were selected into the chart review

### Question

| **Should Phenobarbital vs. Not tapering be used for BZD discontinuation?** | |
| --- | --- |
| **Population:** | BZD discontinuation |
| **Intervention:** | Phenobarbital taper |
| **Comparison:** | No taper |
| **Main outcomes:** | BZD Cessation, Adverse Events, Left against medical advice. |
| **Setting:** | Inpatient setting where a phenobarbital taper is done. |
| **Perspective:** | Patient-level |
| **Conflict of interests:** | None identified. |

### Assessment

| Problem  Is the problem a priority? | | |
| --- | --- | --- |
| Judgement | Research evidence | Additional considerations |
| ○ No ○ Probably no ○ Probably yes ● Yes ○ Varies ○ Don't know |  |  |
| Desirable Effects  How substantial are the desirable anticipated effects? | | |
| Judgement | Research evidence | Additional considerations |
| ○ Trivial ○ Small ● Moderate ○ Large ○ Varies ○ Don't know | Kawasaki et al 2012 was a chart review of 310 patients that had a 3-day fixed-dose phenobarbital taper for BZD dependence. All 310 patients were tapered and only 3/310 (1%) had a readmission for treatment of BZD withdrawal.  Sartori et al 2022 was a chart review of 355 patients that had a phenobarbital detoxification. The patients were hospitalized for treatment, and at discharge 230/355 (64.8%) were BZD free, while the rest of the patients 125/355 (35.2%) had a supportive BZD therapy that was tapered in outpatient. | Messinger et al 2023 was a case study (n = 1) where a patient was rapidly tapered (per their request) and given a large dose of intravenous phenobarbital (650 mg) at the end of the taper. Patient remained hospitalized for a few days following and was released and remained BZD-free at the latest follow-up (60-days). |
| Undesirable Effects  How substantial are the undesirable anticipated effects? | | |
| Judgement | Research evidence | Additional considerations |
| ○ Trivial ● Small ○ Moderate ○ Large ○ Varies ○ Don't know | Kawasaki et al 2012: Adverse events included 3/310 (1%) had delirium and 22/310 (7.1%) had an ED visit within 30 days after taper.  Sartori et al 2022: Adverse events included 1/355 that got a skin rash and discontinued phenobarbital. |  |
| Certainty of evidence  What is the overall certainty of the evidence of effects? | | |
| Judgement | Research evidence | Additional considerations |
| ○ Very low ● Low ○ Moderate ○ High ○ No included studies | \| **Outcomes** \| **Importance** \| **Certainty of the evidence (GRADE)** \| \| --- \| --- \| --- \| \| BZD Cessation, immediately following rapid phenobarbital taper \| CRITICAL \| ⨁⨁◯◯ Low^a^ \| \| Adverse Events \| CRITICAL \| ⨁⨁◯◯ Low^a^ \| \| Left against medical advice \| CRITICAL \| ⨁⨁◯◯ Low^a^ \|  1. Retrospective chart review. Unclear how patients were selected into the chart review. |  |
| Values  Is there important uncertainty about or variability in how much people value the main outcomes? | | |
| Judgement | Research evidence | Additional considerations |
| ○ Important uncertainty or variability ○ Possibly important uncertainty or variability ● Probably no important uncertainty or variability ○ No important uncertainty or variability | There was no evidence in the literature review about values and preferences of outcomes.  Outcomes included BZD cessation, adverse events, and left against medical advice. | Variability likely exists across the patient population, but direct research evidence is lacking. |
| Balance of effects  Does the balance between desirable and undesirable effects favor the intervention or the comparison? | | |
| Judgement | Research evidence | Additional considerations |
| ○ Favors the comparison ○ Probably favors the comparison ○ Does not favor either the intervention or the comparison ● Probably favors the intervention ○ Favors the intervention ○ Varies ○ Don't know | Both the desirable and undesirable effects favor phenobarbital taper. |  |
| Resources required  How large are the resource requirements (ie, costs)? | | |
| Judgement | Research evidence | Additional considerations |
| ○ Large costs ○ Moderate costs ○ Negligible costs and savings ○ Moderate savings ○ Large savings ● Varies ○ Don't know |  |  |
| Cost effectiveness  Does the cost-effectiveness of the intervention favor the intervention or the comparison? | | |
| Judgement | Research evidence | Additional considerations |
| ○ Favors the comparison ○ Probably favors the comparison ○ Does not favor either the intervention or the comparison ○ Probably favors the intervention ○ Favors the intervention ● Varies ○ No included studies |  |  |
| Acceptability  Is the intervention acceptable to key stakeholders? | | |
| Judgement | Research evidence | Additional considerations |
| ○ No ○ Probably no ● Probably yes ○ Yes ○ Varies ○ Don't know | Kawasaki et al 2012: 53/310 (17.1%) left against medical advice.  Sartori et al 2022: 19/355 (5.3%) left against medical advice.  Total: 72/665 (10.8%) |  |
| Feasibility  Is the intervention feasible to implement? | | |
| Judgement | Research evidence | Additional considerations |
| ○ No ○ Probably no ● Probably yes ○ Yes ○ Varies ○ Don't know |  |  |

### Summary of Judgements

|  | **Judgement** | | | | | | |
| --- | --- | --- | --- | --- | --- | --- | --- |
| **Problem** | No | Probably no | Probably yes | **Yes** |  | Varies | Don't know |
| **Desirable Effects** | Trivial | Small | **Moderate** | Large |  | Varies | Don't know |
| **Undesirable Effects** | Trivial | **Small** | Moderate | Large |  | Varies | Don't know |
| **Certainty of evidence** | Very low | **Low** | Moderate | High |  |  | No included studies |
| **Values** | Important uncertainty or variability | Possibly important uncertainty or variability | **Probably no important uncertainty or variability** | No important uncertainty or variability |  |  |  |
| **Balance of effects** | Favors the comparison | Probably favors the comparison | Does not favor either the intervention or the comparison | **Probably favors the intervention** | Favors the intervention | Varies | Don't know |
| **Resources required** | Large costs | Moderate costs | Negligible costs and savings | Moderate savings | Large savings | **Varies** | Don't know |
| **Cost effectiveness** | Favors the comparison | Probably favors the comparison | Does not favor either the intervention or the comparison | Probably favors the intervention | Favors the intervention | **Varies** | No included studies |
| **Equity** | Reduced | Probably reduced | Probably no impact | Probably increased | Increased | **Varies** | Don't know |
| **Acceptability** | No | Probably no | **Probably yes** | Yes |  | Varies | Don't know |
| **Feasibility** | No | Probably no | **Probably yes** | Yes |  | Varies | Don't know |

### Type of Recommendation

| Strong recommendation against the intervention | Conditional recommendation against the intervention | Conditional recommendation for either the intervention or the comparison | Conditional recommendation for the intervention | **Strong recommendation for the intervention** |
| --- | --- | --- | --- | --- |
| ○ | ○ | ○ | ○ | **●** |
|  |  |  |  |  |

### Conclusions

| Recommendation |
| --- |
| Recommendation 13. Tapering with very long-acting agents such as phenobarbital:  a. Can be considered for BZD withdrawal management in inpatient settings (Low Certainty, Strong Recommendation). |
|  |
| Justification |
|  |

EtD Table 3 References Summary

1. Kawasaki SS, Jacapraro JS, Rastegar DA. Safety and effectiveness of a fixed-dose phenobarbital protocol for inpatient benzodiazepine detoxification. *J Subst Abuse Treat*. 2012;43(3):331-334. doi:10.1016/j.jsat.2011.12.011
2. Sartori S, Crescioli G, Brilli V, et al. Phenobarbital use in benzodiazepine and z-drug detoxification: a single-centre 15-year observational retrospective study in clinical practice. *Intern Emerg Med*. 2022;17(6):1631-1640. doi:10.1007/s11739-022-02976-0
